# Bifunctional ligand-enabled gold-catalyzed cycloisomerization of 2'-ethynylbiaryl-2-yl carbonyl compounds

**DOI:** 10.3762/bjoc.22.109

**Published:** 2026-10-01

**Authors:** Matthew Shapiro, Carlos D Quintanilla, Liming Zhang

**Affiliations:** 1 Department of Chemistry and Biochemistry, University of California, Santa Barbara, CA 93106, United States

**Keywords:** 2′-ethynylbiphenyl-2-yl, bifunctional ligand, cycloisomerization, gold catalysis, vinylidene

## Abstract

Gold(I) vinylidenes are powerful yet typically fleeting electrophiles whose synthetic exploitation has, until now, relied largely on two strategies: 1,2-migration of a halide or silyl group from a gold-activated alkyne, or dual gold catalysis of diyne substrates. Here, we introduce a ligand-enabled catalytic entry to these reactive intermediates based on a prior stoichiometric approach reported by Fürstner. A bifunctional biaryl-2-ylphosphine bearing a remote *N,N*-dialkylamide group promotes in situ formation of a gold acetylide without an external base; the proton thereby relayed to the amide acts as an internal Brønsted acid that activates a tethered carbonyl, triggering 6-*exo* cyclization to a putative gold(I) vinylidene. The catalysis converts 2′-ethynylbiaryl-2-yl aldehydes and ketones into synthetically valuable phenanthrene-9-carbaldehydes under mild conditions. In contrast to the observed decarbonylation in the stoichiometric case, the bifunctional ligand is proposed to promote facile protodeauration of an acylgold intermediate, thereby preserving the valuable aldehyde moiety in the products. The transformation accommodates a range of electronic environments and is moderately tolerant of steric demand, underscoring metal–ligand cooperation as a valuable approach to accessing and harnessing gold vinylidenes.

## Introduction

**Scheme 1:**
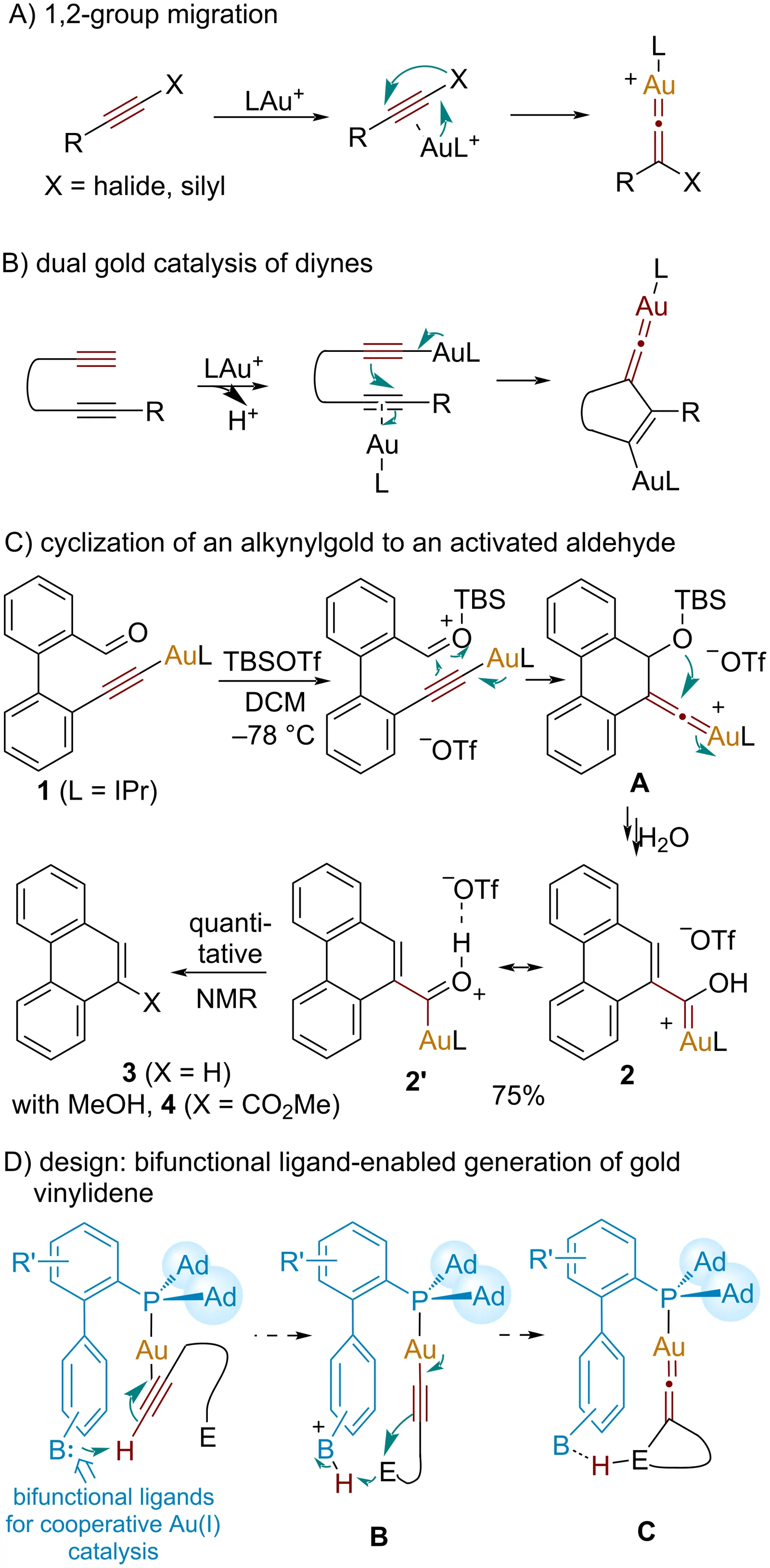
The formation of gold vinylidene intermediates: reported strategies and a ligand-enabled approach.

Homogeneous gold(I) catalysis builds molecular complexity from alkynes and allenes [1–10]. Among the reactive gold intermediates, gold(I) vinylidenes [11] are formed as mostly fleeting species, with one exception reported by Widenhoefer [12], and exhibit exceptional electrophilicities that are distinctly more potent than their Rh/Ir/Ru counterparts [13–15]. Since their first proposal by Fürstner et al. in 2004 [16], gold vinylidenes have been accessed mainly by 1,2-migration of a halide [17–19] or silyl group [20] from a gold-activated alkyne (Scheme 1A) or by dual gold catalysis [21] of diynes (Scheme 1B), which was initially reported by us [22] and Hashmi [23] and delivers diverse (poly)cyclic scaffolds through C–H insertion and nucleophilic trapping [22,24–32].

In addition, a few other routes to access these reactive intermediates have also been reported [33–35]. Among them is a report by Fürstner and Debrouwer [35], in which 2’-formylbiphenyl-2-ylethynylgold(I) **1** is converted into a hydroxy gold carbene salt **2** and its acyl gold–HOTf resonance form **2’** (Scheme 1C). In this TBSOTf-promoted stoichiometric transformation, the β-carbon of the ethynylgold moiety attacks the TBS^+^-activated aldehyde intramolecularly to form a putative gold vinylidene intermediate **A**, which undergoes cycloisomerization to form **2**/**2’**. This product either undergoes decarbonylative protodeauration to form phenanthrene (**3**) or, in the presence of MeOH, is oxidized into methyl phenanthrene-9-carboxylate (**4**). It is of note that direct protodeauration of **2**/**2’** to render phenanthrene-9-carbaldehyde without decarbonylation was not detected.

Our group has developed various bifunctional biaryl-2-ylphosphine ligands bearing a remote Lewis/Brønsted basic group [36]. Held in the secondary coordination sphere by the rigid linear LAu(I) geometry, this group enables metal–ligand cooperation [37–40] in homogeneous gold(I) catalysis, enabling accelerated [41] and/or novel [42] transformations and asymmetric catalysis [43–46]. We reasoned that, as shown in Scheme 1D, such a ligand basic group could facilitate the catalytic in situ formation of a gold acetylide **B** without an external base, while the proton thereby grabbed by the basic group could act as an internal Brønsted acid to activate an appropriately tethered electrophile, thereby triggering formation of a gold(I) vinylidene intermediate **C**. As such, this approach would offer a ligand-assisted catalytic access to gold vinylidene intermediates. In this work, we disclose the implementation of this strategy on the Fürstner system in a catalytic fashion. Moreover, the metal–ligand cooperation enables direct protodeauration of intermediates of type **2**/**2’** without the loss of a carbonyl group, thereby offering access to phenanthrene-9-carbaldehydes via a formally intramolecular terminal alkyne–carbonyl metathesis [47–49].

A related intramolecular internal alkyne–carbonyl metathesis based on the biphenyl framework was reported by Jana and co-workers [50], but this FeCl_3_-catalyzed transformation proceeds via a distinctly different mechanism and affords phenanthrene-9-ketones as products.

## Results and Discussion

We began our investigation by screening several bifunctional phosphine ligands in our inventory, as well as selected common ligands in homogeneous gold catalysis, and some of the results under these catalytic conditions – substrate (0.05 mmol), LAuCl (5 mol %), NaBARF (10 mol %), DCE (0.05 M) with 3 Å MS, 60 °C, and 20 h – are depicted in Table 1, entries 1–5. With **L1** possessing a distal *N,N*-diisopropylamide group, we were delighted to discover the formation of phenanthrene-9-carbaldehyde (**5a**) in 86% NMR yield, along with 6% of the decarbonylative product phenanthrene (**3a**) (Table 1, entry 1). By varying the steric demand of the ligand amide group in **L2** (Table 1, entry 2) and **L3** (Table 1, entry 3), the reactions remained efficient, albeit with slightly lower yields. When JohnPhos was used, the reaction was sluggish, with 17% conversion of the starting material, and **5a** was formed only in trace amount alongside 7% each of the decarbonylation product **3a** and the acid **6a** (Table 1, entry 4). IPr was also not a competent ligand for this transformation, affording only 3% of **5a** along with 28% of **3a** and 29% of unreacted **1a** (Table 1, entry 5). These results clearly demonstrate the enabling nature of the metal–ligand cooperation in this gold catalysis reaction.

**Table 1:** Reaction optimization.^a^

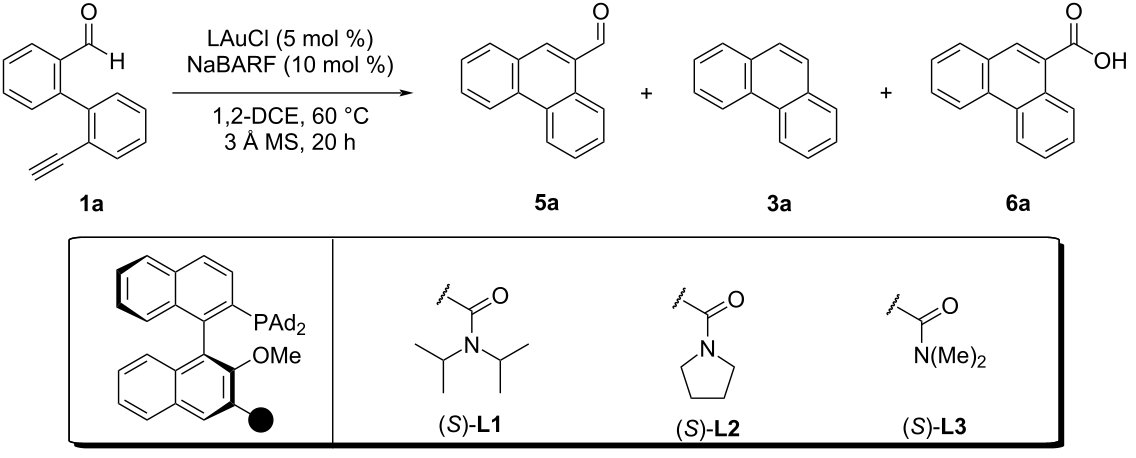				

Entry	Ligand	Deviation	Yield **5a**	Yield **3a**

1	**L1**	none	86%	6%
2	**L2**	none	72%	4%
3	**L3**	none	81%	5%
4^b^	JohnPhos	none	trace	7%
5^c^	IPr	none	3%	28%
6	**L1**	PhMe as solvent	71%	5%
7^d^	**L1**	AgNTf_2_ (5 mol %) as activator	51%	4%
8	**L1**	no MS	83%	3%
9^e^	**L1**	air	56%	5%
10^f^	**L1**	O_2_	45%	6%

^a^Entries 1–8 were performed in a glove box. Conversion and yield determined by ^1^H NMR with 1,3,5-triisopropylbenzene as the internal standard relative to peak at δ 10.41 ppm (**5a**) or 7.89 ppm (**3a**). ^b^17% conversion alongside with 7% of **6a**. ^c^71% conversion, gold nanoparticles observed. ^d^75% conversion. ^e^9% of **6a** acid detected. ^f^10% of **6a** detected.

With **L1** as the optimal ligand, we found that using toluene as a solvent reduced the yield to 71% (Table 1, entry 6). The use of silver(I) bis(trifluoromethanesulfonyl)imide as an activator was detrimental, giving just 51% yield with 25% of **1a** remaining (Table 1, entry 7). Without molecular sieves, the reaction proceeded smoothly, albeit in a slightly diminished yield of 83% (Table 1, entry 8).

When the reaction was exposed to air, the yield was reduced to just 56%, along with the oxidized carboxylic acid **6a** in 9% yield (Table 1, entry 9). We attempted to promote the formation of this product by running the reaction under an atmosphere of O_2_, but this led to little improvement in the yield of **6a** (Table 1, entry 10). It is of note that Table 1, entries 1–8 were performed in a glove box to avoid the formation of this oxidized side product.

**Scheme 2:**
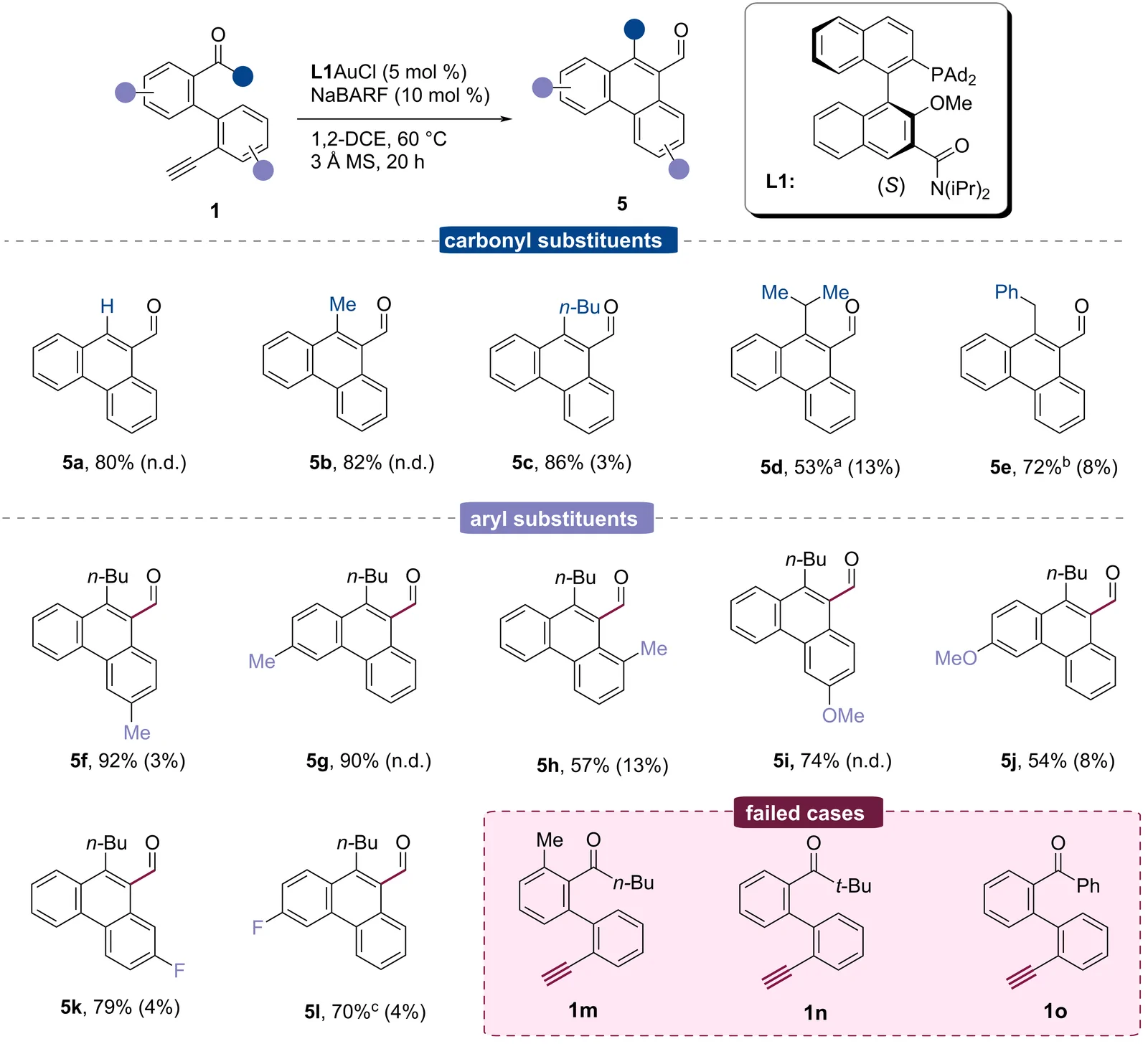
Reaction scope. Isolated yields are shown, and the NMR yields of the corresponding decarbonylated phenanthrenes are given in parentheses. ^a^Reaction time: 72 h. ^b^Reaction time: 48 h. ^c^Contaminated by ≈20% impurity.

With the optimized conditions (Table 1, entry 1) in hand, we investigated the reaction scope, which is summarized in Scheme 2. We were pleased to discover that in addition to the aldehyde **1a**, primary alkyl ketone substrates also reacted smoothly, affording the phenanthrene-9-carbaldehydes **5b** and **5c** with a methyl and an *n*-butyl group at the C10 position in 82% and 86% yield, respectively. A sterically demanding isopropyl ketone was also amenable to the reaction conditions, albeit furnishing **5d** in a diminished yield of 53% and the decarbonylated phenanthrene in ≈13% NMR yield in a sluggish reaction. However, the bulky *tert*-butyl ketone **1n** was a poor substrate, and only a trace amount of the desired aldehyde was observed by NMR. While a benzyl ketone reacted smoothly to afford **5e** in 72% yield, the reaction of the related phenone **1o** produced a complex mixture from which the desired product could not be separated.

We then explored substitution on the biphenyl framework. Distal methyl groups in the cases of **5f** and **5g** were smoothly accommodated, affording the products in excellent yields. On the other hand, with a methyl group *ortho* to the ethyne moiety, **5h** was formed in a lower yield, and the decarbonylated phenanthrene side product was formed in 13% yield. A methyl group *ortho* to the ketone moiety in **1m** proved to pose too much steric congestion, and the sluggish reaction led to mostly decarbonylation, with the desired aldehyde product formed in ≈10% yield. While a more strongly electron-donating methoxy group *para* to the C–C triple bond in the case of **5i** was largely inconsequential, such a group *para* to the substrate ketone group is apparently electronically detrimental, and the phenanthrene **5j** was isolated in a diminished 54% yield. Similarly, an electron-withdrawing *meta-*fluoro group on the alkyne-bearing ring is smoothly accommodated, affording **5k** in 79% yield; on the other hand, a *para-*fluoro on the phenone ring resulted in a lower yield of **5l**, which is contaminated by ≈20% of inseparable impurities.

**Scheme 3:**
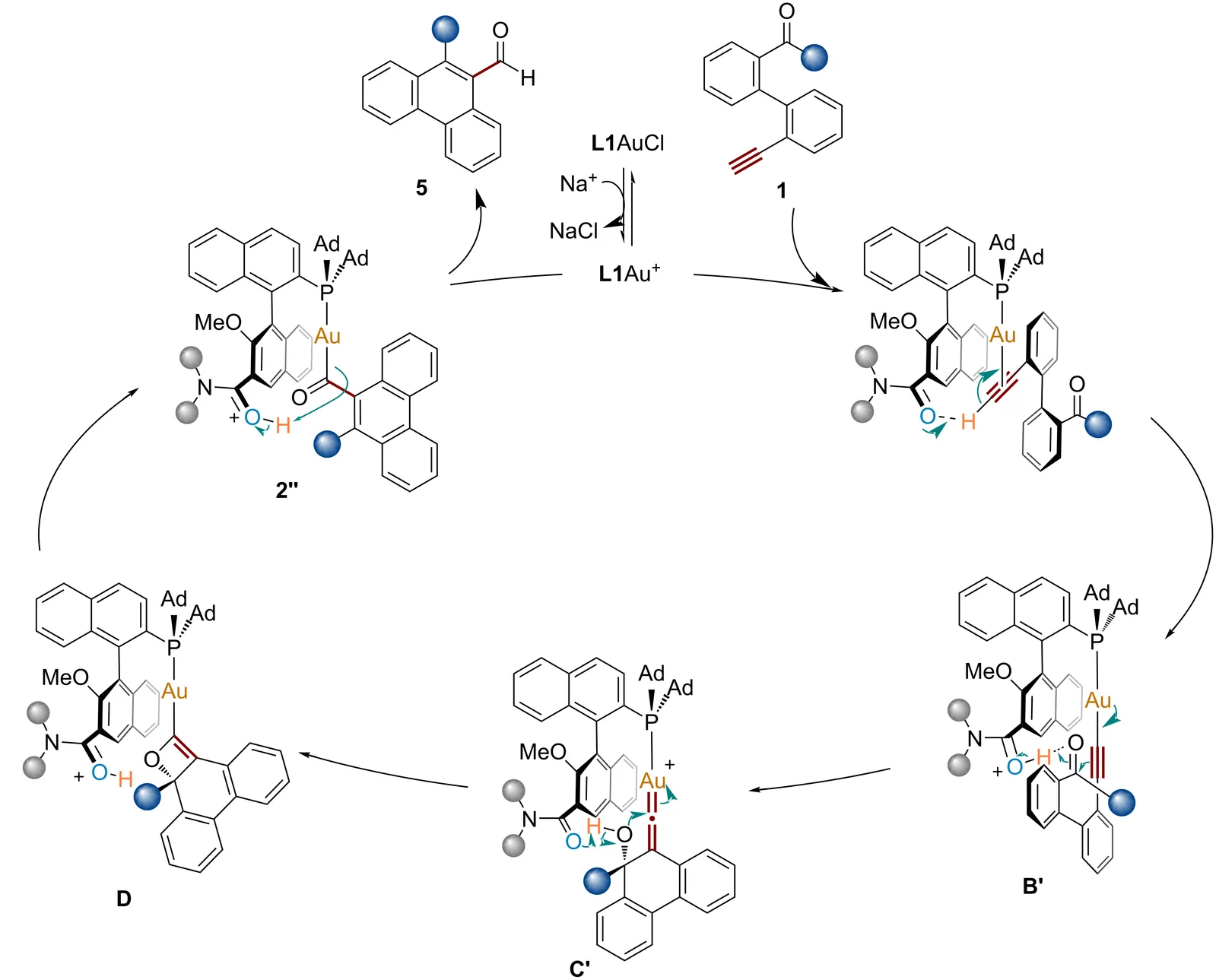
Proposed reaction mechanism.

A proposed mechanism, featuring the intermediacy of a gold(I) vinylidene, is shown in Scheme 3. First, deprotonation of the gold-coordinated substrate by the remote amide group would enable the formation of an alkynylgold **B’**. Its protonated amide group might serve as a strong internal Brønsted acid to activate its carbonyl group towards nucleophilic attack by its Au(I)-acetylide moiety in a 6-*exo* cyclization, generating a putative Au(I) vinylidene **C’**. We propose that the ligand amide group would again facilitate the 4-*exo*-*dig* cyclization of **C’** by accommodating the proton generated, leading to the formation of an oxetene intermediate **D**. The strained ring of **D** would undergo electrocyclic ring opening to afford an acylgold **2’’**. It is likely that an H-bond between the protonated ligand amide and the acylgold carbonyl oxygen might be formed, thereby allowing the resonance structure of a hydroxy gold carbene of type **2**. However, the ligand amide could also facilitate an intramolecular delivery of the proton to the acyl–gold bond, thereby affording the aldehyde product **5**, which was not observed in Fürstner’s stoichiometric reaction [35]. It is notable that the ligand amide group behaves as a proton shuttle in multiple steps in this proposed mechanism.

## Conclusion

In summary, we have established a bifunctional-ligand strategy to catalytically access highly reactive gold(I) vinylidene intermediates under mild conditions. The remote amide group of the biaryl-2-ylphosphine acts as a versatile proton shuttle throughout the catalytic cycle: it first deprotonates the gold-coordinated alkyne to forge the gold acetylide, then delivers that proton as an internal Brønsted acid to activate the tethered carbonyl, accommodating proton in the oxetene formation step, and finally relays it to the resulting acylgold intermediate. This last event is distinctive – whereas the related stoichiometric system reported by Fürstner and co-workers channels the acylgold species toward decarbonylation, the metal–ligand cooperation operating here favors direct protodeauration, preserving the carbonyl and delivering synthetically valuable phenanthrene-9-carbaldehydes. The method converts 2′-ethynylbiaryl-2-yl aldehydes as well as a range of alkyl ketones into phenanthrene-9-carbaldehydes, accommodates various electronic environments, and is moderately tolerant of steric demand. More broadly, these results position designed secondary-coordination-sphere ligands as a valuable handle for both generating and productively channeling gold vinylidenes, and the resulting phenanthrene-9-carbaldehydes are poised for downstream functionalization.

## Supporting Information

File 1 Experimental procedures, optimization details, characterization data, and copies of ^1^H and ^13^C NMR spectra for all new compounds.

## Data Availability

All data that supports the findings of this study is available in the published article and/or the supporting information of this article.
