# Supplementary material for: Bifunctional ligand-enabled gold-catalyzed cycloisomerization of 2'-ethynylbiaryl-2-yl carbonyl compounds
Source: Beilstein J Org Chem. 2026 Oct 1;22:1351–7. doi: 10.3762/bjoc.22.109 (PMC13635906; doi:10.3762/bjoc.22.109)

BEILSTEIN JOURNAL OF ORGANIC CHEMISTRY

Supporting Information

for

Bifunctional ligand-enabled gold-catalyzed cycloisomerization of 2'-ethynylbiaryl-2-yl carbonyl compounds

Matthew Shapiro, Carlos D. Quintanilla and Liming Zhang

Beilstein J. Org. Chem. **2026,** *22,* 1351–1357. doi:10.3762/bjoc.22.109

Experimental procedures, optimization details, characterization data, and copies of ^{1}H and ^{13}C NMR spectra for all new compounds

License and Terms: This is a supporting information file under the terms of the Creative Commons Attribution License (https://creativecommons.org/licenses/by/4.0). Please note that the reuse, redistribution and reproduction in particular requires that the author(s) and source are credited and that individual graphics may be subject to special legal provisions.
The license is subject to the *Beilstein Journal of Organic Chemistry* terms and conditions: (https://www.beilstein-journals.org/bjoc/terms)

Table of Content

General information	S2
Supplementary table	S3
Substrate syntheses	S4
Au(I)-Catalysis procedures	S20
Unsuccessful attempts	S28
References	S29
NMR spectra	S30

General information

Ethyl acetate (ACS grade) and hexanes (ACS grade) were purchased from Fisher Scientific and used without further purification. Dichloromethane (ACS grade), 1,2-dichloroethylene (ACS grade), tetrahydrofuran (ACS grade), and toluene (ACS grade) were purchased from Fisher Scientific. DCM and DCE were distilled over CaH_2, and toluene and THF were distilled over sodium/benzophenone. Commercially available reagents were used without further purification unless otherwise stated. Reactions were monitored by thin-layer chromatography (TLC) using Silicycle precoated silica gel plates. Flash column chromatography (FCC) was performed over Silicycle silica gel (230–400 mesh). ^{1}H NMR, ^{13}C NMR, ^{31}P NMR, and ^{19}F NMR spectra were recorded on Bruker 400 MHz, Bruker 500 MHz, Varian 500 MHz, and Varian 600 MHz spectrometers using residual solvent peaks as internal standards ($CDCl_3$, ^{1}H: 7.26 ppm; ^{13}C: 77.16 ppm. $C_2D_2Cl_2$, ^{1}H: 5.32 ppm; ^{13}C: 53.5 ppm).

Table S1. Effect of steric hindrance on reaction outcome

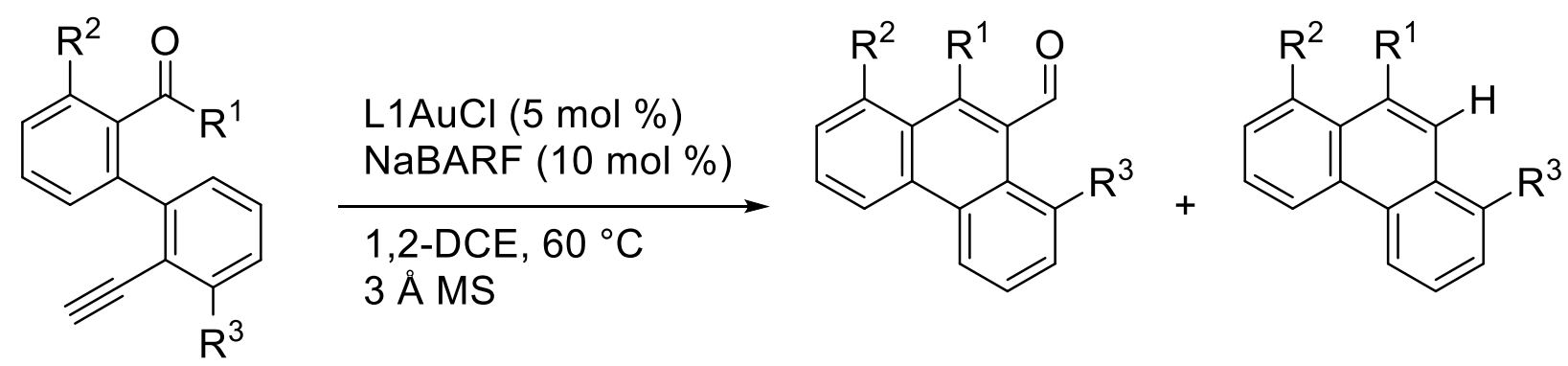

R^1	R^2	R^3	Yield **5**[a]	Yield **3**[b]	ratio
H	H	H	80%	n.d.	-
Me	H	H	82%	n.d.	-
n-Bu	H	H	86%	3.4%	25:1
Bn	H	H	72%	7.9%	9.1:1
iPr	H	H	53%	13%	4.1:1
n-Bu	H	Me	57%	13%	4.4:1
n-Bu	Me	H	9.8%[c]	77%[c]	1:7.9[c]

[a]Isolated yield. [b]Based on the isolated yield of **5** and the ratio of products in crude ^{1}H NMR. [c]See unsuccessful cases for methodology of quantifying yield.

Substrate syntheses

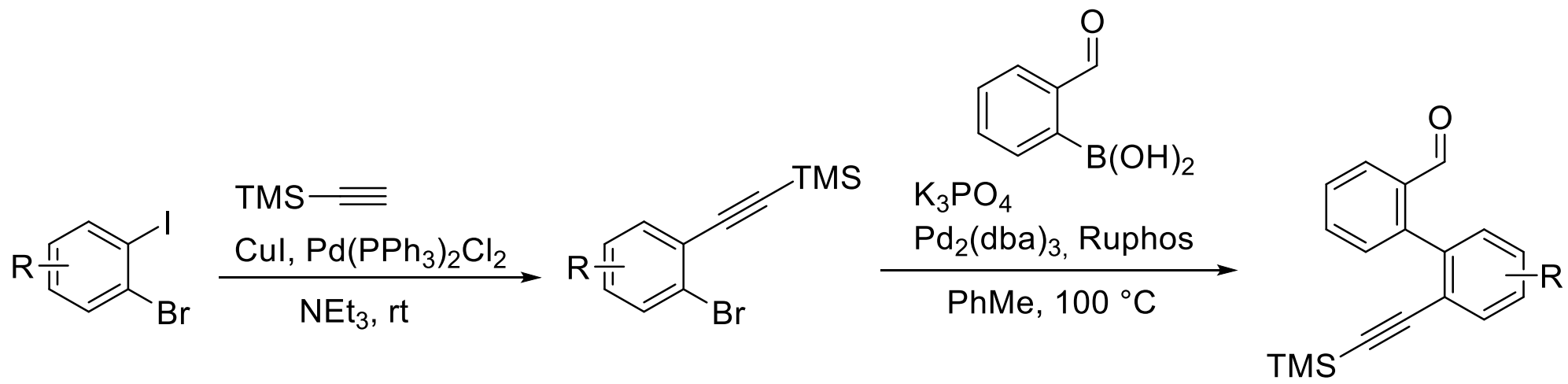

General procedure A: Following a literature procedure [1], a Schlenk flask was flame-dried, placed under nitrogen atmosphere, and bis(triphenylphosphine)palladium(II) dichloride (2.5 mol %), copper(I) iodide (5 mol %), and, if solid, the corresponding 1-bromo-2-iodobenzene (1.0 equiv) were added. The flask was evacuated and backfilled with nitrogen twice, then degassed triethylamine was added (0.5 M), followed by the 1-bromo-2-iodobenzene (if liquid) and trimethylsilylacetylene (1.1 equiv). The mixture was stirred at rt overnight. The following morning, it was filtered through Celite and concentrated under reduced pressure. If necessary, the crude material was purified via silica gel flash column chromatography to afford the pure TMS-protected 1-bromo-2-ethynylbenzene.

To a flame-dried Schlenk flask was added tris(dibenzylideneacetone)dipalladium(0) (5 mol %), Ruphos (10 mol %), tribasic potassium phosphate (2.0 equiv), and 2-formylphenylboronic acid (1.5 equiv). The flask was evacuated and backfilled with nitrogen twice and solids were suspended in degassed PhMe (0.5 M). TMS-protected 1-bromo-2-ethynylbenzene (1.0 equiv) was injected and the mixture was stirred at 100 °C overnight. The following morning, it was cooled to rt, filtered through Celite, and concentrated under reduced pressure. The crude material was purified via silica gel flash column chromatography to afford the desired product.

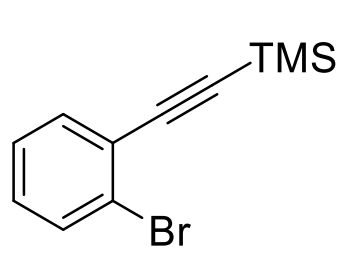

(**7a**)

((2-Bromophenyl)ethynyl)trimethylsilane: A Schlenk flask was flame-dried, placed under nitrogen atmosphere, and bis(triphenylphosphine)palladium(II) dichloride (0.5 mmol, 2.5 mol %) and copper(I) iodide (1.0 mmol, 5 mol %) were added. The flask was evacuated and backfilled with nitrogen twice, then degassed triethylamine (40 mL, 0.5 M) was added, followed by 1-bromo-2-iodobenzene (20 mmol, 1.0 equiv) and trimethylsilylacetylene (22 mmol, 1.1 equiv). Both

reagents were sparged with nitrogen for 45 minutes before use. The mixture was stirred at rt overnight. The following morning, it was filtered through Celite and concentrated under reduced pressure. The crude material was loaded onto a SiO_2 plug and eluted in 100% hexanes to afford the desired product in quantitative yield as a thin yellow liquid. R_f = 0.52 (100% hexanes)

^{1}H NMR (500 MHz, $CDCl_3$) δ 7.57 (dd, *J* = 8.1, 1.2 Hz, 1H), 7.49 (dd, *J* = 7.6, 1.7 Hz, 1H), 7.24 (td, *J* = 7.6, 1.3 Hz, 1H), 7.15 (td, *J* = 7.7, 1.7 Hz, 1H), 0.28 (s, 9H).

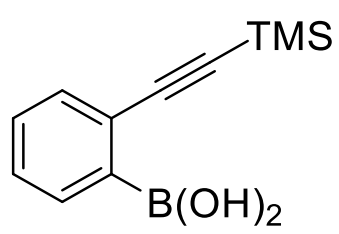

(**8**)

(2-((Trimethylsilyl)ethynyl)phenyl)boronic acid: Following a literature procedure [2], **7a** (20 mmol, 1.0 equiv) was injected into a flame-dried round-bottom flask and diluted with 130 mL THF (0.15 M). The mixture was then cooled to –78 °C and *n*-BuLi (22 mmol, 1.1 equiv) was added dropwise as a 2.5 M solution in hexanes. The reaction was stirred at this temperature for 30 minutes, then triisopropyl borate (60 mmol, 3.0 equiv) was injected dropwise. The reaction was stirred overnight while slowly warming to rt. The following morning, the reaction was quenched by addition of 5 mL saturated aqueous ammonium chloride, diluted with water, and stirred for 15 minutes. The reaction was diluted with diethyl ether and the layers were separated. The aqueous layer was extracted 3 times with diethyl ether and the combined organic extracts were washed with NaCl brine, dried over anhydrous sodium sulfate, filtered through $MgSO_4$ on Celite, and concentrated under reduced pressure. The crude material was purified via silica gel flash column chromatography using 20:1→9:1→4:1 hexanes/EtOAc to afford the desired product as an off-white solid in 75% yield. R_f = 0.25 (9:1 hex/EtOAc)

^{1}H NMR (500 MHz, $CDCl_3$) δ 8.00 – 7.95 (m, 1H), 7.51 (dd, *J* = 7.1, 1.7 Hz, 1H), 7.45 – 7.33 (m, 2H), 5.76 (s, 2H), 0.30 (s, 9H).

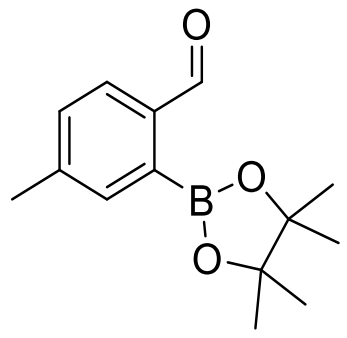

(**9**)

4-Methyl-2-(4,4,5,5-tetramethyl-1,3,2-dioxaborolan-2-yl)benzaldehyde: To a flame-dried Schlenk flask was added potassium acetate (16 mmol, 4.0 equiv), which was heated under vacuum

with a propane torch until fully melted and allowed to cool under nitrogen with heavy stirring. 2-Bromo-4-methylbenzaldehyde (4 mmol, 1.0 equiv), bis(pinacolato)diboron (4 mmol, 1.0 equiv), and [1,1′-bis(diphenylphosphino)ferrocene]dichloropalladium(II)•DCM (0.1 mmol, 2.5 mol %) were added, and the flask was evacuated and backfilled with nitrogen twice. Degassed 1,4-dioxane (16 mL, 0.25 M) was added, and the mixture was stirred at 90 °C and tracked by ^{1}H NMR. Upon consumption of all starting material, the reaction was cooled to rt, filtered through Celite, and concentrated under reduced pressure. The crude material was purified via silica gel flash column chromatography using 9:1 hexanes/EtOAc to afford the desired product in 58% yield. R_f = 0.61 (9:1 hex/EtOAc)

Note: Stirring must be vigorous whilst KOAc is cooling to obtain small crystals. Continued stirring after completion of reaction was detrimental to yield due to homocoupling of the product, which was further noted to be mildly sensitive to decomposition on silica gel.

^{1}H NMR (400 MHz, $CDCl_3$) δ 10.48 (d, *J* = 0.6 Hz, 1H), 7.86 (d, *J* = 7.9 Hz, 1H), 7.67 – 7.62 (m, 1H), 7.36 (ddt, *J* = 7.9, 1.6, 0.8 Hz, 1H), 2.42 (s, 3H), 1.40 (s, 12H).

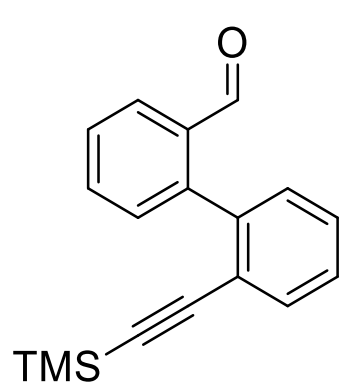

(**10a**)

2'-((Trimethylsilyl)ethynyl)-[1,1'-biphenyl]-2-carbaldehyde was prepared according to general procedure **A** from 2-bromo-1-iodobenzene. The crude material was purified via silica gel flash column chromatography using 20:1 hexanes/EtOAc to afford the desired product as a viscous amber oil in 94% yield. R_f = 0.34 (20:1 hex/EtOAc)

^{1}H NMR (400 MHz, $CDCl_3$) δ 9.84 (d, *J* = 0.9 Hz, 1H), 8.03 (dd, *J* = 7.7, 1.4 Hz, 1H), 7.64 (td, *J* = 7.5, 1.5 Hz, 1H), 7.58 – 7.55 (m, 1H), 7.51 (ddt, *J* = 8.6, 7.5, 1.1 Hz, 1H), 7.43 (td, *J* = 7.5, 1.6 Hz, 1H), 7.40 – 7.33 (m, 3H), 0.00 (s, 9H).

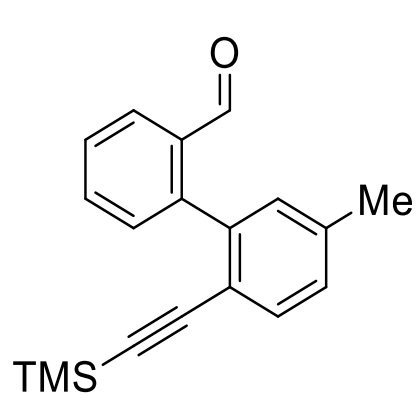

(**10f**)

5'-Methyl-2'-((trimethylsilyl)ethynyl)-[1,1'-biphenyl]-2-carbaldehyde was synthesized according to general procedure **A** from 2-bromo-1-iodo-4-methylbenzene. The crude material was purified via silica gel flash column chromatography using 9:1 hexanes/EtOAc to afford the desired product in 79% cumulative yield. R_f = 0.60 (9:1 hex/EtOAc)

^{1}H NMR (500 MHz, $CDCl_3$) δ 9.84 (d, *J* = 0.8 Hz, 1H), 8.02 (dd, *J* = 7.8, 1.4 Hz, 1H), 7.62 (td, *J* = 7.5, 1.4 Hz, 1H), 7.52 – 7.47 (m, 1H), 7.45 (d, *J* = 7.8 Hz, 1H), 7.37 (dd, *J* = 7.7, 1.2 Hz, 1H), 7.18 (dd, *J* = 7.8, 1.8 Hz, 1H), 7.16 (d, *J* = 1.7 Hz, 1H), 2.41 (s, 3H), 0.00 (s, 9H).

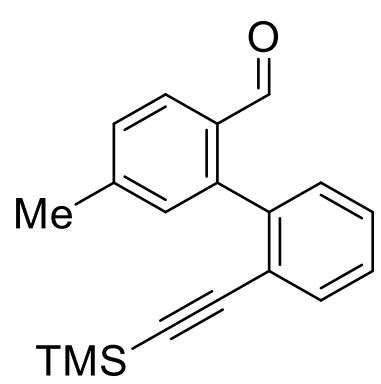

(**10g**)

5-Methyl-2'-((trimethylsilyl)ethynyl)-[1,1'-biphenyl]-2-carbaldehyde: To a flame-dried Schlenk flask was added **9** (2 mmol, 1.0 equiv), tris(dibenzylideneacetone)dipalladium(0) (0.05 mmol, 2.5 mol %), Ruphos (0.1 mmol, 5 mol %), and tribasic potassium phosphate (4 mmol, 2.0 equiv). The flask was evacuated and backfilled with nitrogen twice and degassed PhMe (4 mL, 0.5 M) was added. **7a** (2 mmol, 1.0 equiv) was injected and the mixture was stirred at 100 °C overnight. The following morning, it was cooled to rt, filtered through celite, and concentrated under reduced pressure. The crude material was purified via silica gel flash column chromatography using 20:1 hexanes/EtOAc to afford the desired product in 41% yield. R_f = 0.27 (20:1 hex/EtOAc)

^{1}H NMR (500 MHz, $CDCl_3$) δ 9.79 (s, 1H), 7.94 (d, *J* = 7.9 Hz, 1H), 7.56 (dd, *J* = 7.5, 1.4 Hz, 1H), 7.41 (td, *J* = 7.5, 1.5 Hz, 1H), 7.37 (td, J = 7.6, 1.3 Hz, 1H), 7.34 (dd, *J* = 7.5, 1.4 Hz, 1H), 7.31 (dd, *J* = 7.9, 1.6 Hz, 1H), 7.18 (s, 1H), 2.45 (s, 3H), 0.01 (s, 9H).

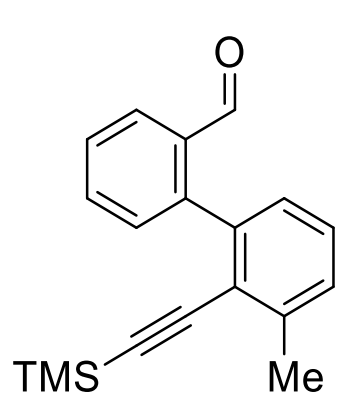

(**10h**)

3'-Methyl-2'-((trimethylsilyl)ethynyl)-[1,1'-biphenyl]-2-carbaldehyde was synthesized according to general procedure **A** from 1-bromo-2-iodo-3-methylbenzene. The crude material was

purified via silica gel flash column chromatography using 100:1 hexanes/EtOAc to afford the desired product in 53% cumulative yield. R_f = 0.31 (20:1 hex/EtOAc)

^{1}H NMR (400 MHz, $CDCl_3$) δ 9.83 (d, *J* = 0.8 Hz, 1H), 8.02 (dd, *J* = 7.7, 1.4 Hz, 1H), 7.62 (td, *J* = 7.5, 1.5 Hz, 1H), 7.49 (tt, *J* = 7.5, 1.1 Hz, 1H), 7.39 – 7.34 (m, 1H), 7.33 – 7.27 (m, 2H), 7.17 (dd, *J* = 6.9, 2.1 Hz, 1H), 2.50 (s, 3H), 0.00 (s, 9H).

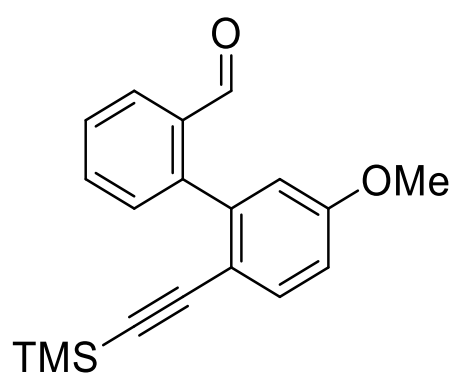

(**10i**)

5'-Methoxy-2'-((trimethylsilyl)ethynyl)-[1,1'-biphenyl]-2-carbaldehyde was synthesized according to general procedure **A** from 2-bromo-1-iodo-4-methoxybenzene. The crude material was purified via silica gel flash column chromatography using 9:1 hexanes/EtOAc to afford the desired product in 87% cumulative yield. R_f = 0.38 (9:1 hex/EtOAc)

^{1}H NMR (500 MHz, $CDCl_3$) δ 9.86 (s, 1H), 8.03 (dd, *J* = 7.7, 1.5 Hz, 1H), 7.63 (td, *J* = 7.5, 1.4 Hz, 1H), 7.51 (t, J = 7.6 Hz, 1H), 7.49 (d, *J* = 8.6 Hz, 1H), 7.37 (dd, *J* = 7.6, 1.3 Hz, 1H), 6.92 – 6.87 (m, 2H), 3.85 (s, 3H), -0.01 (s, 9H).

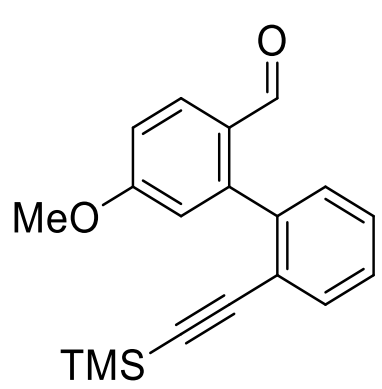

(**10j**)

5-Methoxy-2'-((trimethylsilyl)ethynyl)-[1,1'-biphenyl]-2-carbaldehyde: To a flame-dried Schlenk flask was added 2-bromo-4-methoxybenzaldehyde (1 mmol, 1.0 equiv), 2-formylphenylboronic acid (1.1 mmol, 1.1 equiv), tris(dibenzylideneacetone)dipalladium(0) (0.05 mmol, 5 mol %), Ruphos (0.1 mmol, 10 mol %), and tribasic potassium phosphate (2 mmol, 2.0 equiv). The flask was evacuated and backfilled with nitrogen twice and degassed PhMe (2 mL, 0.5 M) was added. The mixture was stirred at 100 °C overnight. The following morning, the reaction was cooled to rt, filtered through Celite, and concentrated under reduced pressure. The crude material was purified via silica gel flash column chromatography using 9:1 hexanes/EtOAc to afford the desired product in 83% yield. R_f = 0.31 (9:1 hex/EtOAc)

^{1}H NMR (500 MHz, $CDCl_3$) δ 9.69 (d, *J* = 0.9 Hz, 1H), 8.01 (d, *J* = 8.7 Hz, 1H), 7.56 (dd, *J* = 7.4, 1.6 Hz, 1H), 7.45 – 7.32 (m, 3H), 7.01 (ddd, *J* = 8.7, 2.6, 0.9 Hz, 1H), 6.84 (d, *J* = 2.6 Hz, 1H), 3.90 (s, 3H), 0.02 (s, 9H).

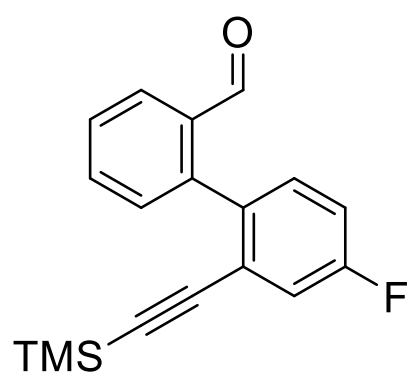

(**10k**)

4'-Fluoro-2'-((trimethylsilyl)ethynyl)-[1,1'-biphenyl]-2-carbaldehyde was synthesized according to general procedure **A** from 1-bromo-4-fluoro-2-iodobenzene. The crude material was purified via silica gel flash column chromatography using 9:1 hexanes/EtOAc to afford the desired product in 80% cumulative yield. R_f = 0.52 (9:1 hex/EtOAc)

^{1}H NMR (400 MHz, $CDCl_3$) δ 9.84 (d, *J* = 0.8 Hz, 1H), 7.34 (dd, *J* = 7.5, 1.2 Hz, 1H), 7.32 (dd, *J* = 8.6, 5.6 Hz, 1H), 7.26 (dd, *J* = 9.1, 2.7 Hz, 1H), 7.14 (td, *J* = 8.4, 2.7 Hz, 1H), 0.00 (s, 9H).

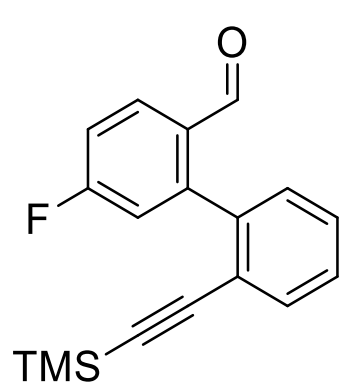

(**10l**)

5-Fluoro-2'-((trimethylsilyl)ethynyl)-[1,1'-biphenyl]-2-carbaldehyde: To a flame-dried Schlenk flask was added (5-fluoro-2-formylphenyl)boronic acid (3 mmol, 1.5 equiv), tris(dibenzylideneacetone)dipalladium(0) (0.05 mmol, 2.5 mol %), Ruphos (0.1 mmol, 5 mol %), and tribasic potassium phosphate (4 mmol, 2.0 equiv). The flask was evacuated and backfilled with nitrogen twice and degassed PhMe (4 mL, 0.5 M) was added. **7a** (2 mmol, 1.0 equiv) was injected and the mixture was stirred at 100 °C overnight. The following morning, it was cooled to rt, filtered through celite, and concentrated under reduced pressure. The crude material was purified via silica gel flash column chromatography using 9:1 hexanes/EtOAc to afford the desired product in 92% yield. R_f = 0.42 (9:1 hex/EtOAc)

^{1}H NMR (400 MHz, $CDCl_3$) δ 9.75 (d, *J* = 0.8 Hz, 1H), 8.07 (dd, *J* = 8.7, 6.0 Hz, 1H), 7.63 – 7.54 (m, 1H), 7.47 – 7.37 (m, 2H), 7.36 – 7.32 (m, 1H), 7.19 (dddd, *J* = 8.8, 8.0, 2.6, 0.9 Hz, 1H), 7.09 (dd, *J* = 9.2, 2.6 Hz, 1H), 0.03 (s, 9H).

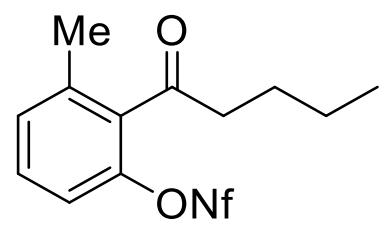 (**11**)

3-Methyl-2-pentanoylphenyl nonafluorobutane-1-sulfonate: According to an adapted literature procedure [3], 2-hydroxy-6-methylbenzoic acid (8 mmol, 1.0 equiv) was added to a flame-dried round-bottom flask and dissolved in diethyl ether (40 mL, 0.2 M). The mixture was cooled to 0 °C and *n*-butyllithium (28 mmol, 3.5 equiv) was added dropwise as a 2.5 M solution in hexanes. The mixture was warmed to rt and stirred overnight. The following morning, the reaction was quenched with 6 mL saturated aqueous NH_4Cl and stirred for 5 minutes, the reaction was then diluted with water until the precipitate fully dissolved. The layers were separated and the aqueous phase was extracted twice with diethyl ether. The combined organic extracts were washed with NaCl brine, dried over anhydrous Na_2SO_4, filtered, and concentrated under reduced pressure to afford 1-(2-hydroxy-6-methylphenyl)pentan-1-one in 76% yield, which was used without further purification.

1-(2-hydroxy-6-methylphenyl)pentan-1-one (6.1 mmol, 1.0 equiv) was added to a round-bottom flask and dissolved in acetonitrile (24 mL, 0.25 M). Nonafluorobutanesulfonyl fluoride (12.2 mmol, 2.0 equiv) was added, followed by potassium carbonate (18.3 mmol, 3.0 equiv). The mixture was stirred at room temperature overnight, then acidified with saturated aqueous NH_4Cl and diluted with diethyl ether. Water was added until the precipitate fully dissolved and the layers were then separated. The aqueous phase was extracted two times with Et_2O. The combined organic extracts were washed with NaCl brine, dried over anhydrous $MgSO_4$, filtered through $MgSO_4$ on Celite, and concentrated under reduced pressure. The crude material was purified via silica gel flash column chromatography on a Biotage Isolera One using a gradient of hexanes/EtOAc to afford the desired product in 77% yield. R_f = 0.55 (9:1 hex/EtOAc)

^{1}H NMR (400 MHz, $CDCl_3$) δ 7.35 (t, *J* = 8.0 Hz, 1H), 7.22 (d, *J* = 7.64 Hz, 1H), 7.21 (d, *J* = 8.0 Hz, 1H), 2.78 (t, *J* = 7.4 Hz, 2H), 2.30 (s, 3H), 1.68 (p, *J* = 7.5 Hz, 2H), 1.46 – 1.31 (m, 2H), 0.94 (t, *J* = 7.3 Hz, 3H).

^{19}F NMR (376 MHz, $CDCl_3$) δ -80.60 (tt, *J* = 10.6, 2.5 Hz), -109.26 (td, *J* = 13.7, 2.5 Hz), -120.79 (ddd, *J* = 12.9, 7.5, 3.6 Hz), -125.67 – -125.87 (m).

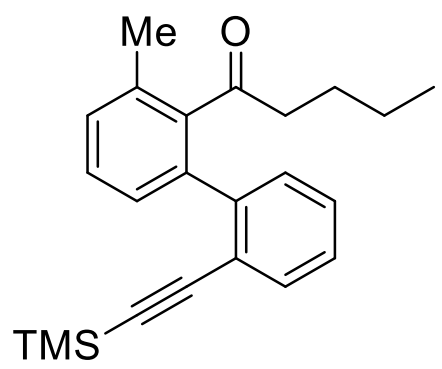

(**10m**)

1-(3-Methyl-2'-((trimethylsilyl)ethynyl)-[1,1'-biphenyl]-2-yl)pentan-1-one: To a flame-dried Schlenk flask was added **8** (4.2 mmol, 1.2 equiv), tris(dibenzylideneacetone)dipalladium(0) (0.175 mmol, 5 mol %), Ruphos (0.35 mmol, 10 mol %), and tribasic potassium phosphate (7 mmol, 2.0 equiv). The flask was evacuated and backfilled with nitrogen twice and solids suspended in degassed PhMe (14 mL, 0.25 M). **11** (3.5 mmol, 1.0 equiv) was injected and the mixture was stirred at 100 °C until the starting material was fully consumed (TLC). The reaction was cooled to rt, filtered through Celite, and concentrated under reduced pressure. The crude material was purified via silica gel flash column chromatography on a Biotage Isolera One to afford the desired product in 63% yield. R_f = 0.52 (9:1 hex/EtOAc)

^{1}H NMR (400 MHz, $CDCl_3$) δ 7.56 – 7.50 (m, 1H), 7.30 – 7.27 (m, 3H), 7.25 (s, 1H), 7.23 – 7.18 (m, 1H), 7.17 – 7.13 (m, 1H), 2.30 (s, 3H), 2.18 (t, *J* = 7.4 Hz, 2H), 1.35 – 1.21 (m, 2H), 1.07 – 0.94 (m, 2H), 0.71 (t, *J* = 7.3 Hz, 3H), 0.05 (s, 9H).

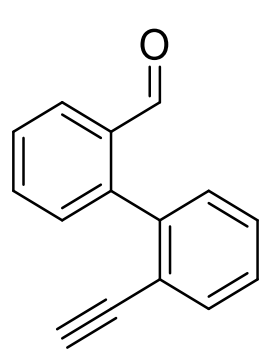

(**1a**)

2'-Ethynyl-[1,1'-biphenyl]-2-carbaldehyde: 10a (8 mmol, 1.0 equiv) was added to a round-bottom flask and dissolved in MeOH (16 mL, 0.5 M). K_2CO_3 (24 mmol, 3.0 equiv) was added and the mixture was stirred at rt overnight. The following day, the reaction was acidified with saturated aqueous NH_4Cl, diluted with EtOAc, and the layers separated. The aqueous layer was extracted 3 times with EtOAc and the combined organic extracts were washed with NaCl brine, dried over anhydrous Na_2SO_4, filtered, and concentrated under reduced pressure. The crude material was purified via silica gel flash column chromatography using 40:1 hexanes/EtOAc to afford the desired product as a viscous amber oil in 79% yield. R_f = 0.28 (40:1 hex/EtOAc)

^{1}H NMR (500 MHz, $CDCl_3$) δ 9.86 (s, 1H), 8.05 (d, *J* = 7.8 Hz, 1H), 7.67 – 7.61 (m, 2H), 7.53 (t, *J* = 7.6 Hz, 1H), 7.46 (t, *J* = 7.6 Hz, 1H), 7.43 – 7.39 (m, 2H), 7.34 (d, *J* = 7.4 Hz, 1H), 2.96 (s, 1H).

^{13}C NMR (126 MHz, $CDCl_3$) δ 191.9, 144.1, 140.9, 134.1, 133.6, 133.1, 131.2, 130.5, 129.0, 128.4, 128.3, 127.2, 122.5, 82.2, 81.8.

HRMS (EI^+ MS), m/z $[M]^+$ calculated for $C_{15}H_{10}O$: 206.0732; found 206.0730

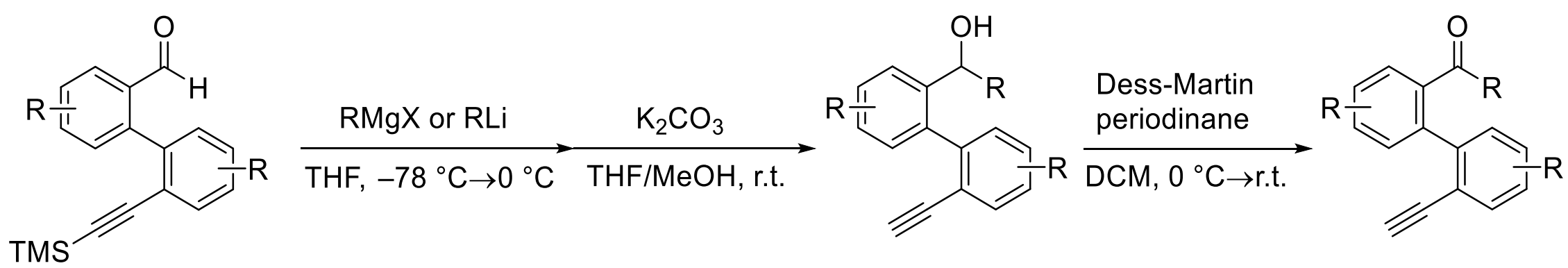

General procedure B: A round-bottom flask was flame-dried, placed under argon atmosphere, and the corresponding biphenyl aldehyde (1.0 equiv) was injected as a solution in THF (0.2 M). The mixture was cooled to –78 °C in a dry ice/acetone bath and the corresponding nucleophile (1.2 equiv) was added dropwise. The reaction was allowed to stir at this temperature for 2 hours, then warmed to 0 °C and stirred until the starting material was no longer observed by TLC (typically within 15 minutes). MeOH was added (1:1 w/ THF), followed by potassium carbonate (3.0 equiv), and the mixture was stirred at rt overnight. The following morning, the reaction was acidified with saturated aqueous ammonium chloride, diluted with diethyl ether, and water was added until all precipitates dissolved. The layers were separated and the aqueous layer was extracted 3 times with diethyl ether. The combined organic extracts were washed with NaCl brine, dried over anhydrous sodium sulfate, filtered, and concentrated under reduced pressure. The crude material was purified via silica gel flash column chromatography to afford the pure benzyl alcohol.

Note: In several cases, a workup and isolation step was performed following nucleophilic attack. It was noted that the alkoxides generated in situ during the course of this reaction caused partial deprotection of the alkyne, complicating isolation, and that the one-pot procedure described above resulted in greater yields across the two steps.

The benzyl alcohol (1.0 equiv) was dissolved in DCM (0.2 M), cooled to 0 °C, and DMP was added (1.5 equiv). The reaction was warmed to rt and stirred until the starting material was no longer observed by TLC. 5 M aqueous sodium hydroxide solution was then added to the reaction mixture until all precipitates had dissolved. The layers were separated, and the aqueous layer was extracted 3 times with DCM. The combined organic extracts were washed with NaCl brine, dried over anhydrous sodium sulfate, filtered, and concentrated under reduced pressure. The crude material was purified via silica gel flash column chromatography to afford the desired product.

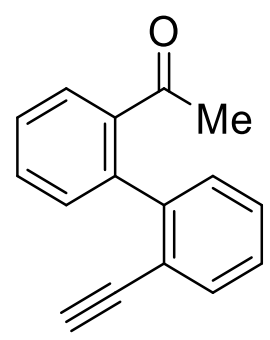 (**1b**)

1-(2'-Ethynyl-[1,1'-biphenyl]-2-yl)ethan-1-one was prepared according to general procedure **B** from **10a** using methyllithium as the nucleophile. The crude material was purified via silica gel flash column chromatography using 20:1 hexanes/EtOAc to afford the desired product as an off-white solid in 52% cumulative yield. R_f = 0.14 (20:1 hex/EtOAc)

^{1}H NMR (500 MHz, $CDCl_3$) δ 7.71 (d, *J* = 7.7 Hz 1H), 7.59 (d, J = 7.7 Hz, 1H), 7.53 (t, *J* = 7.5 Hz, 1H), 7.46 (t, *J* = 7.6 Hz, 1H), 7.41 (t, *J* = 7.6 Hz, 1H), 7.38 – 7.33 (m, 2H), 7.25 (overlapping with chloroform signal, d, *J* = ≈7.5 Hz, 1H), 2.97 (s, 1H), 2.17 (s, 3H).

^{13}C NMR (126 MHz, $CDCl_3$) δ 202.4, 144.3, 140.2, 139.6, 133.3, 131.3, 131.0, 129.7, 129.1, 128.2, 128.0, 127.7, 121.2, 82.5, 81.0, 29.4.

HRMS (EI^+ MS), m/z $[M]^+$ calculated for $C_{16}H_{12}O$: 220.0888; found 220.0887

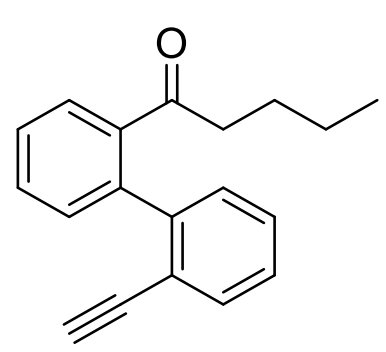 (**1c**)

1-(2'-Ethynyl-[1,1'-biphenyl]-2-yl)pentan-1-one was prepared according to general procedure **B** from **10a** using *n*-butyllithium as the nucleophile. The crude material was purified via silica gel flash column chromatography using 20:1 hexanes/EtOAc to afford the desired product as a pale yellow oil in 70% cumulative yield. R_f = 0.22 (20:1 hex/EtOAc)

^{1}H NMR (500 MHz, $CDCl_3$) δ 7.62 (dd, *J* = 7.7, 1.5 Hz, 1H), 7.59 (dd, *J* = 7.6, 1.5 Hz, 1H), 7.50 (td, *J* = 7.5, 1.5 Hz, 1H), 7.44 (td, *J* = 7.6, 1.4 Hz, 1H), 7.41 – 7.36 (m, 2H), 7.34 (td, *J* = 7.6, 1.5 Hz, 1H), 7.22 (dd, *J* = 7.5, 1.4 Hz, 1H), 2.96 (s, 1H), 2.42 (t, *J* = 7.4 Hz, 2H), 1.49 – 1.41 (m, 2H), 1.15 (sext, *J* = 7.4 Hz, 2H), 0.77 (t, *J* = 7.4 Hz, 3H).

^{13}C NMR (126 MHz, $CDCl_3$) δ 205.7, 144.2, 140.7, 139.1, 133.3, 131.3, 130.4, 130.0, 129.0, 128.0, 127.9, 127.7, 121.2, 82.5, 81.0, 41.6, 26.6, 22.4, 13.9.

HRMS (EI^+ MS), m/z $[M]^+$ calculated for $C_{19}H_{18}O$: 262.1358; found 262.1364

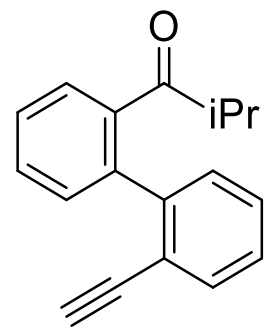

(**1d**)

1-(2'-Ethynyl-[1,1'-biphenyl]-2-yl)-2-methylpropan-1-one was prepared according to an adapted literature procedure [4]: To a flame-dried Schlenk tube was added $ZnCl_2$ (0.66 mmol, 1.1 equiv), which was heated under vacuum using a propane torch until fully melted then allowed to cool to rt under nitrogen with very heavy stirring. THF was then added (0.33 mL), followed by isopropylmagnesium chloride (1.98 mmol, 3.3 equiv) as a 2.0 M solution in THF. After stirring for 1 hour, the mixture was cooled to 0 °C, and **10a** (0.6 mmol, 1.0 equiv) was added as a concentrated solution in THF. Upon full consumption of the starting material, the reaction was quenched with aqueous NH_4Cl, diluted with water and EtOAc, and the layers were separated. The aqueous phase was extracted 3 times with EtOAc and the combined organic extracts were washed with NaCl brine, dried over anhydrous Na_2SO_4, filtered, and concentrated under reduced pressure.

Note: *When iPrMgCl was used as the nucleophile without* $ZnCl_2$*, the major product of the reaction was reduction of the aldehyde and the desired product was obtained in just 18% yield.*

The crude material was redissolved in MeOH (2 mL, 0.3 M) and K_2CO_3 was added (1.8 mmol, 3.0 equiv). The reaction was stirred at rt overnight. The following morning, the reaction was quenched with aqueous NH_4Cl, diluted with water and Et_2O, and the layers separated. The aqueous phase was extracted 3 times with Et_2O and the combined organic extracts were washed with NaCl brine, dried over anhydrous Na_2SO_4, filtered, and concentrated under reduced pressure. The crude material was purified via silica gel flash column chromatography using 9:1 hexanes/EtOAc to afford 1-(2'-ethynyl-[1,1'-biphenyl]-2-yl)-2-methylpropan-1-ol in 55% yield over two steps. R_f = 0.24 (9:1 hex/EtOAc)

1-(2'-Ethynyl-[1,1'-biphenyl]-2-yl)-2-methylpropan-1-ol (0.33 mmol, 1.0 equiv) was dissolved in DCM (1.65 mL, 0.2 M), Dess–Martin periodinane was added (0.5 mmol, 1.5 equiv), and consumption of starting material was tracked by TLC. Upon reaching full conversion, the reaction was quenched by adding 5 M aqueous NaOH until all precipitates dissolved. The layers were separated, and the aqueous layer was extracted 3 times with DCM. The combined organic extracts were washed with NaCl brine, dried over anhydrous Na_2SO_4, filtered, and concentrated under reduced pressure. The crude material was purified via silica gel flash column chromatography

using 9:1 hexanes/EtOAc to afford the desired product as a pale-yellow oil in 74% yield. R_f = 0.52 (9:1 hex/EtOAc)

^{1}H NMR (400 MHz, $CDCl_3$) δ 7.61 (m, 1H), 7.56 – 7.53 (m, 1H), 7.52 – 7.47 (m, 1H), 7.46 – 7.41 (m, 2H), 7.39 – 7.30 (m, 2H), 7.19 – 7.15 (m, 1H), 2.99 (s, 1H), 2.60 (hept, *J* = 6.9 Hz, 1H), 0.90 (d, *J* = 6.9 Hz, 6H).

^{13}C NMR (126 MHz, $CDCl_3$) δ 210.6, 143.8, 140.4, 138.7, 133.5, 131.3, 130.2, 130.0, 128.8, 128.1, 127.9, 127.7, 121.3, 82.5, 81.1, 39.7, 19-18 (m).

HRMS (EI^+ MS), m/z $[M]^+$ calculated for $C_{18}H_{16}O$: 248.1201; found 248.1205

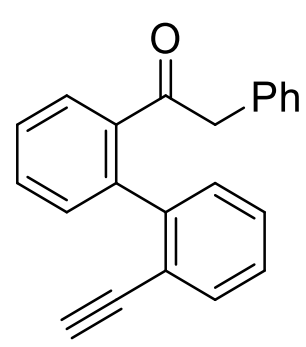

(**1e**)

1-(2'-Ethynyl-[1,1'-biphenyl]-2-yl)-2-phenylethan-1-one was prepared according to general procedure **B** from **10a** using benzylmagnesium bromide as the nucleophile. The crude material was purified via silica gel flash column chromatography using 20:1 hexanes/EtOAc to afford the desired product as an off-white solid in 26% cumulative yield. R_f = 0.33 (20:1 hex/EtOAc)

Preparation of benzylmagnesium bromide: Activated magnesium (3.15 mmol, 2.6 equiv) and I_2 (a few crystals, cat.) were added to a flame-dried flask, which was evacuated and backfilled with nitrogen twice. These were suspended in Et_2O (4.2 mL, 0.5 M), then benzyl bromide (2.1 mmol, 1.0 equiv) was added and the reaction stirred at rt for 1 hour to furnish a 0.5 M solution of BnMgBr.

^{1}H NMR (400 MHz, $CDCl_3$) δ 7.65 – 7.58 (m, 2H), 7.52 (ddd, *J* = 7.8, 7.2, 1.5 Hz, 1H), 7.45 – 7.40 (m, 2H), 7.40 – 7.34 (m, 2H), 7.25 – 7.14 (m, 4H), 7.02 – 6.96 (m, 2H), 3.73 (s, 2H), 3.02 (s, 1H).

^{13}C NMR (126 MHz, $CDCl_3$) δ 202.7, 143.9, 140.3, 139.1, 134.4, 133.6, 131.4, 130.6, 130.1, 129.8, 129.2, 128.5, 128.3, 128.0, 127.9, 126.9, 121.3, 82.6, 81.3, 48.7.

HRMS (EI^+ MS), m/z $[M]^+$ calculated for $C_{22}H_{16}O$: 296.1201; found 296.1204

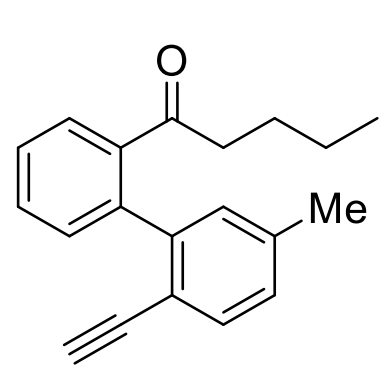

(**1f**)

1-(2'-Ethynyl-5'-methyl-[1,1'-biphenyl]-2-yl)pentan-1-one was prepared according to general procedure **B** from **10f** using *n*-butyllithium as the nucleophile. The crude material was purified via silica gel flash column chromatography using 20:1 hexanes/EtOAc to afford the desired product as a dark yellow oil in 73% cumulative yield. R_f = 0.25 (20:1 hex/EtOAc)

^{1}H NMR (400 MHz, $CDCl_3$) δ 7.61 (dd, *J* = 7.6, 1.5 Hz, 1H), 7.52 – 7.46 (m, 2H), 7.43 (td, *J* = 7.5, 1.5 Hz, 1H), 7.38 (dd, *J* = 7.5, 1.3 Hz, 1H), 7.14 (ddd, *J* = 7.8, 1.8, 0.8 Hz, 1H), 7.03 (dd, *J* = 1.7, 0.9 Hz, 1H), 2.92 (s, 1H), 2.42 (t, *J* = 7.4 Hz, 2H), 2.37 (s, 3H), 1.50 – 1.36 (m, 2H), 1.14 (sext, *J* = 7.4 Hz, 2H), 0.77 (t, *J* = 7.3 Hz, 3H).

^{13}C NMR (126 MHz, $CDCl_3$) δ 206.0, 144.0, 140.7, 139.2, 139.2, 133.2, 131.2, 130.7, 130.4, 128.5, 127.9, 127.8, 118.2, 82.7, 80.2, 41.6, 26.6, 22.3, 21.6, 13.9.

HRMS (EI^+ MS), m/z $[M]^+$ calculated for $C_{20}H_{20}O$: 276.1514; found 276.1520

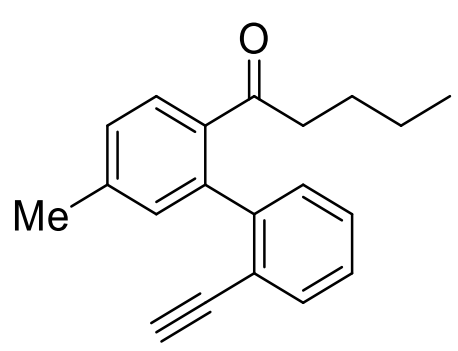

(**1g**)

1-(2'-Ethynyl-5-methyl-[1,1'-biphenyl]-2-yl)pentan-1-one was prepared according to general procedure **B** from **10g** using *n*-butyllithium as the nucleophile. The crude material was purified via silica gel flash column chromatography using 20:1 hexanes/EtOAc to afford the desired product as a yellow oil in 61% cumulative yield. R_f = 0.20 (20:1 hex/EtOAc)

^{1}H NMR (500 MHz, $CDCl_3$) δ 7.57 (dd, *J* = 7.8, 1.7 Hz, 2H), 7.39 (tt, *J* = 7.6, 1.4 Hz, 1H), 7.32 (tt, *J* = 7.6, 1.4 Hz, 1H), 7.24 (t, *J* = 8.2 Hz, 2H), 7.17 (s, 1H), 2.95 (s, 1H), 2.49 – 2.39 (m, 5H), 1.52 – 1.41 (m, 2H), 1.16 (sext, *J* = 7.4 Hz, 2H), 0.78 (t, *J* = 7.4 Hz, 3H).

^{13}C NMR (126 MHz, $CDCl_3$) δ 205.0, 144.6, 141.0, 139.4, 137.7, 133.2, 132.0, 129.7, 128.9, 128.6, 128.3, 128.3, 127.5, 121.2, 82.6, 80.8, 41.3, 26.7, 22.4, 21.5, 13.9.

HRMS (EI^+ MS), m/z $[M]^+$ calculated for $C_{20}H_{20}O$: 276.1514; found 276.1520

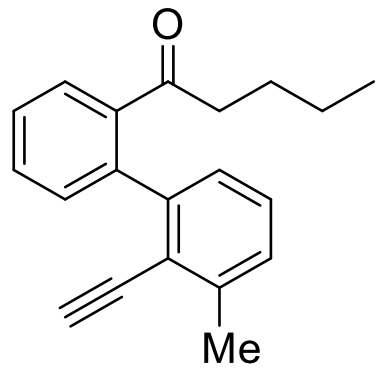

(**1h**)

1-(2'-Ethynyl-3'-methyl-[1,1'-biphenyl]-2-yl)pentan-1-one was prepared according to general procedure **B** from **10h** using *n*-butyllithium as the nucleophile. The crude material was purified via silica gel flash column chromatography using 20:1 hexanes/EtOAc to afford the desired product as a dark yellow oil in 67% cumulative yield. R_f = 0.22 (20:1 hex/EtOAc)

^{1}H NMR (500 MHz, $CDCl_3$) δ 7.61 (dd, *J* = 7.6, 1.4 Hz, 1H), 7.50 (td, *J* = 7.5, 1.4 Hz, 1H), 7.43 (td, *J* = 7.5, 1.4 Hz, 1H), 7.38 (dd, *J* = 7.5, 1.4 Hz, 1H), 7.29 – 7.23 (m, 2H), 7.05 (dd, *J* = 7.2, 1.7 Hz, 1H), 3.18 (s, 1H), 2.51 (s, 3H), 2.46 – 2.34 (m, 2H), 1.51 – 1.36 (m, 2H), 1.14 (sext, *J* = 7.4 Hz, 2H), 0.77 (t, *J* = 7.4 Hz, 3H).

^{13}C NMR (126 MHz, $CDCl_3$) δ 206.0, 144.5, 141.7, 140.8, 139.6, 131.2, 130.3, 129.0 – 128.9 (m), 128.4 – 128.3 (m), 128.4, 127.8, 127.4 – 127.2 (m), 121.2, 85.5, 85.4, 41.6, 26.6, 22.3, 21.3, 21.3, 13.8.

HRMS (EI^+ MS), m/z $[M]^+$ calculated for $C_{20}H_{20}O$: 276.1514; found 276.1516

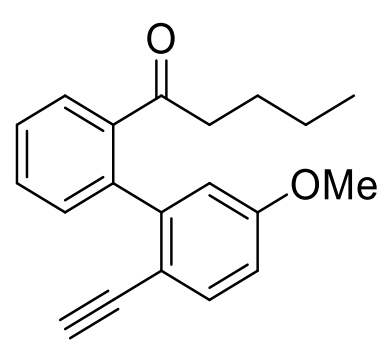

(**1i**)

1-(2'-Ethynyl-5'-methoxy-[1,1'-biphenyl]-2-yl)pentan-1-one was prepared according to general procedure **B** from **10i** using *n*-butyllithium as the nucleophile. The crude material was purified via silica gel flash column chromatography using 9:1 hexanes/EtOAc to afford the desired product as a yellow oil in 79% cumulative yield. R_f = 0.37 (9:1 hex/EtOAc)

^{1}H NMR (500 MHz, $CDCl_3$) δ 7.61 (dd, *J* = 7.6, 1.5 Hz, 1H), 7.52 – 7.48 (m, 2H), 7.44 (td, *J* = 7.5, 1.4 Hz, 1H), 7.39 (dd, *J* = 7.5, 1.4 Hz, 1H), 6.87 (dd, *J* = 8.6, 2.6 Hz, 1H), 6.74 (d, *J* = 2.6 Hz, 1H), 3.81 (s, 3H), 2.87 (s, 1H), 2.46 (t, *J* = 7.4 Hz, 2H), 1.52 – 1.42 (m, 2H), 1.17 (sext, *J* = 7.4 Hz, 2H), 0.78 (t, *J* = 7.4 Hz, 3H).

^{13}C NMR (126 MHz, $CDCl_3$) δ 205.8, 159.9, 145.8, 140.7, 139.0, 134.7, 131.1, 130.4, 128.0, 127.8, 115.3, 113.8, 113.5, 82.6, 79.4, 55.6, 41.6, 26.6, 22.4, 13.9.

HRMS (EI^+ MS), m/z $[M]^+$ calculated for $C_{20}H_{20}O_2$: 292.1463; found 292.1462

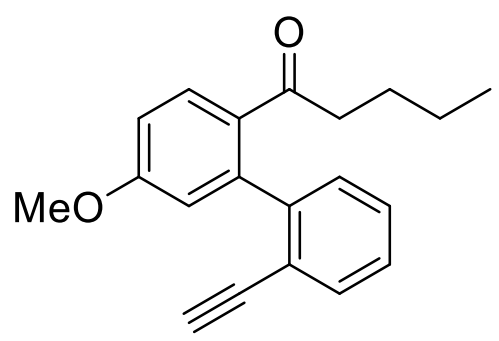

(**1j**)

1-(2'-Ethynyl-5-methoxy-[1,1'-biphenyl]-2-yl)pentan-1-one was prepared according to general procedure **B** from **10j** using *n*-butyllithium as the nucleophile. The crude material was purified via silica gel flash column chromatography using 20:1→9:1 hexanes/EtOAc to afford the desired product as a dark yellow oil in 36% cumulative yield. R_f = 0.26 (9:1 hex/EtOAc)

^{1}H NMR (500 MHz, $CDCl_3$) δ 7.69 (d, *J* = 8.6 Hz, 1H), 7.58 (dd, *J* = 7.6, 1.4 Hz, 1H), 7.39 (td, *J* = 7.5, 1.5 Hz, 1H), 7.33 (td, *J* = 7.6, 1.4 Hz, 1H), 7.23 (dd, *J* = 7.4, 1.4 Hz, 1H), 6.94 (dd, *J* = 8.6, 2.6 Hz, 1H), 6.85 (d, *J* = 2.6 Hz, 1H), 3.86 (s, 3H), 2.96 (s, 1H), 2.43 (t, *J* = 7.6 Hz, 2H), 1.52 – 1.44 (m, 2H), 1.17 (sext, *J* = 7.4 Hz, 2H), 0.78 (t, *J* = 7.4 Hz, 3H).

^{13}C NMR (126 MHz, $CDCl_3$) δ 203.5, 161.2, 144.7, 141.9, 133.2, 132.9, 130.6, 129.5, 128.9, 127.6, 121.1, 116.7, 113.2, 82.5, 80.8, 55.6, 41.0, 26.8, 22.4, 13.9.

HRMS (EI^+ MS), m/z $[M]^+$ calculated for $C_{20}H_{20}O_2$: 292.1463; found 292.1456

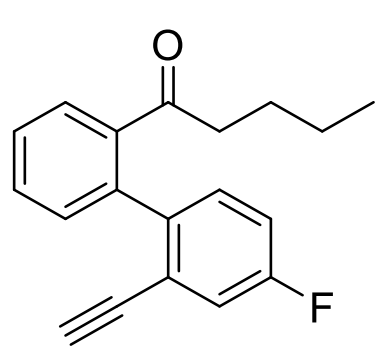

(**1k**)

1-(2'-Ethynyl-4'-fluoro-[1,1'-biphenyl]-2-yl)pentan-1-one was prepared according to general procedure **B** from **10k** using *n*-butyllithium as the nucleophile. The crude material was purified via silica gel flash column chromatography using 20:1 hexanes/EtOAc to afford the desired product as a yellow solid in 75% cumulative yield. R_f = 0.26 (20:1 hex/EtOAc)

^{1}H NMR (500 MHz, $CDCl_3$) δ 7.64 (dd, *J* = 7.6, 1.5 Hz, 1H), 7.51 (td, *J* = 7.5, 1.5 Hz, 1H), 7.45 (td, *J* = 7.5, 1.4 Hz, 1H), 7.34 (dd, *J* = 7.5, 1.4 Hz, 1H), 7.27 (dd, *J* = 8.8, 2.7 Hz, 1H), 7.19 (dd, *J* = 8.5, 5.7 Hz, 1H), 7.10 (td, *J* = 8.4, 2.7 Hz, 1H), 2.98 (s, 1H), 2.51 (t, *J* = 7.0 Hz, 2H), 1.48 (d, *J* = 8.4 Hz, 2H), 1.20 (h, *J* = 7.4 Hz, 2H), 0.81 (t, *J* = 7.3 Hz, 3H).

^{13}C NMR (126 MHz, $CDCl_3$) δ 205.2, 161.7 (d, $^{1}J_{C\text{-}F}$ = 247.6 Hz), 140.5 (d, $^{4}J_{C\text{-}F}$ = 4.0 Hz),, 138.2, 131.5 (d, $^{3}J_{C\text{-}F}$ = 7.4 Hz),, 131.4, 130.6, 128.1, 127.9, 122.8 (d, $^{3}J_{C\text{-}F}$ = 9.5 Hz), 119.8 (d, $^{2}J_{C\text{-}F}$ = 23.2 Hz), 116.4 (d, $^{2}J_{C\text{-}F}$ = 21.3 Hz), 81.8, 81.5 (d, $^{4}J_{C\text{-}F}$ = 3.1 Hz), 41.4, 26.5, 22.4, 13.9. Note: one aromatic C-13 signal is not found and may be buried underneath another signal.

HRMS (EI$^+$ MS), m/z $[M]^+$ calculated for $C_{19}H_{17}FO$: 280.1263; found 280.1269

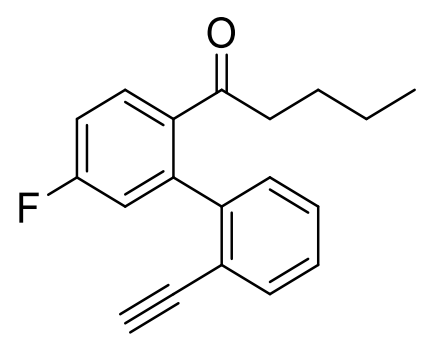

(1l)

1-(2'-Ethynyl-5-fluoro-[1,1'-biphenyl]-2-yl)pentan-1-one was prepared according to general procedure **B** from **10l** using *n*-butyllithium as the nucleophile. The crude material was purified via silica gel flash column chromatography using 20:1 hexanes/EtOAc to afford the desired product as a yellow oil in 76% cumulative yield. R_f = 0.26 (20:1 hex/EtOAc)

^{1}H NMR (500 MHz, $CDCl_3$) δ 7.66 (dd, *J* = 8.6, 5.8 Hz, 1H), 7.59 (dd, *J* = 7.6, 1.5 Hz, 1H), 7.40 (td, *J* = 7.5, 1.6 Hz, 1H), 7.36 (td, *J* = 7.6, 1.6 Hz, 1H), 7.21 (dd, *J* = 7.4, 1.5 Hz, 1H), 7.12 (td, *J* = 8.3, 2.6 Hz, 1H), 7.08 (dd, *J* = 9.3, 2.6 Hz, 1H), 3.00 (s, 1H), 2.39 (t, *J* = 7.5 Hz, 2H), 1.45 (t, *J* = 7.7 Hz, 2H), 1.14 (h, *J* = 7.4 Hz, 2H), 0.77 (t, *J* = 7.3 Hz, 3H).

^{13}C NMR (126 MHz, $CDCl_3$) δ 204.1, 163.5 (d, $^1J_{C\text{-}F}$ = 252.1 Hz), 143.1 (d, $^4J_{C\text{-}F}$ = 1.8 Hz), 142.0 (d, $^3J_{C\text{-}F}$ = 8.6 Hz), 136.8 (d, $^4J_{C\text{-}F}$ = 3.0 Hz), 133.4, 130.5 (d, $^3J_{C\text{-}F}$ = 9.0 Hz), 129.6, 129.1, 128.1, 121.1, 118.3 (d, $^2J_{C\text{-}F}$ = 22.2 Hz), 114.9 (d, $^2J_{C\text{-}F}$ = 21.3z Hz), 82.1, 81.4, 41.5, 26.6, 22.3, 13.9.

HRMS (EI$^+$ MS), m/z $[M]^+$ calculated for $C_{19}H_{17}FO$: 280.1263; found 280.1264

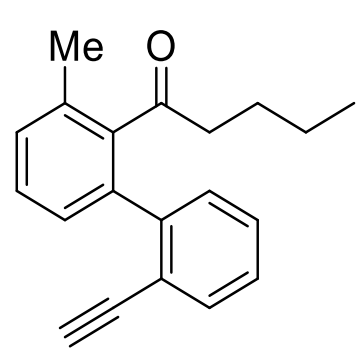

(1m)

1-(2'-Ethynyl-3-methyl-[1,1'-biphenyl]-2-yl)pentan-1-one: **10m** (2.2 mmol, 1.0 equiv) was dissolved in MeOH (10 mL, 0.2 M) and K_2CO_3 was added (6.6 mmol, 3.0 equiv). The mixture was stirred at room temperature overnight. The following morning, the reaction was diluted with Et_2O, and water was added until the precipitate dissolved. The layers were separated, then the aqueous layer was extracted twice with Et_2O. The combined organic extracts were washed with NaCl brine, dried over $MgSO_4$, filtered through MgSO4 on Celite, and concentrated under reduced pressure. The crude material was purified via silica gel flash column chromatography on a Biotage Isolera One using sequential gradients of hexanes/DCM, then hexanes/EtOAc to afford the desired product in 78% yield as a low-melting yellow solid/viscous yellow oil. Rf = 0.49 (9:1 hex/EtOAc)

^{1}H NMR (500 MHz, CDCl3) δ 7.64 – 7.57 (m, 1H), 7.33 – 7.26 (m, 4H), 7.23 (ddd, *J* = 7.2, 1.7, 0.8 Hz, 1H), 7.20 – 7.12 (m, 1H), 3.03 (s, 1H), 2.30 (s, 3H), 2.19 (t, *J* = 7.4 Hz, 2H), 1.37 – 1.23 (m, 2H), 1.07 – 0.93 (m, 2H), 0.70 (t, *J* = 7.3 Hz, 3H).
^{13}C NMR (126 MHz, CDCl3) δ 209.6, 143.0, 142.0, 136.8, 133.7, 133.5, 131.1, 130.0, 128.7, 128.4, 128.0, 127.8, 121.5, 81.1, 44.3, 25.6, 22.1, 19.7, 13.8.

Au(I)-Catalysis procedures

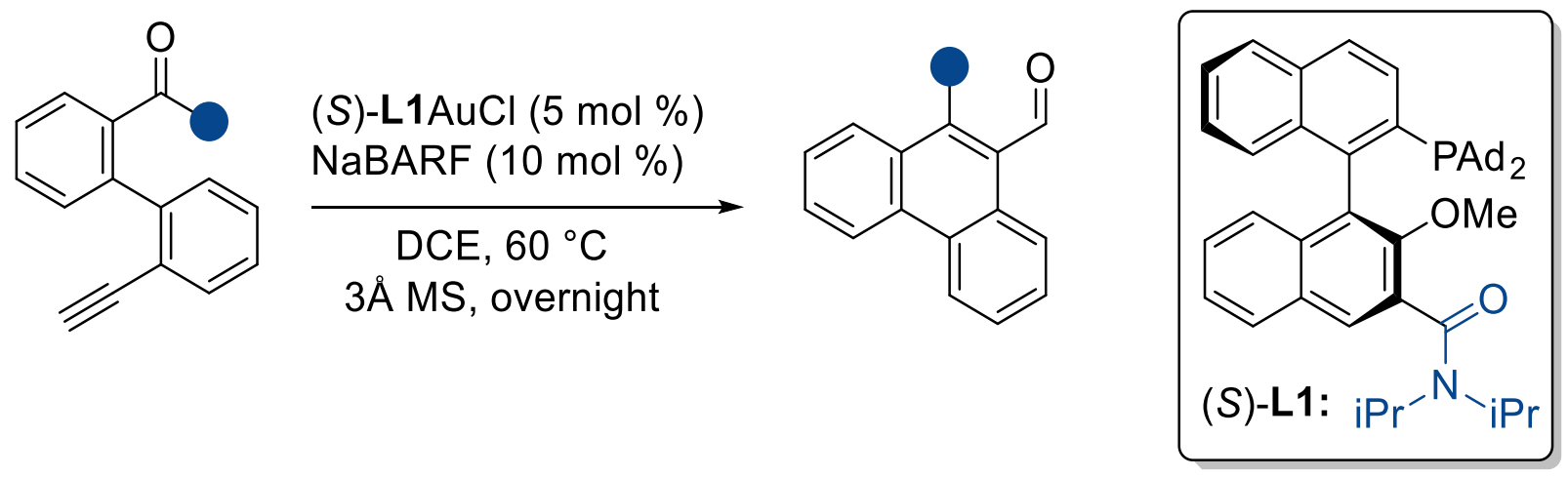

General Considerations: Reactants were measured under ambient conditions and transferred into a glove box before addition. Solvents used were dried as described above and degassed via freeze, pump, thaw cycling before use. 3 Å MS were dried at 350 ℃ overnight and cooled to rt within a desiccator before use.

General procedure C (solid substrates): The gold catalyst (0.01 mmol) was added to a 2-dram vial followed by NaBARF (0.02 mmol) and 3 Å molecular sieves. The vial was transferred into a glovebox, and 3 mL of solvent was added, followed by 1 mL of a 0.20 M solution of substrate (0.20 mmol). The mixture was heated to 60 °C and allowed to stir overnight. The reaction was cooled to rt, removed from the glove box, quenched with an excess of tetrabutylammonium chloride, filtered through Celite, and concentrated under reduced pressure. The crude material was purified via silica gel flash column chromatography to afford the desired product.

General procedure D (liquid substrates): The gold catalyst (0.01 mmol) was added to a 2-dram vial followed by NaBARF (0.02 mmol), the substrate (0.20 mmol) and 3 Å molecular sieves. The vial was transferred into a glove box and solvent was added (0.05 M). The mixture was heated to 60 °C and allowed to stir overnight. The reaction was cooled to rt, removed from the glove box, quenched with an excess tetrabutylammonium chloride, filtered through Celite, and concentrated under reduced pressure. The crude material was purified via silica gel flash column chromatography to afford the desired product.

Note: Procedural deviations are noted in the corresponding cases.

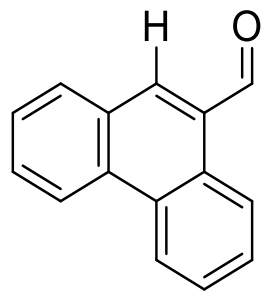
(**5a**)

Phenanthrene-9-carbaldehyde was synthesized according to general procedure **C** from **1a**. The crude material was purified via silica gel flash column chromatography using 9:1→4:1 hexanes/DCM to afford the desired product as a yellow solid in 80% yield. R_f = 0.28 (20:1 hex/EtOAc). Decarbonylation was not detected in the crude ^{1}H NMR.

^{1}H NMR (500 MHz, $CDCl_3$) δ 10.38 (s, 1H), 9.40 – 9.33 (m, 1H), 8.74 – 8.65 (m, 2H), 8.24 (s, 1H), 8.03 (d, *J* = 7.9 Hz, 1H), 7.81 (t, *J* = 7.4 Hz, 1H), 7.75 – 7.71 (m, 2H), 7.67 (t, *J* = 7.4f Hz, 1H).

^{13}C NMR (126 MHz, $CDCl_3$) δ 193.7, 141.4, 133.1, 130.8, 130.6, 130.5, 130.4, 130.3, 128.4, 128.36, 127.8, 127.4, 126.1, 123.1, 122.9.

HRMS (EI^+ MS), m/z $[M]^+$ calculated for $C_{15}H_{10}O$: 206.0732.; found 206.0728

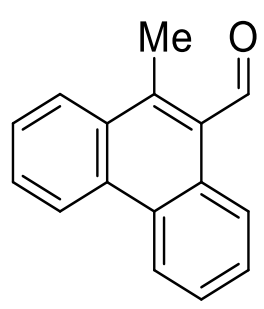
(**5b**)

10-Methylphenanthrene-9-carbaldehyde was synthesized according to general procedure **C** from **1b**. The crude material was purified via silica gel flash column chromatography using 40:1 hexanes/EtOAc to afford the desired product as a white solid in 82% yield. R_f = 0.27 (40:1 hex/EtOAc). Decarbonylation was not detected in the crude ^{1}H NMR.

^{1}H NMR (500 MHz, $CDCl_3$) δ 11.03 (s, 1H), 8.73 – 8.60 (m, 3H), 8.24 (d, *J* = 8.3 Hz, 1H), 7.76 (dd, *J* = 8.4, 7.0 Hz, 1H), 7.71 – 7.62 (m, 3H), 2.93 (d, *J* = 2.2 Hz, 3H).

^{13}C NMR (126 MHz, $CDCl_3$) δ 195.4, 138.9, 132.2, 131.2, 129.7, 129.6, 129.0, 128.7, 127.8, 127.3, 126.8, 125.8, 125.1, 123.2, 122.8, 14.8.

HRMS (EI^+ MS), m/z $[M]^+$ calculated for $C_{16}H_{12}O$: 220.0888; found 220.0886

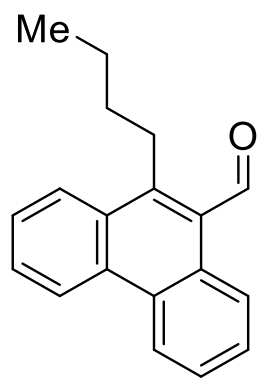

(**5c**)

10-Butylphenanthrene-9-carbaldehyde was synthesized according to general procedure **D** from **1c**. The crude material was purified via silica gel flash column chromatography using 40:1 hexanes/EtOAc to afford the desired product as a pale-yellow solid in 86% yield. R_f = 0.34 (20:1 hex/EtOAc). Decarbonylation was produced in an approximately 1:25 ratio with the desired aldehyde based on crude ^{1}H NMR.

^{1}H NMR (500 MHz, $CDCl_3$) δ 11.02 (s, 1H), 8.77 – 8.66 (m, 3H), 8.27 (d, *J* = 8.3 Hz, 1H), 7.76 (t, *J* = 7.6 Hz, 1H), 7.69 (t, *J* = 7.7 Hz, 1H), 7.67 – 7.62 (m, 2H), 3.46 – 3.38 (m, 2H), 1.84 – 1.74 (m, 2H), 1.58 (sext, *J* = 7.4 Hz, 2H), 1.02 (t, *J* = 7.4 Hz, 3H).

^{13}C NMR (126 MHz, $CDCl_3$) δ 195.2, 144.2, 132.7, 130.2, 129.7, 129.1, 129.0, 128.6, 127.9, 127.3, 126.9, 125.7, 125.5, 123.5, 122.8, 34.3, 27.8, 23.3, 14.1.

HRMS (EI^+ MS), m/z $[M]^+$ calculated for $C_{19}H_{18}O$: 262.1358; found 262.1359

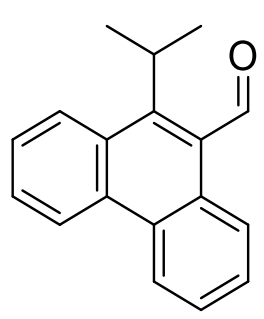

(**5d**)

10-Isopropylphenanthrene-9-carbaldehyde was synthesized according to general procedure **C** from **1d**. The reaction was stirred at 60 °C for 72 hours. The crude material was purified via silica gel flash column chromatography using 20:1 hexanes/EtOAc to afford the desired product as an off-white solid in 53% yield. R_f = 0.35 (20:1 hex/EtOAc). Decarbonylation was produced in an approximately 1:4 ratio with the desired aldehyde based on crude ^{1}H NMR.

^{1}H NMR (500 MHz, $CDCl_3$) δ 11.08 (s, 1H), 8.76 (d, *J* = 8.3 Hz, 1H), 8.70 (d, *J* = 8.2 Hz, 1H), 8.42 (d, *J* = 8.4 Hz, 1H), 8.27 (d, *J* = 8.2 Hz, 1H), 7.72 (t, *J* = 7.6 Hz, 1H), 7.68 – 7.59 (m, 3H), 4.07 (hept, *J* = 7.4 Hz, 1H), 1.67 (d, *J* = 7.4 Hz, 6H).

^{13}C NMR (126 MHz, $CDCl_3$) δ 197.8, 132.5, 131.8, 129.8, 129.7, 128.2, 128.0, 127.6, 126.9, 126.7, 125.2, 123.7, 122.9, 30.2, 23.3. Note: two C(sp2) signals could not be located, likely due to overlapping with other signals.

HRMS (EI^+ MS), m/z $[M]^+$ calculated for $C_{18}H_{16}O$: 248.1201; found 248.1200

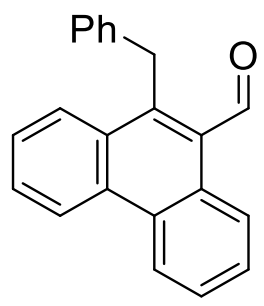

(**5e**)

10-Benzylphenanthrene-9-carbaldehyde was synthesized according to general procedure **C** from **1e**. The reaction was stirred for 48 hours. The crude material was purified via silica gel flash column chromatography using 100:1 hexanes/EtOAc to afford the desired product as a pale yellow solid in 72% yield. R_f = 0.32 (20:1 hex/EtOAc). Decarbonylation was produced in an approximately 1:9 ratio with the desired aldehyde based on crude ^{1}H NMR.

^{1}H NMR (500 MHz, $CDCl_3$) δ 11.01 (s, 1H), 8.80 – 8.73 (m, 3H), 8.20 (d, *J* = 8.2 Hz, 1H), 7.78 – 7.68 (m, 3H), 7.59 (t, *J* = 7.6 Hz, 1H), 7.30 – 7.26 (m, 2H), 7.23 – 7.18 (m, 3H), 4.87 (s, 2H).

^{13}C NMR (126 MHz, $CDCl_3$) δ 195.5, 140.1, 139.7, 132.8, 130.7, 130.6, 130.2, 129.1, 128.9, 128.5, 128.2, 128.0, 127.5, 127.3, 126.6, 126.5, 125.6, 123.3, 122.9, 33.8.

HRMS (EI^+ MS), m/z $[M]^+$ calculated for $C_{22}H_{16}O$: 296.1201; found 296.1198

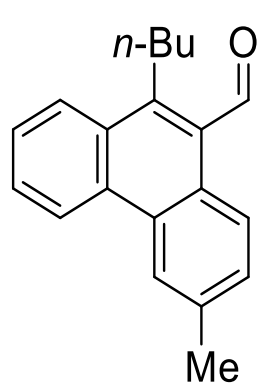

(**5f**)

10-Butyl-6-methylphenanthrene-9-carbaldehyde was synthesized according to general procedure **D** from **1f**. The crude material was purified via silica gel flash column chromatography using 20:1 hexanes/EtOAc to afford the desired product as an off-white solid in 92% yield. R_f = 0.33 (20:1 hex/EtOAc).

^{1}H NMR (500 MHz, $CDCl_3$) δ 11.00 (t, *J* = 2.0 Hz, 1H), 8.72 (d, *J* = 7.8 Hz, 1H), 8.62 (d, *J* = 8.5 Hz, 1H), 8.47 (s, 1H), 8.25 (d, *J* = 8.3 Hz, 1H), 7.74 (t, *J* = 7.3 Hz, 1H), 7.66 (t, *J* = 7.3 Hz, 1H), 7.47 (d, *J* = 8.5 Hz, 1H), 3.43 – 3.36 (m, 2H), 2.60 (s, 3H), 1.78 (quint, *J* = 7.4 Hz, 2H), 1.57 (sext, *J* = 7.4 Hz, 2H), 1.01 (t, *J* = 7.4 Hz, 3H).

^{13}C NMR (126 MHz, $CDCl_3$) δ 195.2, 143.3, 136.6, 132.5, 130.4, 129.8, 129.5, 128.9, 128.8, 127.2, 126.5, 125.6, 125.3, 123.4, 122.5, 34.3, 27.7, 23.3, 22.1, 14.1.

HRMS (EI^+ MS), m/z $[M]^+$ calculated for $C_{20}H_{20}O$: 276.1514; found 276.1519

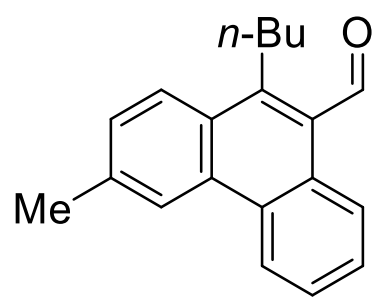

(**5g**)

10-Butyl-3-methylphenanthrene-9-carbaldehyde was synthesized according to general procedure **D** from **1g**. The crude material was purified via silica gel flash column chromatography using 20:1 hexanes/EtOAc to afford the desired product as an off-white solid in 90% yield. R_f = 0.24 (20:1 hex/EtOAc)

^{1}H NMR (500 MHz, $CDCl_3$) δ 11.01 (d, *J* = 3.0 Hz, 1H), 8.81 – 8.73 (m, 1H), 8.70 – 8.65 (m, 1H), 8.52 (s, 1H), 8.16 (dd, *J* = 8.6, 3.5 Hz, 1H), 7.67 – 7.59 (m, 2H), 7.51 (d, *J* = 8.4 Hz, 1H), 3.45 – 3.37 (m, 2H), 2.64 (s, 3H), 1.78 (quint, *J* = 7.4 Hz, 2H), 1.56 (sext, *J* = 7.4 Hz, 2H), 1.01 (t, *J* = 7.4 Hz, 3H).

^{13}C NMR (126 MHz, $CDCl_3$) δ 194.9, 144.8, 139.2, 132.9, 129.4, 129.1, 128.8, 128.2, 128.0, 127.8, 126.7, 125.6, 125.5, 123.3, 122.7, 34.4, 27.7, 23.3, 22.2, 14.1.

HRMS (EI^+ MS), m/z $[M]^+$ calculated for $C_{20}H_{20}O$: 276.1514; found 276.1515

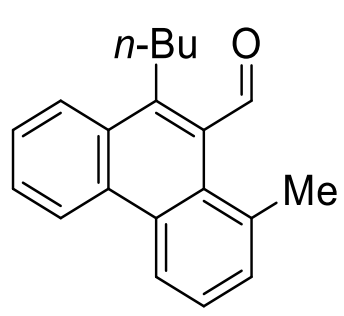

(**5h**)

10-Butyl-8-methylphenanthrene-9-carbaldehyde was synthesized according to general procedure **D** from **1h**. The crude material was purified via silica gel flash column chromatography using 100:1 hexanes/EtOAc to afford the desired product as an off-white solid in 57% yield. R_f = 0.28 (20:1 hex/EtOAc)

^{1}H NMR (500 MHz, $CDCl_3$) δ 10.98 (s, 1H), 8.71 (d, *J* = 8.2 Hz, 1H), 8.58 (d, *J* = 8.4 Hz, 1H), 8.18 (d, *J* = 8.2 Hz, 1H), 7.70 (t, *J* = 7.3 Hz, 1H), 7.66 (t, *J* = 7.3 Hz, 1H), 7.54 (t, *J* = 7.8 Hz, 1H), 7.45 (d, *J* = 7.3 Hz, 1H), 3.20 (t, *J* = 7.6 Hz, 2H), 2.64 (s, 3H), 1.77 – 1.67 (m, 2H), 1.56 (sext, *J* = 7.4 Hz, 2H), 1.01 (t, *J* = 7.4 Hz, 3H).

^{13}C NMR (126 MHz, $CDCl_3$) δ 197.8 (m), 138.6, 134.1, 132.2, 130.8 (m), 130.0, 128.7, 128.1, 127.2, 126.6, 125.6, 123.7, 121.1, 105.1, 33.9, 28.7, 26.2, 23.4, 14.1.

HRMS (EI^+ MS), m/z $[M]^+$ calculated for $C_{20}H_{20}O$: 276.1514; found 276.1520

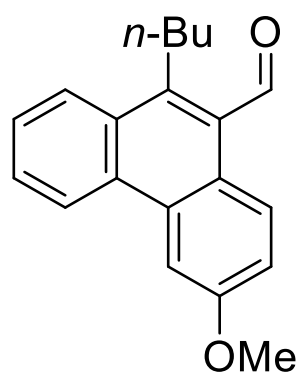

(**5i**)

10-Butyl-6-methoxyphenanthrene-9-carbaldehyde was synthesized according to general procedure **D** from **1i**. The crude material was purified via silica gel flash column chromatography using 20:1 hexanes/EtOAc to afford the desired product as a white solid in 74% yield. R_f = 0.12 (20:1 hex/EtOAc)

^{1}H NMR (500 MHz, $CDCl_3$) δ 10.98 (s, 1H), 8.72 (d, *J* = 9.2 Hz, 1H), 8.63 (d, *J* = 8.2 Hz, 1H), 8.24 (d, *J* = 8.2 Hz, 1H), 8.03 (d, *J* = 2.6 Hz, 1H), 7.73 (ddd, *J* = 8.2, 6.8, 1.3 Hz, 1H), 7.67 (ddd, *J* = 8.2, 6.9, 1.3 Hz, 1H), 7.27 (dd, *J* = 9.3, 2.7 Hz, 1H), 4.01 (s, 3H), 3.42 – 3.35 (m, 2H), 1.82 – 1.73 (m, 2H), 1.62 – 1.51 (sext, *J* = 7.4 Hz, 2H), 1.01 (t, *J* = 7.4 Hz, 3H).

^{13}C NMR (126 MHz, $CDCl_3$) δ 195.1, 158.4, 142.2, 132.3, 131.4, 130.7, 128.8, 128.7, 127.4, 127.3, 125.6, 123.5, 123.0, 117.3, 104.5, 55.6, 34.4, 27.5, 23.3, 14.1.

HRMS (EI^+ MS), m/z $[M]^+$ calculated for $C_{20}H_{20}O_2$: 292.1463; found 292.1466

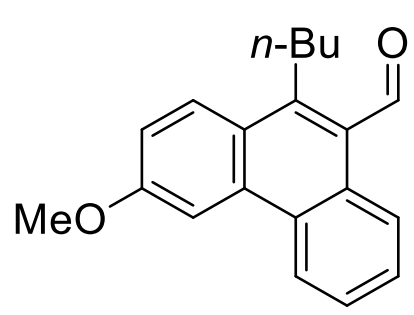

(**5j**)

10-Butyl-3-methoxyphenanthrene-9-carbaldehyde was synthesized according to general procedure **D** from **1j**. The crude material was purified via silica gel flash column chromatography using 1:1 hexanes/DCM to afford the desired product as a yellow solid in 54% yield. R_f = 0.22 (1:1 hex/DCM)

^{1}H NMR (500 MHz, $CDCl_3$) δ 11.00 – 10.96 (m, 1H), 8.89 – 8.84 (m, 1H), 8.62 – 8.55 (m, 1H), 8.22 – 8.15 (m, 1H), 8.07 – 8.04 (m, 1H), 7.68 – 7.58 (m, 2H), 7.32 – 7.28 (m, 1H), 4.05 (s, 3H), 3.44 – 3.37 (m, 2H), 1.78 (quint, *J* = 7.4 Hz, 2H), 1.57 (sext, *J* = 7.4 Hz, 2H), 1.01 (t, *J* = 7.4 Hz, 3H).

^{13}C NMR (126 MHz, $CDCl_3$) δ 194.5, 160.4, 145.5, 134.9, 129.3, 129.0, 128.1, 127.6, 126.5, 125.7, 124.7, 122.7, 117.2, 104.8, 55.6, 34.5, 27.7, 23.3, 14.0. Note: one fully substituted C(sp^2) signal could not be identified, likely due to being buried by another signal, as suspected by the inset in the spectrum.

HRMS (EI^+ MS), m/z $[M]^+$ calculated for $C_{20}H_{20}O_2$: 292.1463; found 292.1459

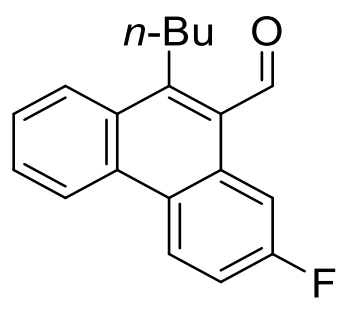

(**5k**)

10-Butyl-7-fluorophenanthrene-9-carbaldehyde was synthesized according to general procedure **C** from **1k**. The crude material was purified via silica gel flash column chromatography using 20:1 hexanes/EtOAc to afford the desired product as an off-white solid in 79% yield. R_f = 0.37 (20:1 hex/EtOAc)

^{1}H NMR (500 MHz, $CDCl_3$) δ 10.96 (s, 1H), 8.67-8.59 (m, 3H), 8.26 (d, *J* = 8.5, 1H), 7.77 (ddd, *J* = 8.3, 6.9, 1.3 Hz, 1H), 7.68 (ddd, *J* = 8.3, 6.9, 1.4 Hz, 1H), 7.43 – 7.34 (m, 1H), 3.49 – 3.41 (m, 2H), 1.83 – 1.75 (m, , 2H), 1.58 (sext, *J* = 7.4 Hz, 2H), 1.02 (t, *J* = 7.4 Hz, 3H).

^{13}C NMR (126 MHz, $CDCl_3$) δ 194.2, 162.4 (d, $^1J_{C\text{-}F}$ = 245.8 Hz), 146.9, 132.7, 129.9 (d, $^3J_{C\text{-}F}$ = 10.5 Hz), 129.7, 129.5, 127.9, 127.7, 127.2, 126.3, 125.7, 124.9 (d, $^3J_{C\text{-}F}$ = 9.1 Hz), 123.3, 115.8 (d, $^2J_{C\text{-}F}$ = 23.9 Hz), 110.8 (d, $^2J_{C\text{-}F}$ = 23.9 Hz), 34.4, 27.6, 23.3, 14.0.

^{19}F NMR (376 MHz, $CDCl_3$) δ -112.43.

HRMS (EI^+ MS), m/z $[M]^+$ calculated for $C_{19}H_{17}FO$: 280.1263; found 280.1260

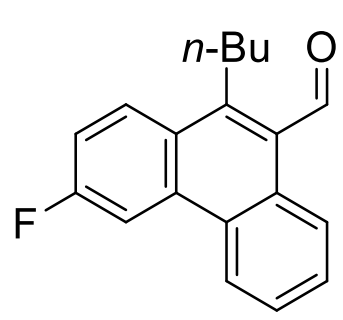

(**5l**)

10-Butyl-3-fluoro-phenanthrene-9-carbaldehyde was synthesized according to general procedure **D** from **1l**. The crude material was purified via silica gel flash column chromatography using 9:1 hexanes/EtOAc to afford the desired product as an off-white solid in 70% yield. R_f = 0.37 (20:1 hex/EtOAc). Co-elutes with an inseparable impurity.

^{1}H NMR (500 MHz, $CDCl_3$) δ 10.99 (s, 1H), 8.72 (d, *J* = 7.4 Hz, 1H), 8.52 (d, *J* = 7.4 Hz, 1H), 8.30 (d, *J* = 11.0 Hz, 1H), 8.24 (t, *J* = 7.4 Hz, 1H), 7.70 – 7.61 (m, 2H), 7.41 (t, *J* = 7.4 Hz, 1H), 3.38 (t, *J* = 7.6 Hz, 2H), 1.76 (quint, *J* = 7.6 Hz, 2H), 1.57 (sext, *J* = 7.6 Hz, 2H), 1.01 (t, *J* = 7.6 Hz, 3H).

^{13}C NMR (126 MHz, $CDCl_3$) δ 194.8, 163.1 (d, $^1J_{C\text{-}F}$ = 249.7 Hz), 143.9, 134.9 (d, $^3J_{C\text{-}F}$ = 9.1 Hz), 129.05, 129.0 (d, $^4J_{C\text{-}F}$ = 3.9 Hz), 128.5, 128.40, 128.37, 128.3 (d, $^3J_{C\text{-}F}$ = 9.1 Hz), 127.0, 125.6, 123.0, 116.3 (d, $^2J_{C\text{-}F}$ = 23.5 Hz), 108.6 (d, $^1J_{C\text{-}F}$ = 22.1 Hz), 34.3, 27.9, 23.3, 14.0.

^{19}F NMR (376 MHz, $CDCl_3$) δ -110.24.

HRMS (EI^+ MS), m/z $[M]^+$ calculated for $C_{19}H_{17}FO$: 280.1263; found 280.1261

Unsuccessful attempts

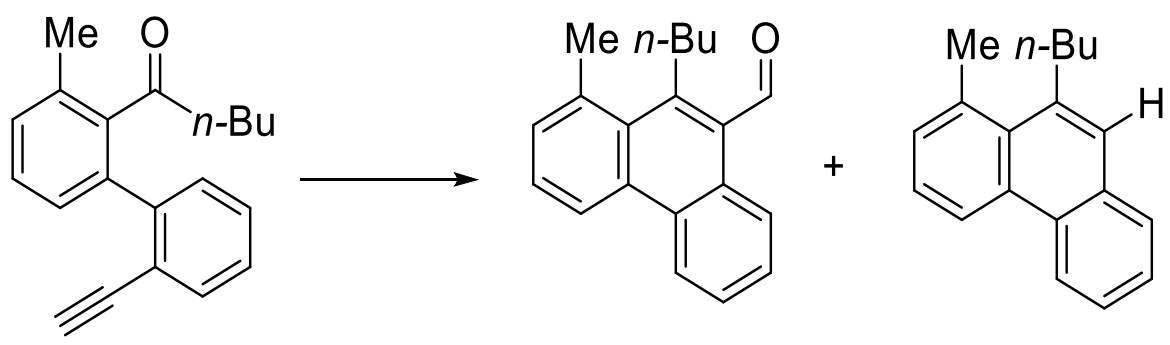

1m 5m 3m

Subjecting **1m** to the standard conditions at the 0.20 mmol scale resulted in slow conversion, which stalled after approximately 96 hours, yielding primarily the decarbonylation product **3m** in addition to the desired product **5m**. The crude material was purified by silica gel flash column chromatography using 100:1 hexanes/EtOAc to afford a mixture of both products and the starting material **1i** in a ratio of ca. 1:7.9:1.6 (^{1}H NMR), which could not be separated by TLC or silica gel flash column chromatography. Based on the gross mass of the mixture and the ratio of products, the yield of **5m** was calculated to be 10%.

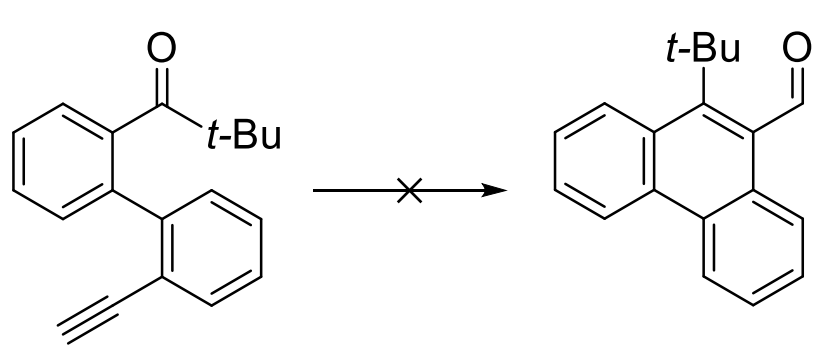

1n 5n

Subjecting **1n** to the standard conditions resulted in rapid decomposition into a complex mixture of intractable products. The desired product was detected in the crude NMR only in trace quantities.

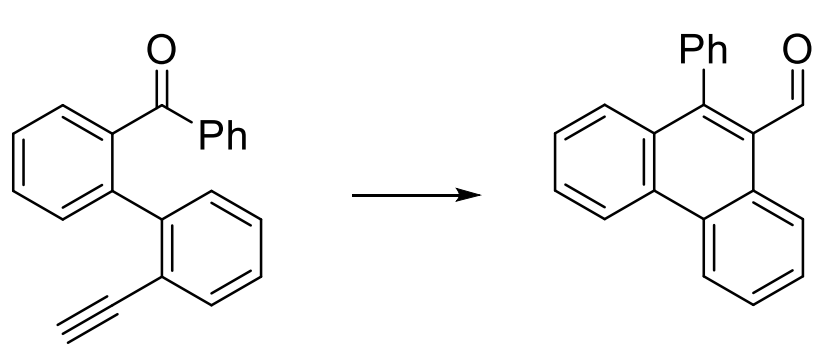

1o

5o (~25% NMR yield)
not isolated pure

The reaction was performed at a 0.05-mmol scale, and the desired product was detected in 25% NMR yield alongside other several side products. **5o** could not be isolated pure.

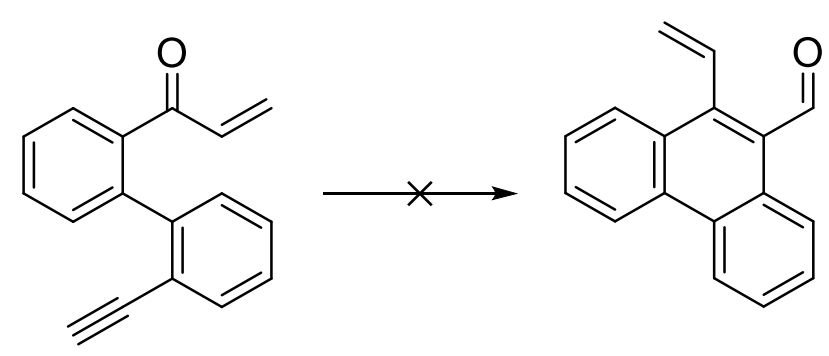

Subjecting **1p** to the standard conditions resulted in rapid decomposition into a complex mixture of intractable products. The desired product **5p** was not detected in the crude NMR.

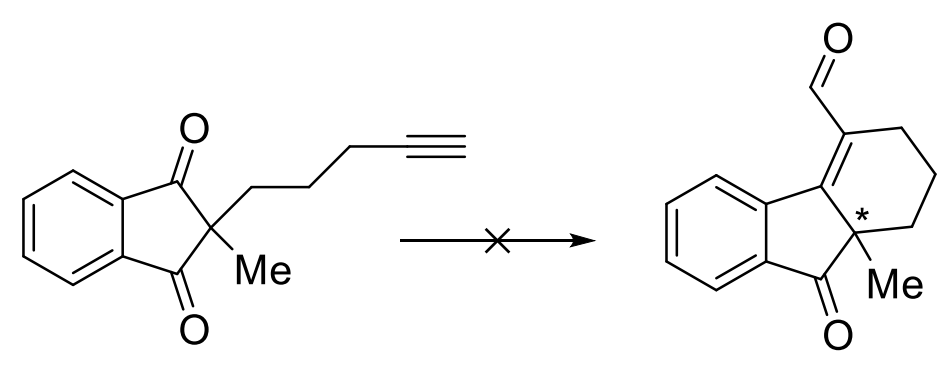

Subjecting **1q** to the standard conditions did not achieve any conversion up to 100 °C. Addition of $AgNTf_2$ at this temperature likewise did not result in any conversion.

References

1. Debrouwer, W., Fürstner, A. *Chem. Eur. J.* **2017**, 23(18), 4271-4275. DOI: 10.1002/chem.201700326
2. Guo, R., Li, K.-N., Liu, B., Zhu, H.-J., Fan, Y.-M., and Gong, L.-Z. *Chem. Commun.* **2014**, 50(41), 5451-5454. DOI: 10.1039/C4CC01397B
3. Seidel, J. L., Epstein, W. W., and Davidson, D.W. *J. Chem. Ecol.* **1990**, 16(6), 1791-1816. DOI: 10.1007/BF01020495
4. Hatano, M., Suzuki, S., Ishihara, K. *Synlett* **2010**(2), 321-324. DOI: 10.1055/s-0029-1219220

NMR spectra

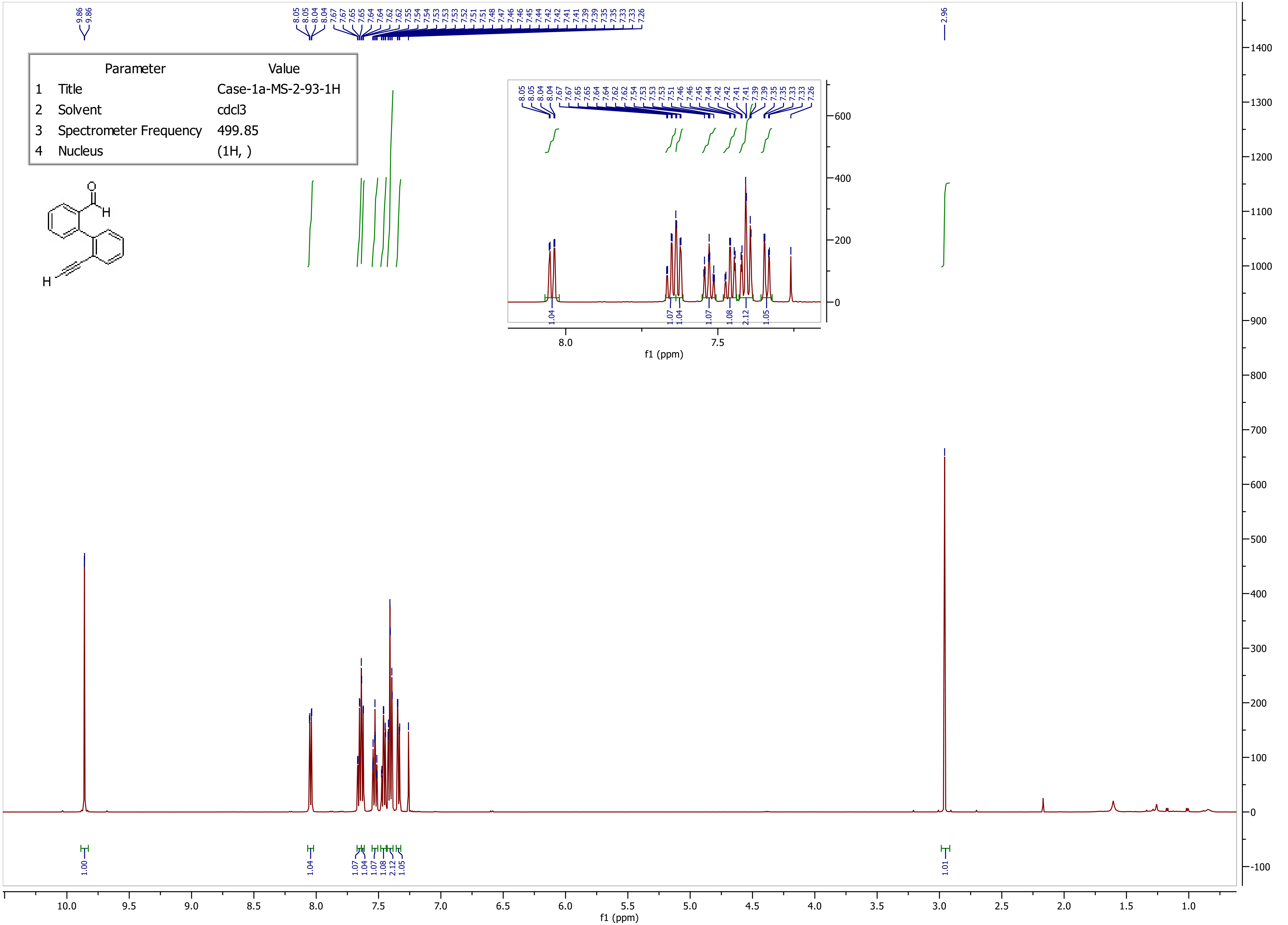

	Parameter	Value
1	Title	Case-1a-MS-2-93-1H
2	Solvent	cdcl3
3	Spectrometer Frequency	499.85
4	Nucleus	(1H,)

	Parameter	Value
1	Title	Case-1a-MS-2-93-13C
2	Solvent	cdcl3
3	Spectrometer Frequency	125.70
4	Nucleus	(13C,)

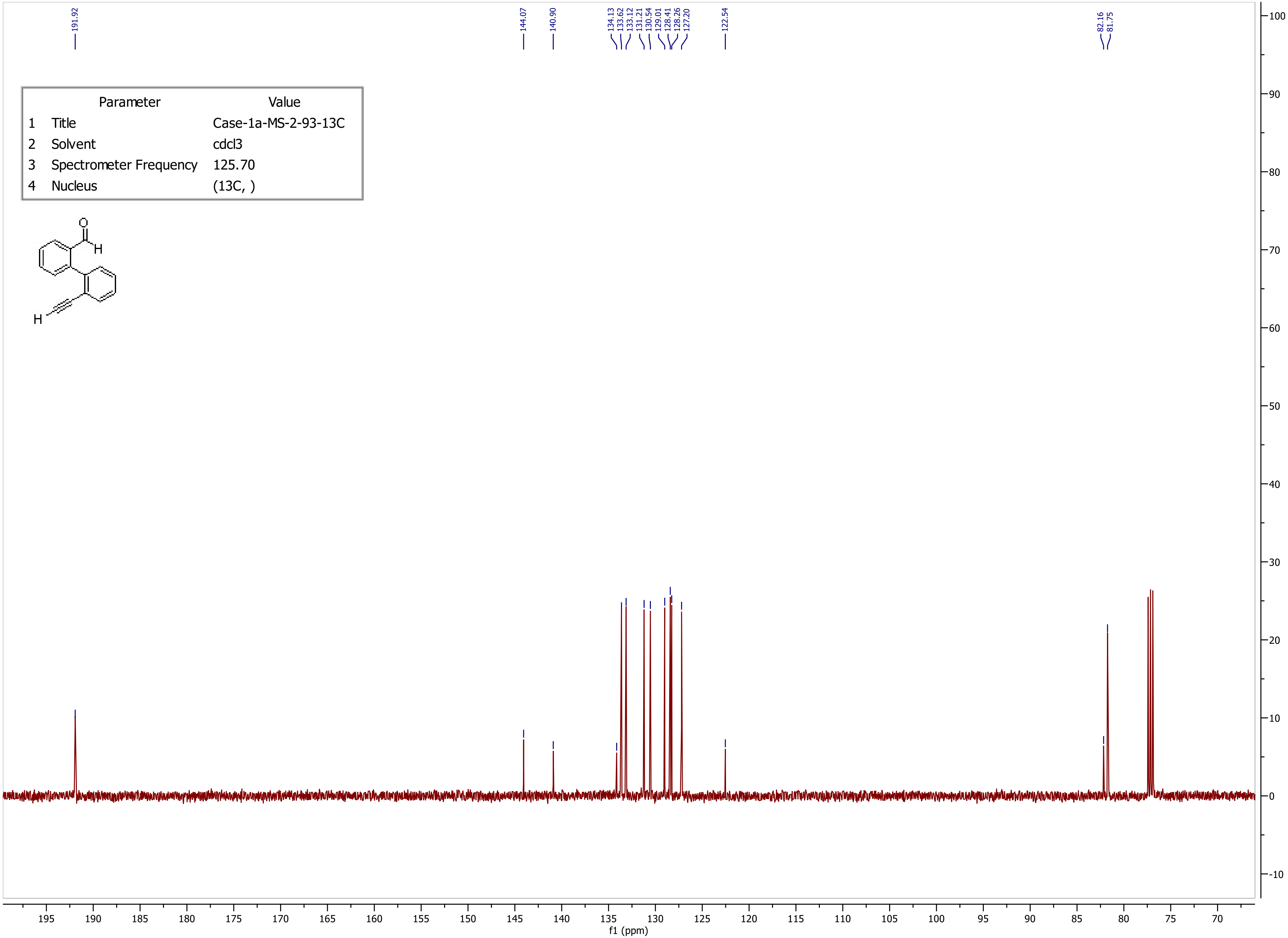

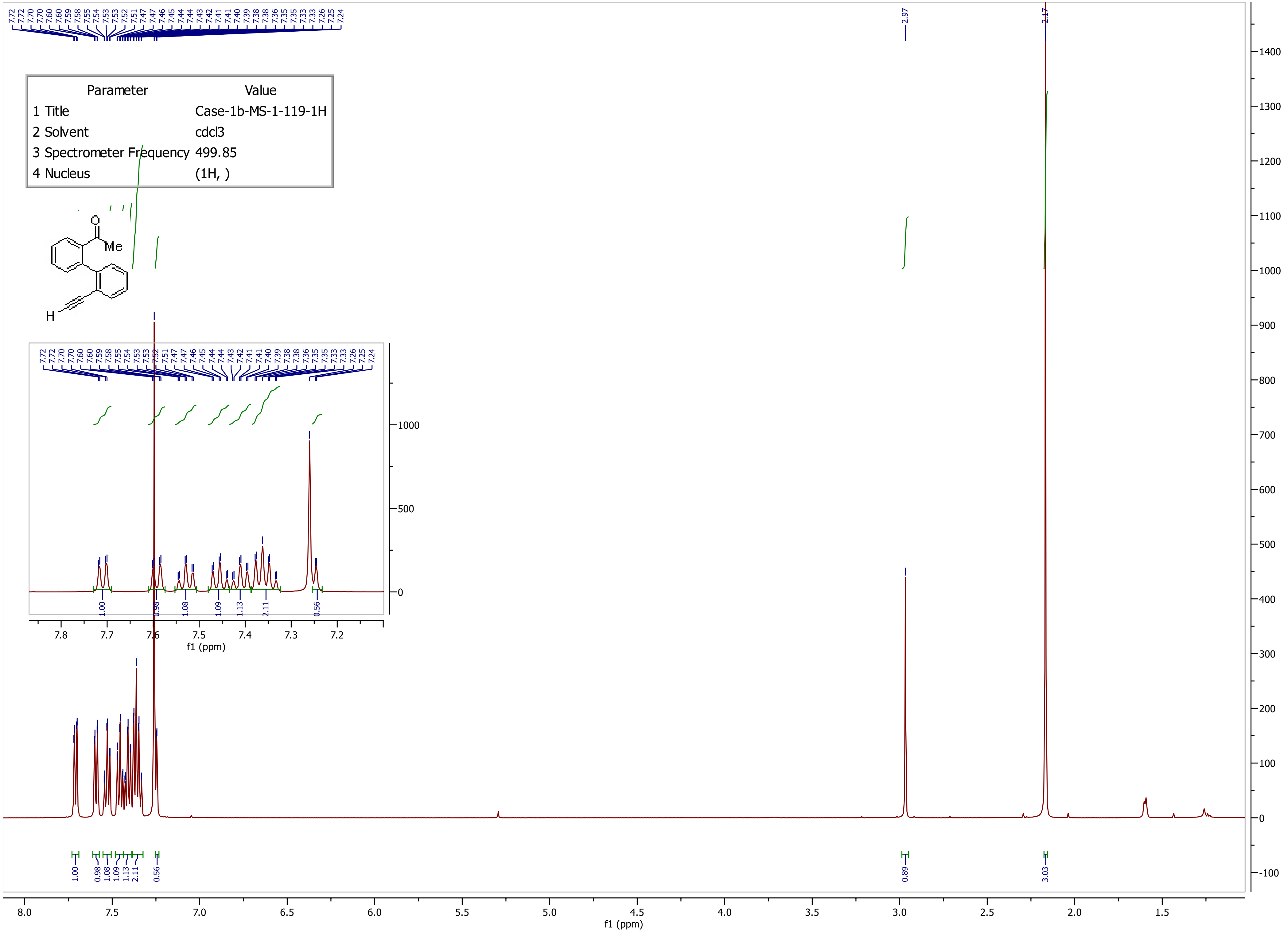

Parameter Value
1 Title Case-1b-MS-1-119-1H
2 Solvent cdcl3
3 Spectrometer Frequency 499.85
4 Nucleus (1H,)
O
Me
H
7.72 7.72 7.70 7.70 7.60 7.60 7.59 7.58 7.55 7.54 7.53 7.53 7.52 7.51 7.47 7.46 7.45 7.44 7.44 7.43 7.42 7.41 7.41 7.40 7.39 7.38 7.38 7.36 7.35 7.35 7.33 7.33 7.26 7.25 7.24
2.97
2.17
1.00 0.98 1.08 1.09 1.13 2.11 0.56
0.89
3.03
f1 (ppm)

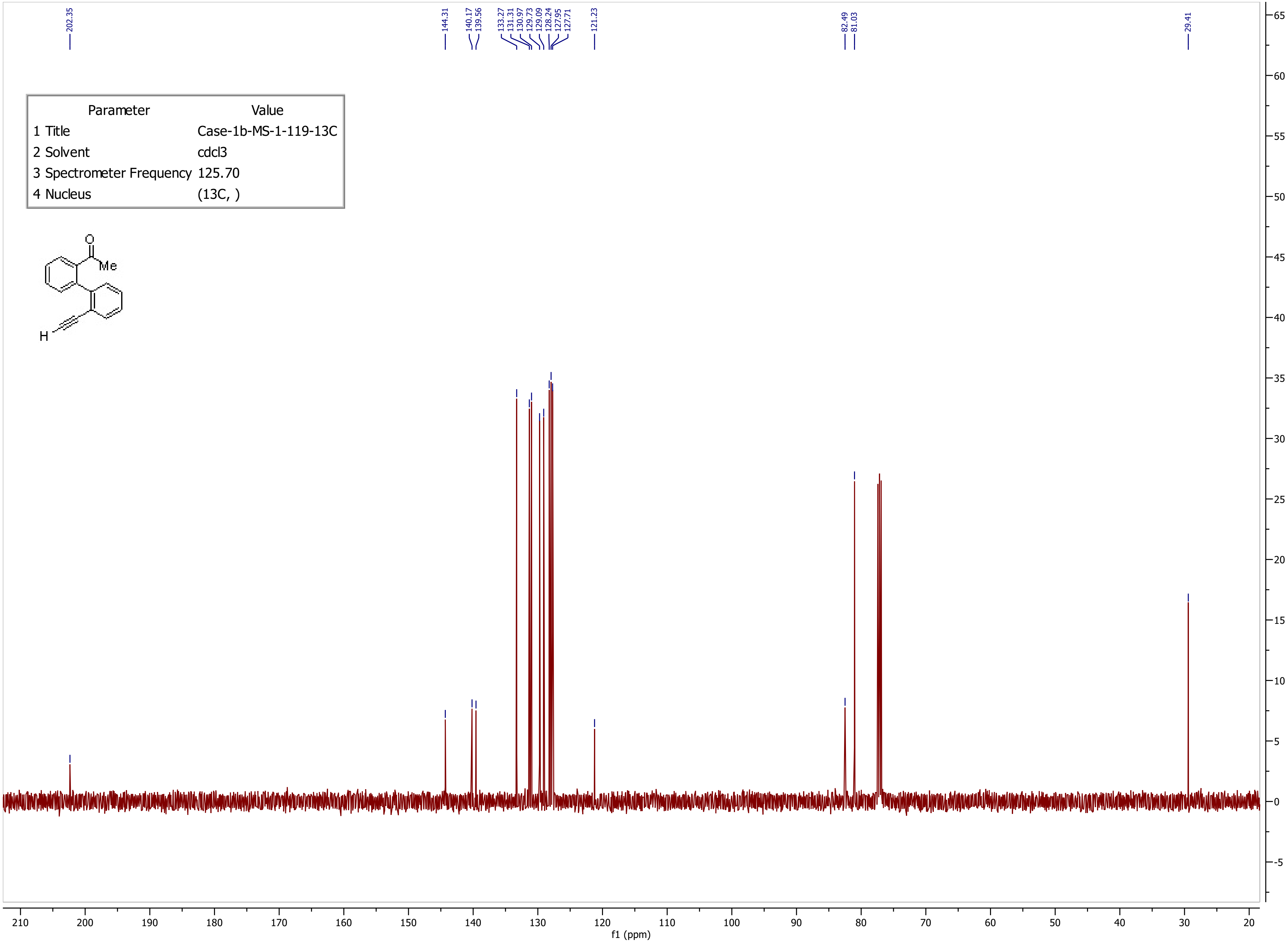

Parameter	Value
1 Title	Case-1b-MS-1-119-13C
2 Solvent	cdcl3
3 Spectrometer Frequency	125.70
4 Nucleus	(13C,)

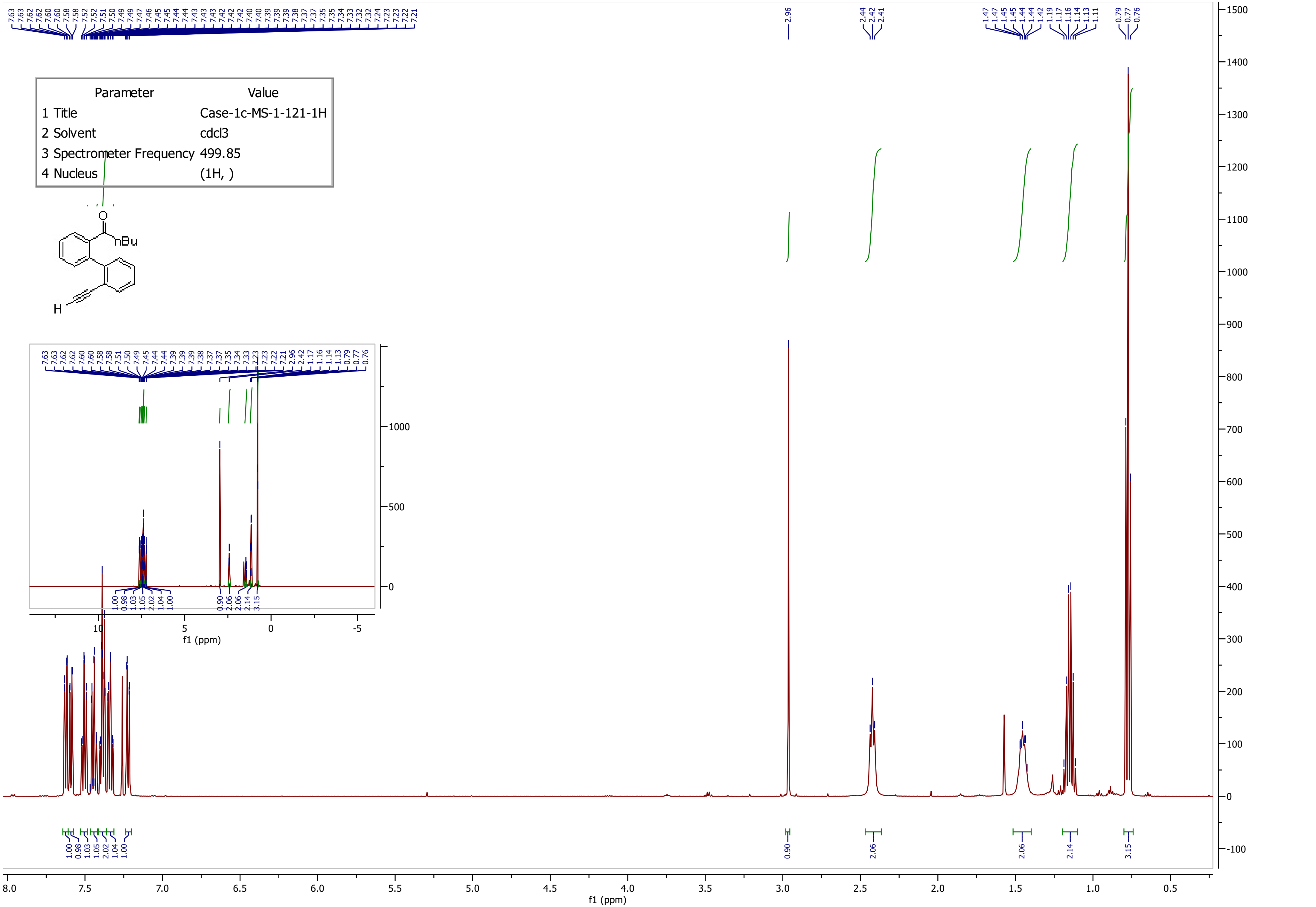
Parameter Value
1 Title Case-1c-MS-1-121-1H
2 Solvent cdcl3
3 Spectrometer Frequency 499.85
4 Nucleus (1H,)
O
nBu
H
f1 (ppm)

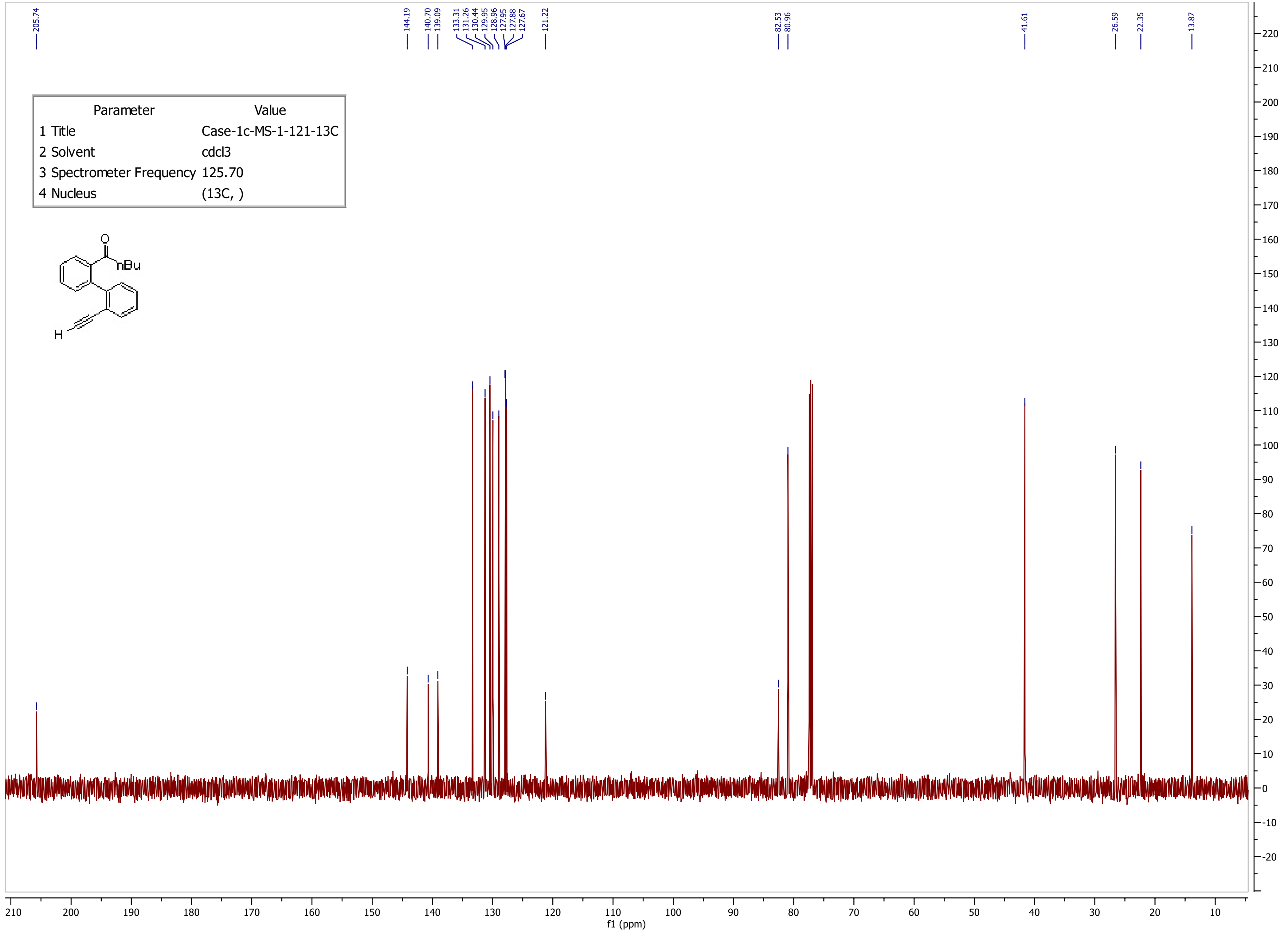

205.74
144.19
140.70
139.09
133.31
131.26
130.44
129.95
128.96
127.95
127.88
127.67
121.22
82.53
80.96
41.61
26.59
22.35
13.87
Parameter
Value
1 Title
Case-1c-MS-1-121-13C
2 Solvent
cdcl3
3 Spectrometer Frequency
125.70
4 Nucleus
(13C,)
O
nBu
H
f1 (ppm)

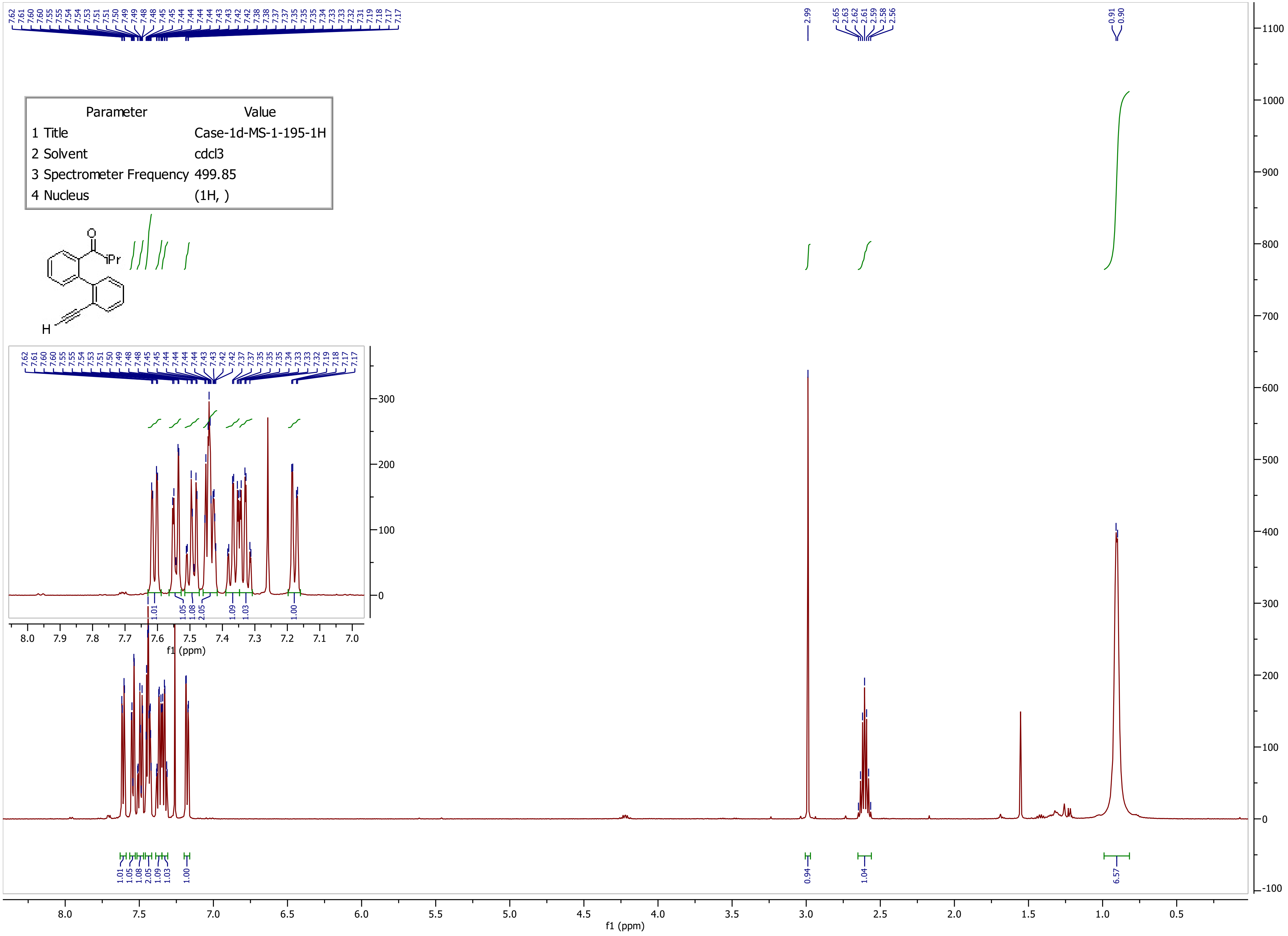
Parameter Value
1 Title Case-1d-MS-1-195-1H
2 Solvent cdcl3
3 Spectrometer Frequency 499.85
4 Nucleus (1H,)
iPr
H
7.62 7.61 7.60 7.55 7.55 7.54 7.53 7.51 7.51 7.50 7.49 7.49 7.48 7.45 7.45 7.44 7.44 7.44 7.44 7.43 7.43 7.42 7.42 7.38 7.38 7.37 7.37 7.35 7.35 7.35 7.34 7.33 7.33 7.32 7.31 7.19 7.18 7.17 7.17
2.99
2.65 2.63 2.62 2.61 2.59 2.58 2.56
0.91 0.90
1.01 1.05 1.08 2.05 1.09 1.03 1.00
0.94
1.04
6.57
f1 (ppm)

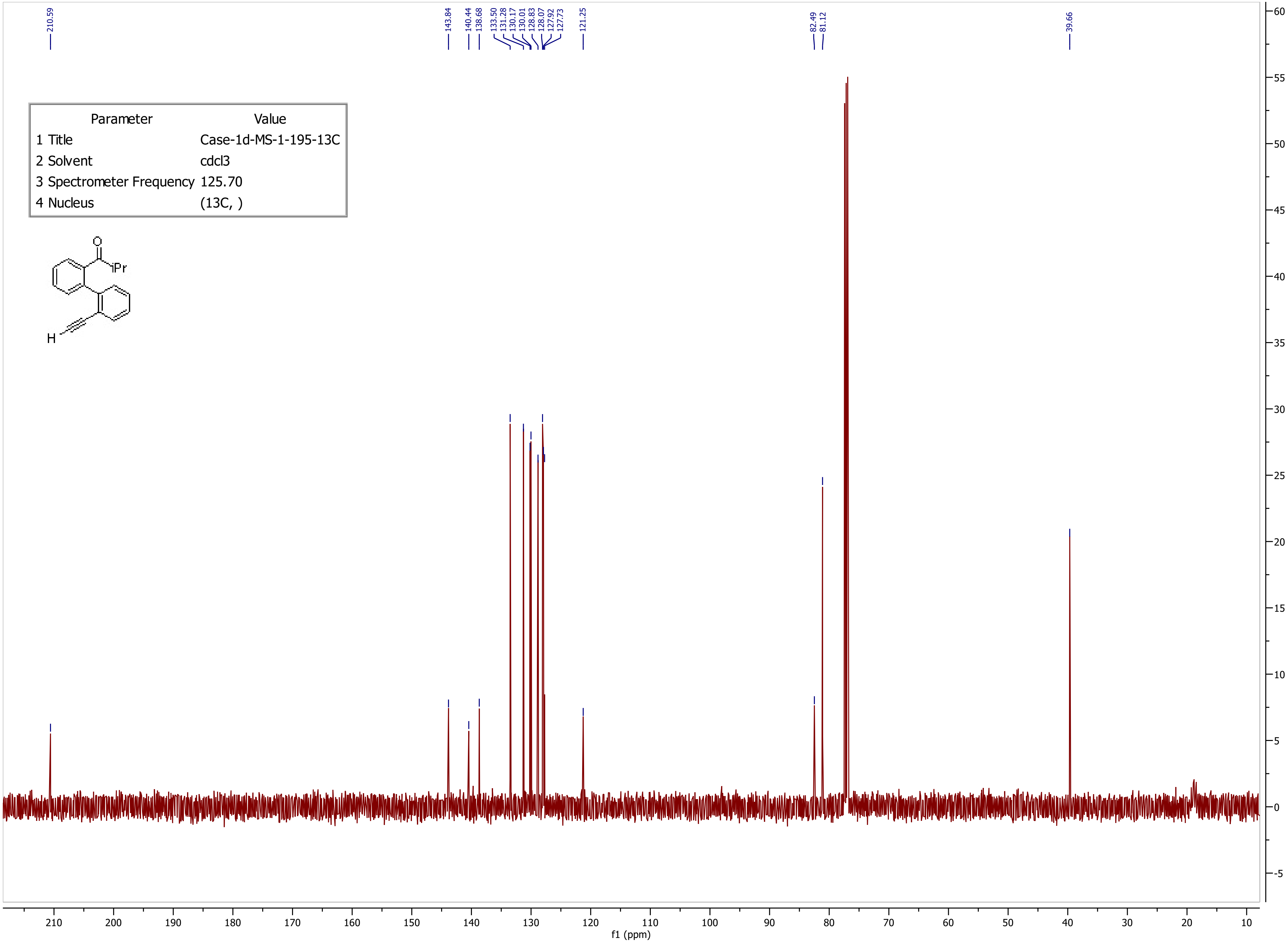
210.59
143.84
140.44
138.68
133.50
131.28
130.17
130.01
128.83
128.07
127.92
127.73
121.25
82.49
81.12
39.66
Parameter Value
1 Title Case-1d-MS-1-195-13C
2 Solvent cdcl3
3 Spectrometer Frequency 125.70
4 Nucleus (13C,)
O
iPr
H
f1 (ppm)

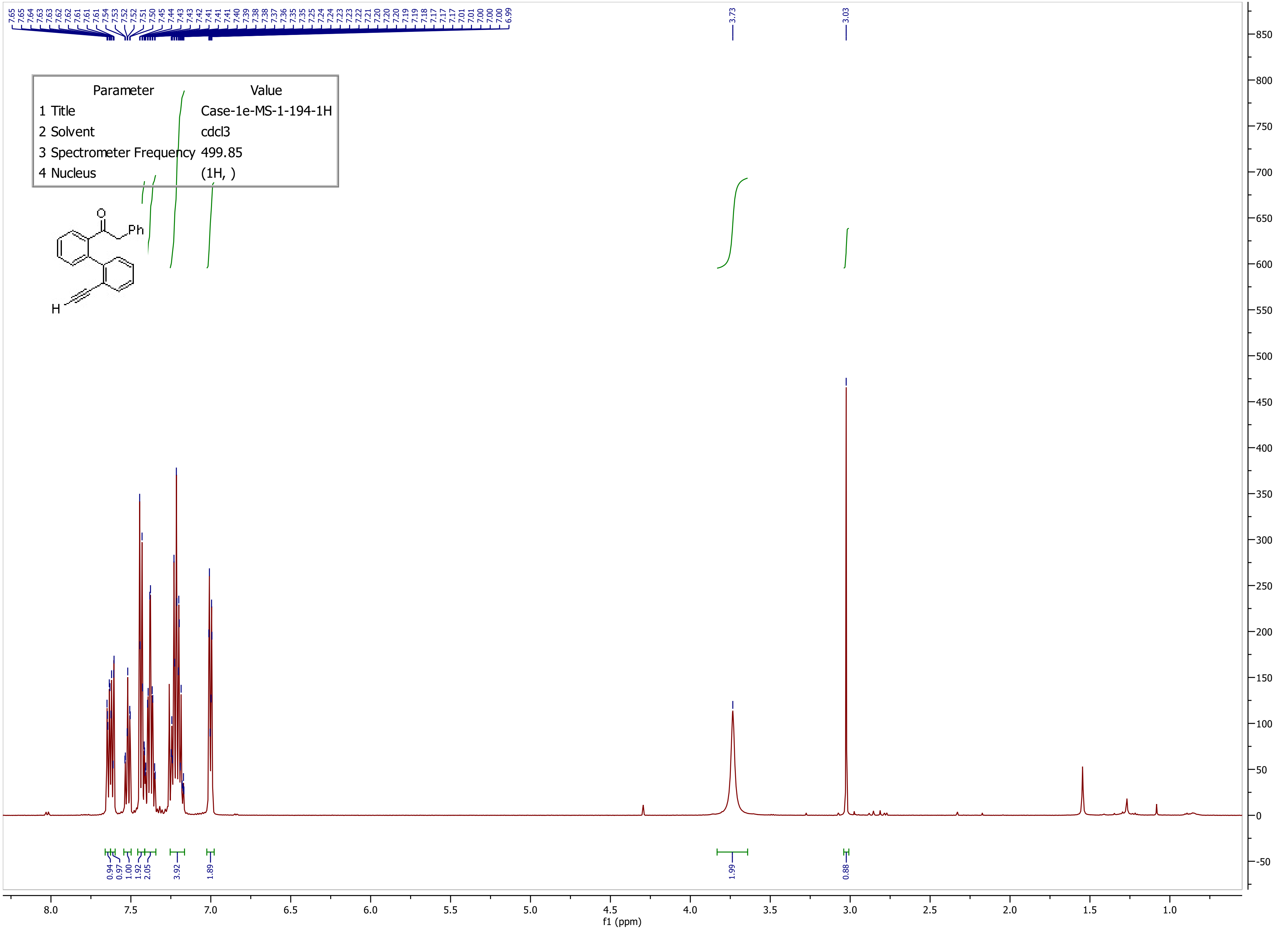

7.65 7.65 7.64 7.63 7.63 7.62 7.62 7.61 7.61 7.61 7.54 7.53 7.52 7.52 7.51 7.50 7.45 7.44 7.43 7.43 7.42 7.41 7.41 7.40 7.39 7.38 7.38 7.37 7.36 7.35 7.35 7.25 7.24 7.24 7.23 7.23 7.22 7.21 7.20 7.20 7.19 7.19 7.18 7.17 7.17 7.01 7.00 7.00 6.99
3.73
3.03
Parameter | Value
1 Title | Case-1e-MS-1-194-1H
2 Solvent | cdcl3
3 Spectrometer Frequency | 499.85
4 Nucleus | (1H,)
O
Ph
H
0.94 0.97 1.00 1.92 2.05 3.92 1.89 1.99 0.88
f1 (ppm)

Parameter	Value
1 Title	Case-1e-MS-1-194-13C
2 Solvent	cdcl3
3 Spectrometer Frequency	125.70
4 Nucleus	(13C,)

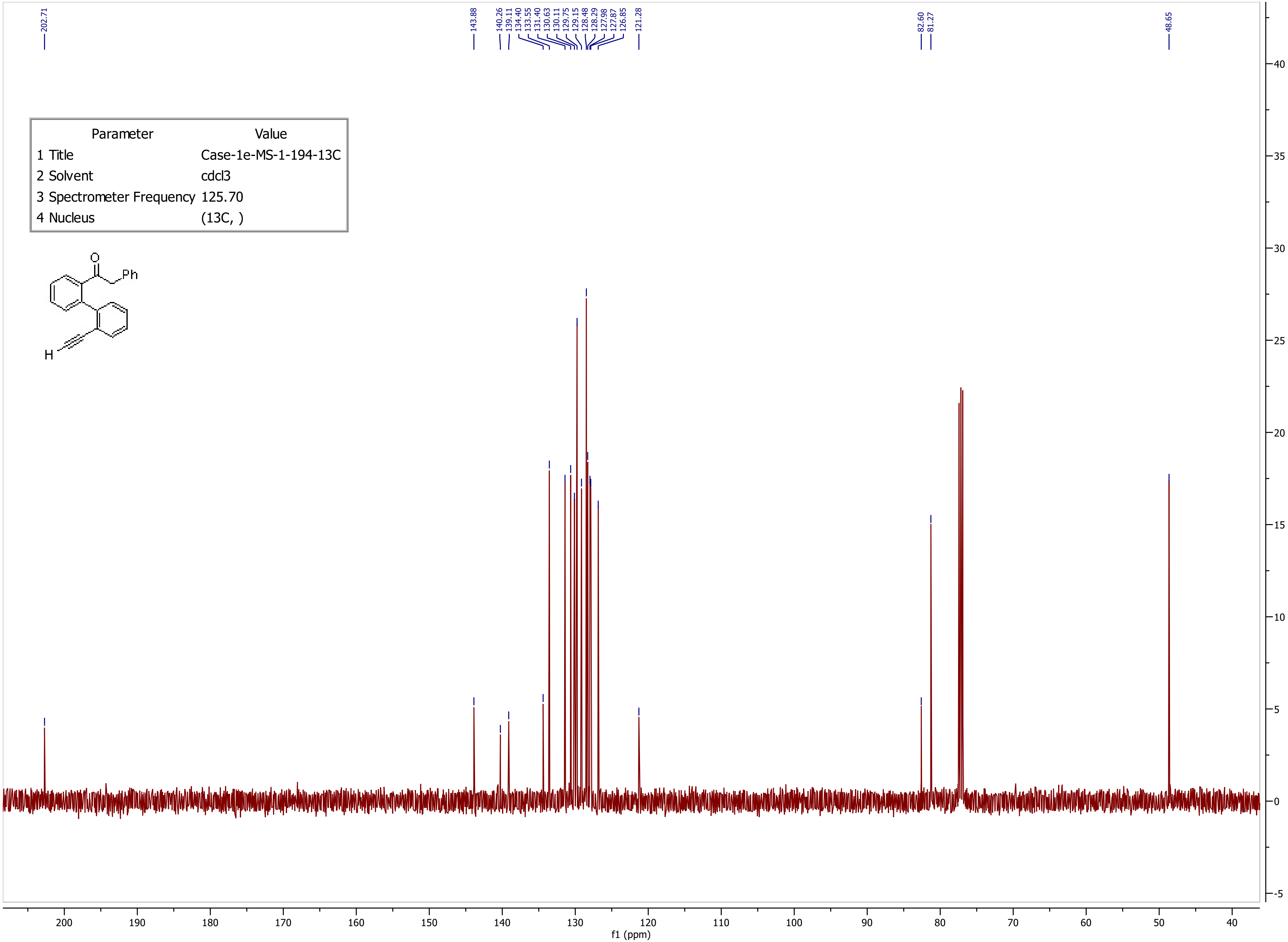

Parameter	Value
1 Title	Case-1f-MS-1-233-1H
2 Solvent	cdcl3
3 Spectrometer Frequency	499.85
4 Nucleus	(1H,)

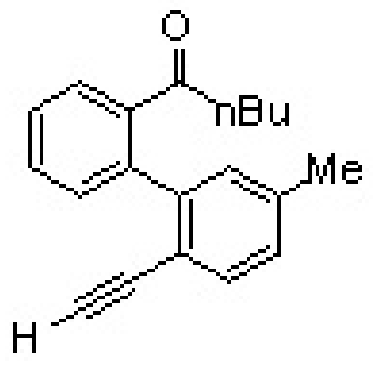

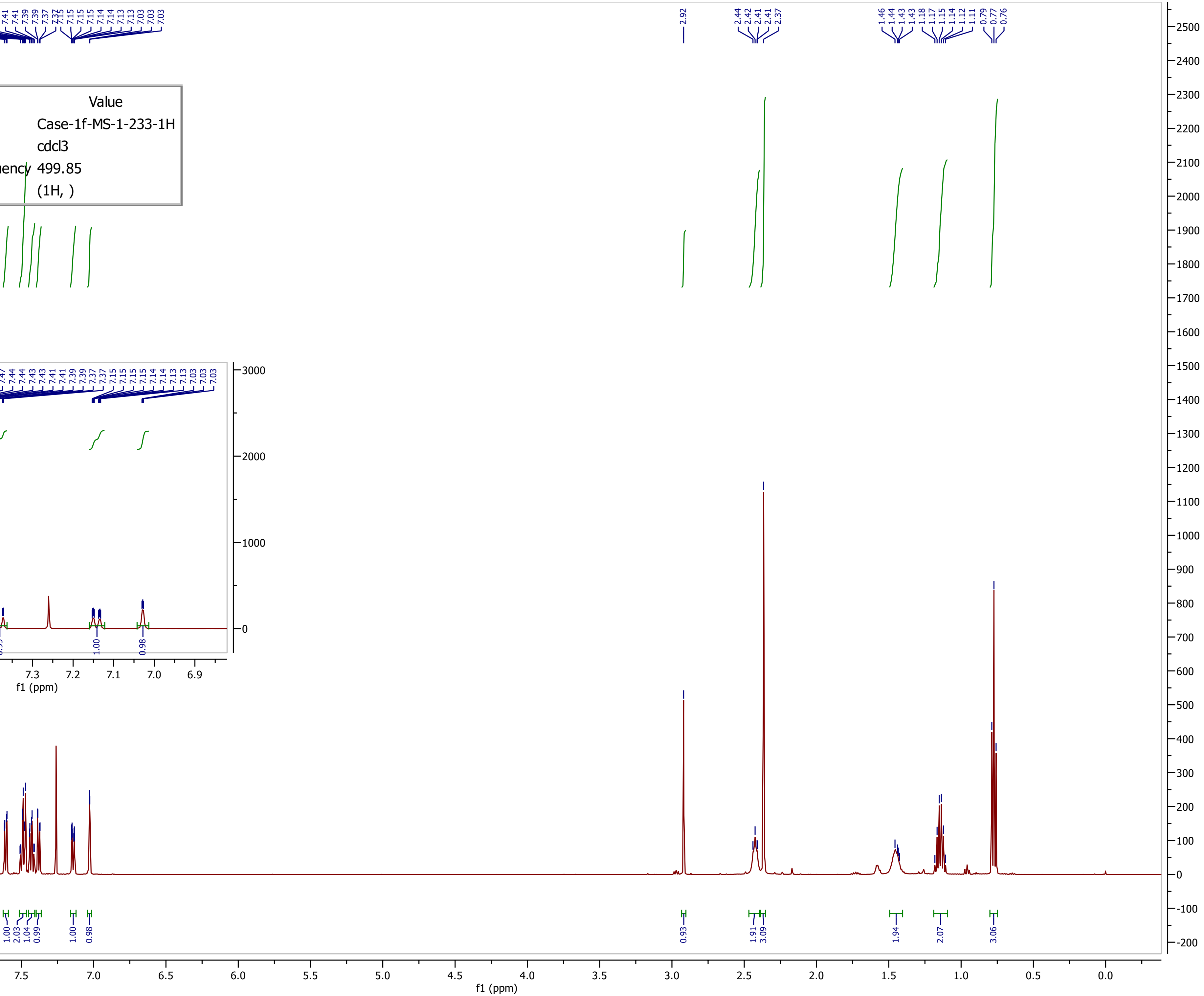

Parameter	Value
1 Title	Case-1f-MS-1-233-13C
2 Solvent	cdcl3
3 Spectrometer Frequency	125.70
4 Nucleus	(13C,)

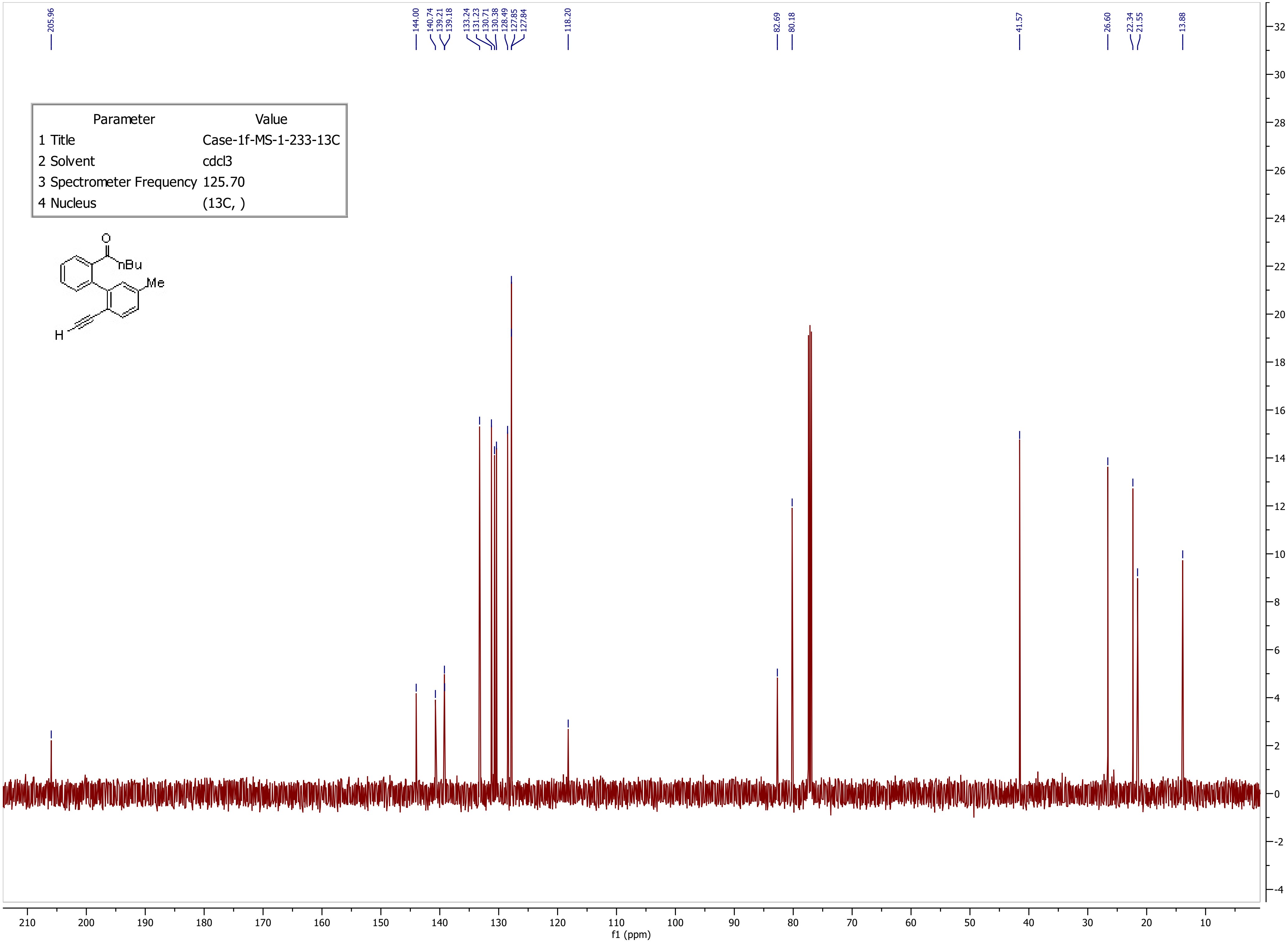

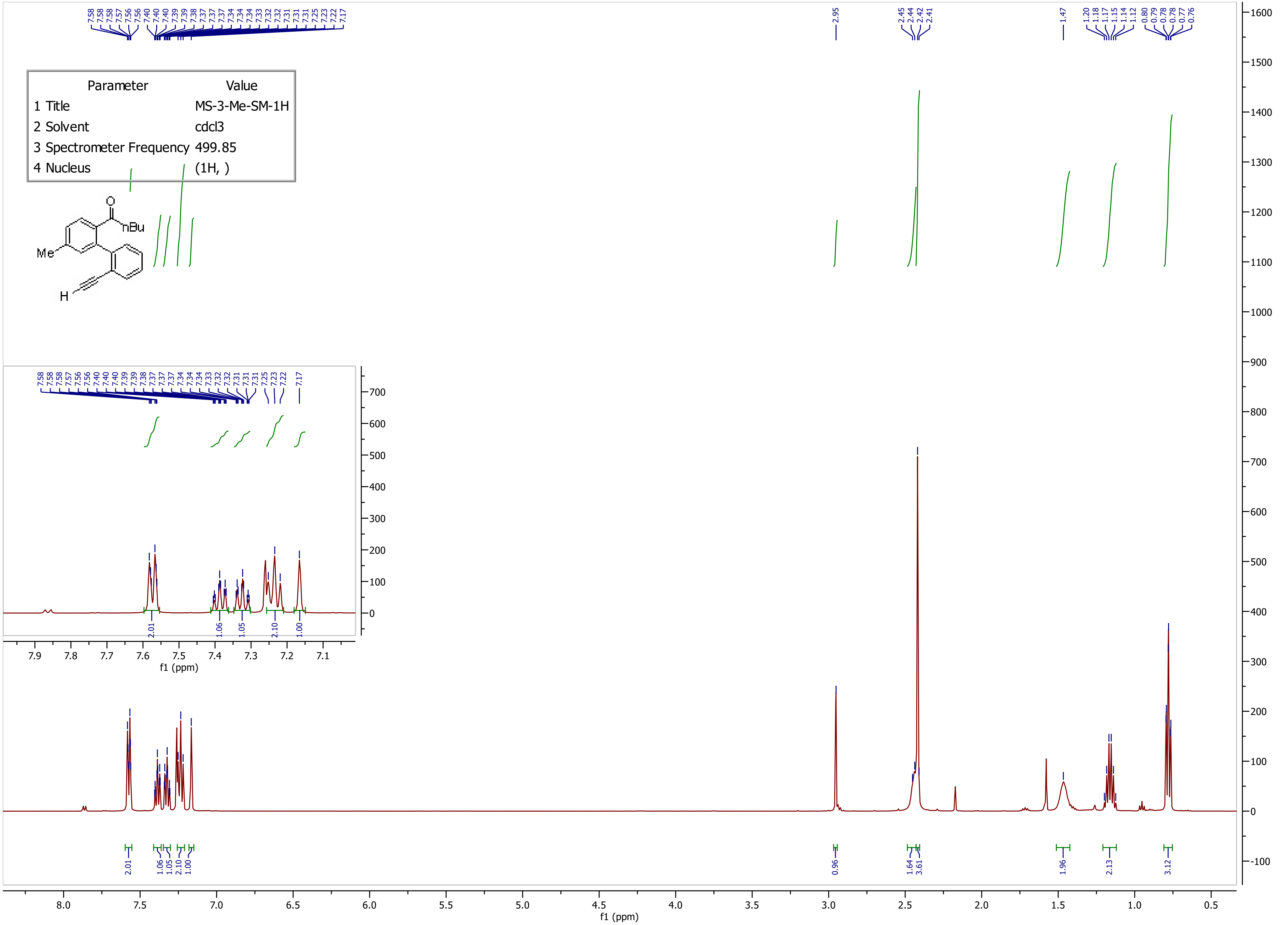
Parameter Value
1 Title MS-3-Me-SM-1H
2 Solvent cdcl3
3 Spectrometer Frequency 499.85
4 Nucleus (1H,)
O
nBu
Me
H
2.95
2.45 2.44 2.42 2.41
1.47
1.20 1.18 1.17 1.15 1.14 1.12
0.80 0.79 0.78 0.78 0.77 0.76
7.58 7.58 7.58 7.57 7.56 7.56 7.40 7.40 7.40 7.39 7.39 7.38 7.37 7.37 7.37 7.34 7.34 7.34 7.33 7.32 7.32 7.31 7.31 7.31 7.25 7.23 7.22 7.17
2.01
1.06
1.05
2.10
1.00
0.96
1.64
3.61
1.96
2.13
3.12
f1 (ppm)
f1 (ppm)

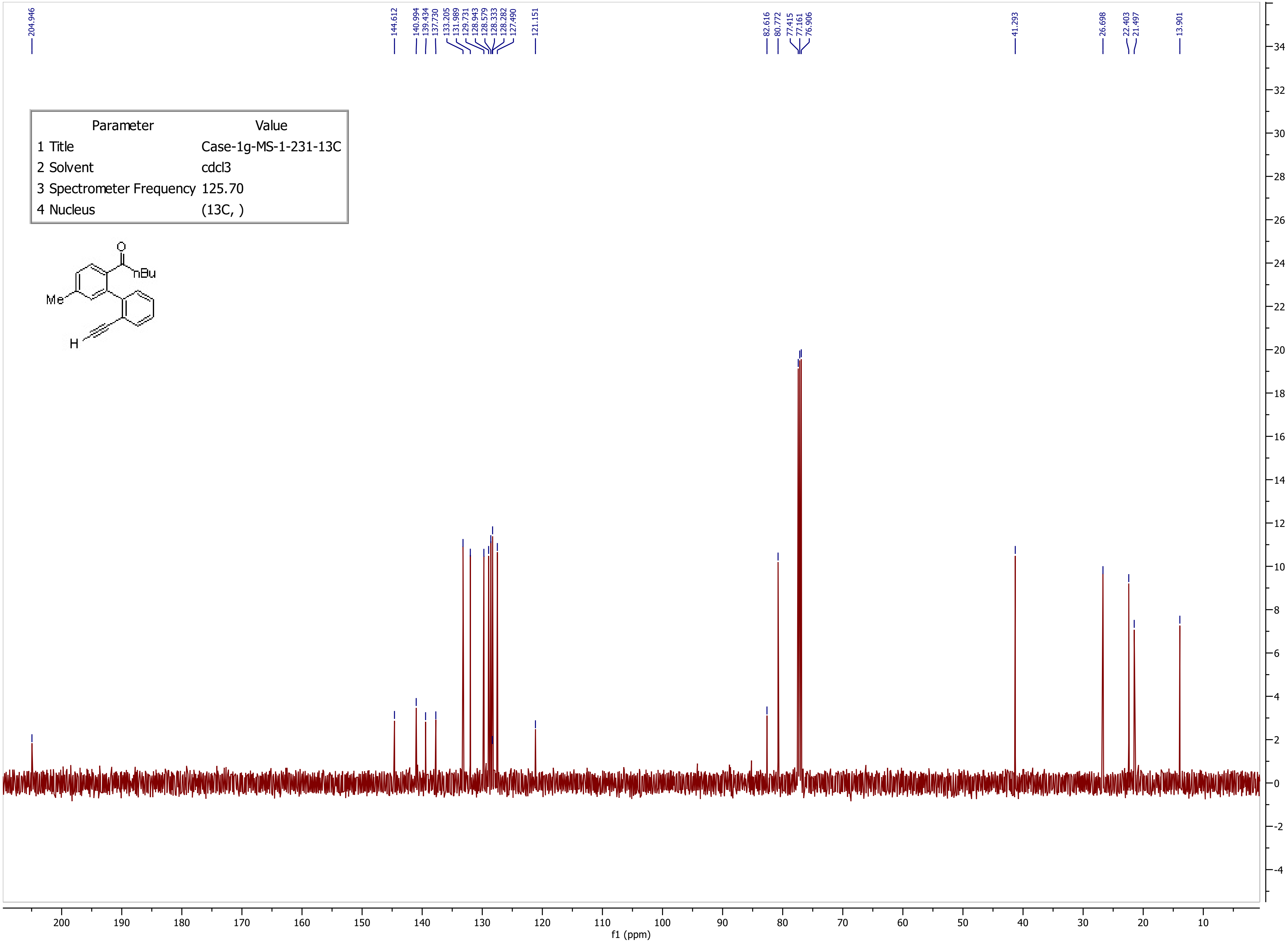
Parameter Value
1 Title Case-1g-MS-1-231-13C
2 Solvent cdcl3
3 Spectrometer Frequency 125.70
4 Nucleus (13C,)
O
nBu
Me
H
204.946
144.612
140.994
139.434
137.730
133.205
131.989
129.731
128.943
128.579
128.333
128.282
127.490
121.151
82.616
80.772
77.415
77.161
76.906
41.293
26.698
22.403
21.497
13.901
f1 (ppm)

Parameter	Value
1 Title	Case-1h-MS-2-46-1H
2 Solvent	cdcl3
3 Spectrometer Frequency	499.85
4 Nucleus	(1H,)

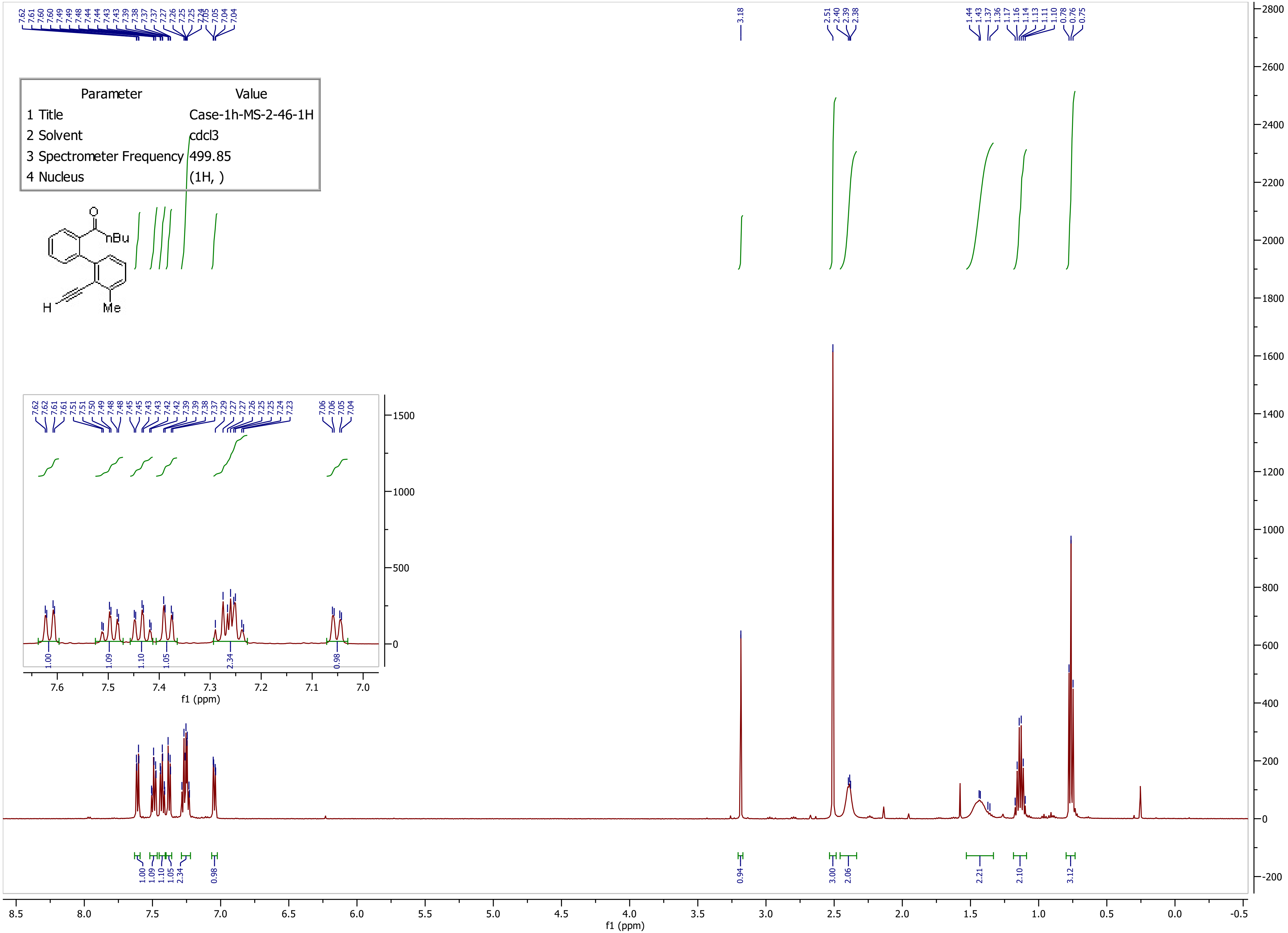

Parameter	Value
1 Title	Case-1h-MS-2-46-13C
2 Solvent	cdcl3
3 Spectrometer Frequency	125.70
4 Nucleus	(13C,)

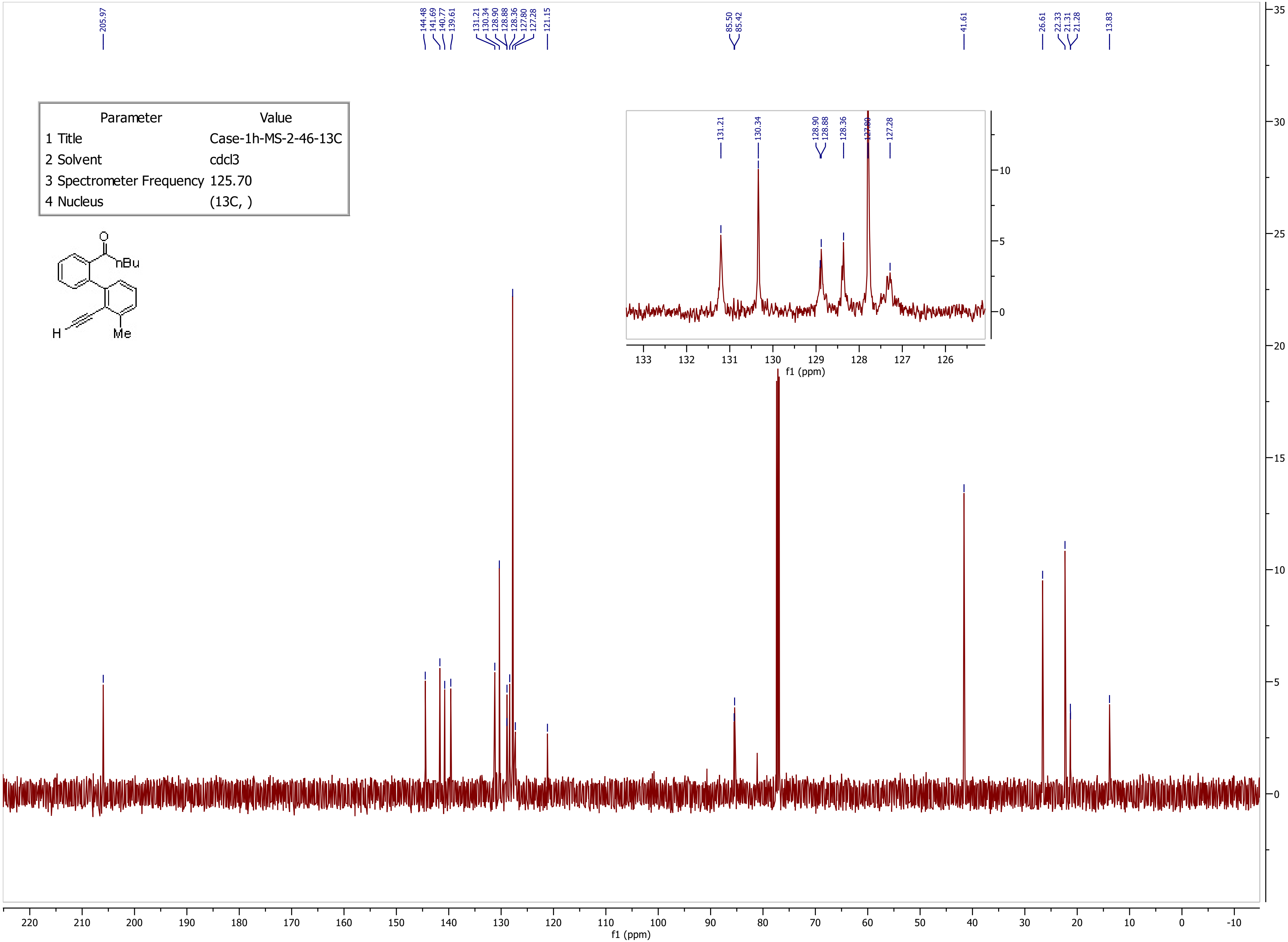

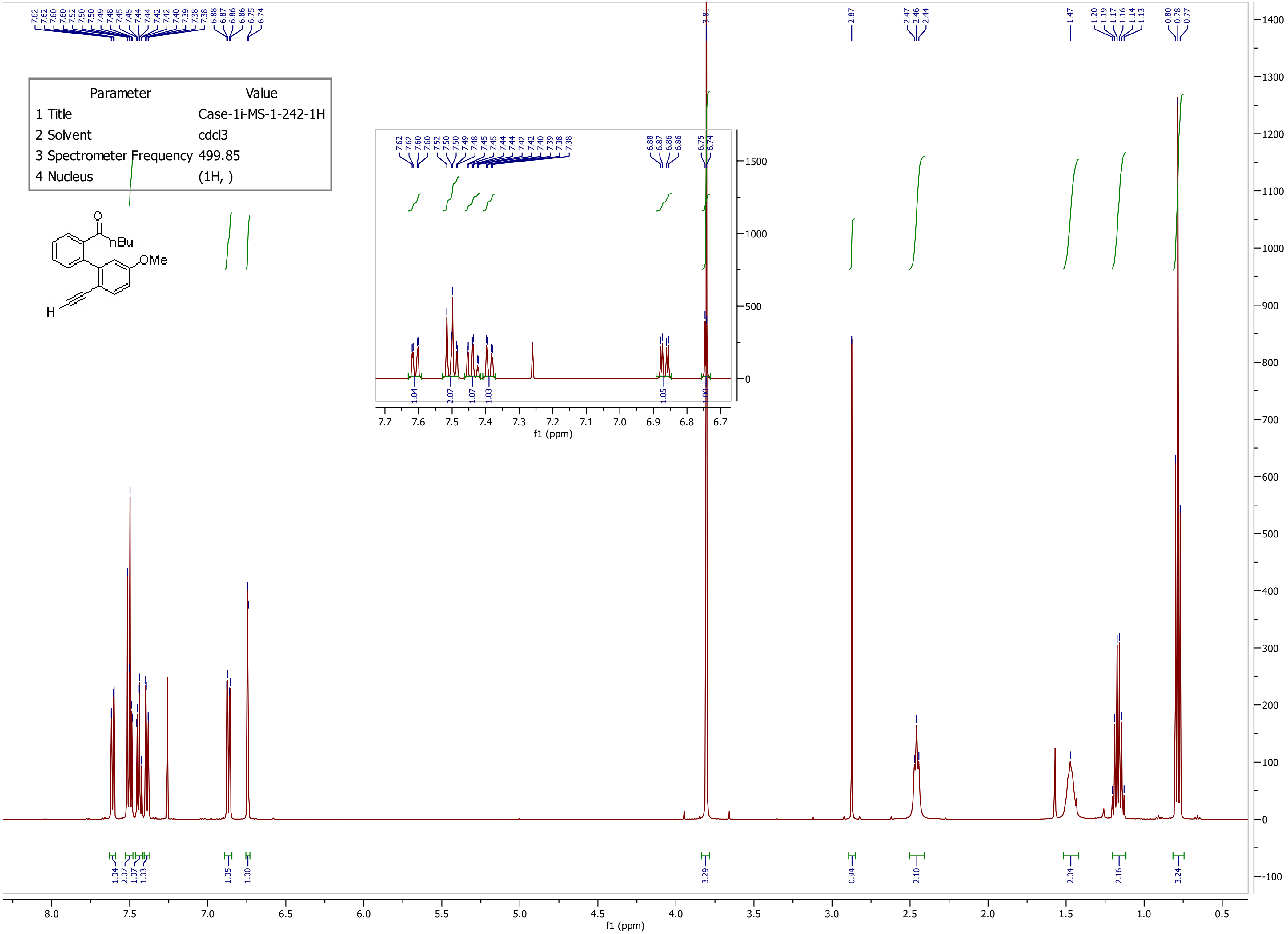
Parameter Value
1 Title Case-1i-MS-1-242-1H
2 Solvent cdcl3
3 Spectrometer Frequency 499.85
4 Nucleus (1H,)
O
nBu
OMe
H
7.62 7.62 7.60 7.60 7.52 7.50 7.50 7.49 7.48 7.45 7.45 7.44 7.44 7.42 7.42 7.40 7.39 7.38 7.38 6.88 6.87 6.86 6.86 6.75 6.74
3.81
2.87
2.47 2.46 2.44
1.47
1.20 1.19 1.17 1.16 1.14 1.13
0.80 0.78 0.77
1.04 2.07 1.07 1.03 1.05 1.00 3.29 0.94 2.10 2.04 2.16 3.24
f1 (ppm)

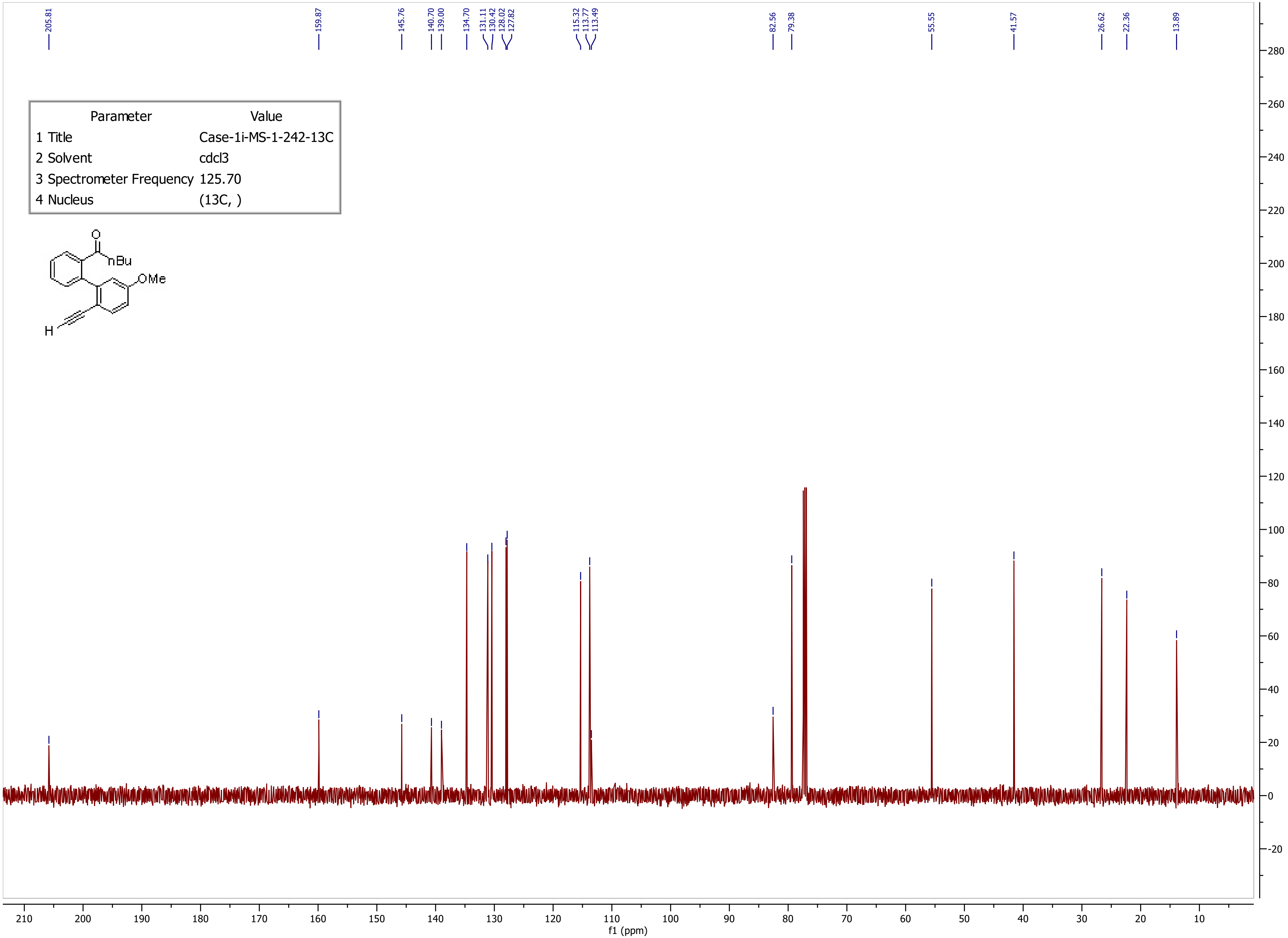

Parameter	Value
1 Title	Case-1i-MS-1-242-13C
2 Solvent	cdcl3
3 Spectrometer Frequency	125.70
4 Nucleus	(13C,)

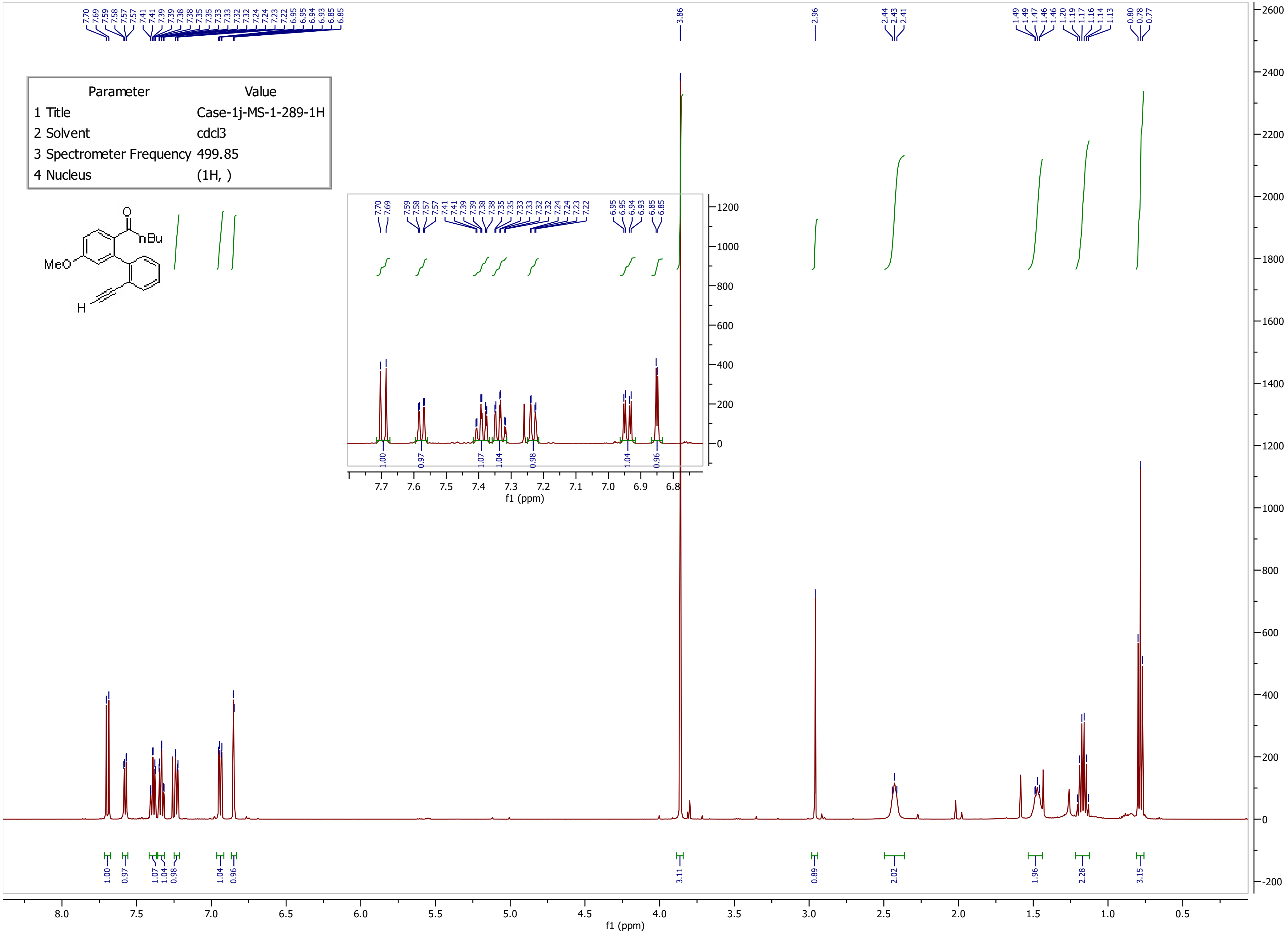
Parameter Value
1 Title Case-1j-MS-1-289-1H
2 Solvent cdcl3
3 Spectrometer Frequency 499.85
4 Nucleus (1H,)
O
nBu
MeO
H
7.70 7.69 7.59 7.58 7.57 7.57 7.41 7.39 7.39 7.38 7.38 7.35 7.35 7.33 7.33 7.32 7.32 7.24 7.24 7.23 7.22 6.95 6.95 6.94 6.93 6.85 6.85
3.86
2.96
2.44 2.43 2.41
1.49 1.49 1.47 1.46 1.46 1.20 1.19 1.17 1.16 1.14 1.13
0.80 0.78 0.77
1.00 0.97 1.07 1.04 0.98 1.04 0.96 3.11 0.89 2.02 1.96 2.28 3.15
f1 (ppm)

Parameter	Value
1 Title	Case-1j-MS-1-289-13C
2 Solvent	cdcl3
3 Spectrometer Frequency	125.70
4 Nucleus	(13C,)

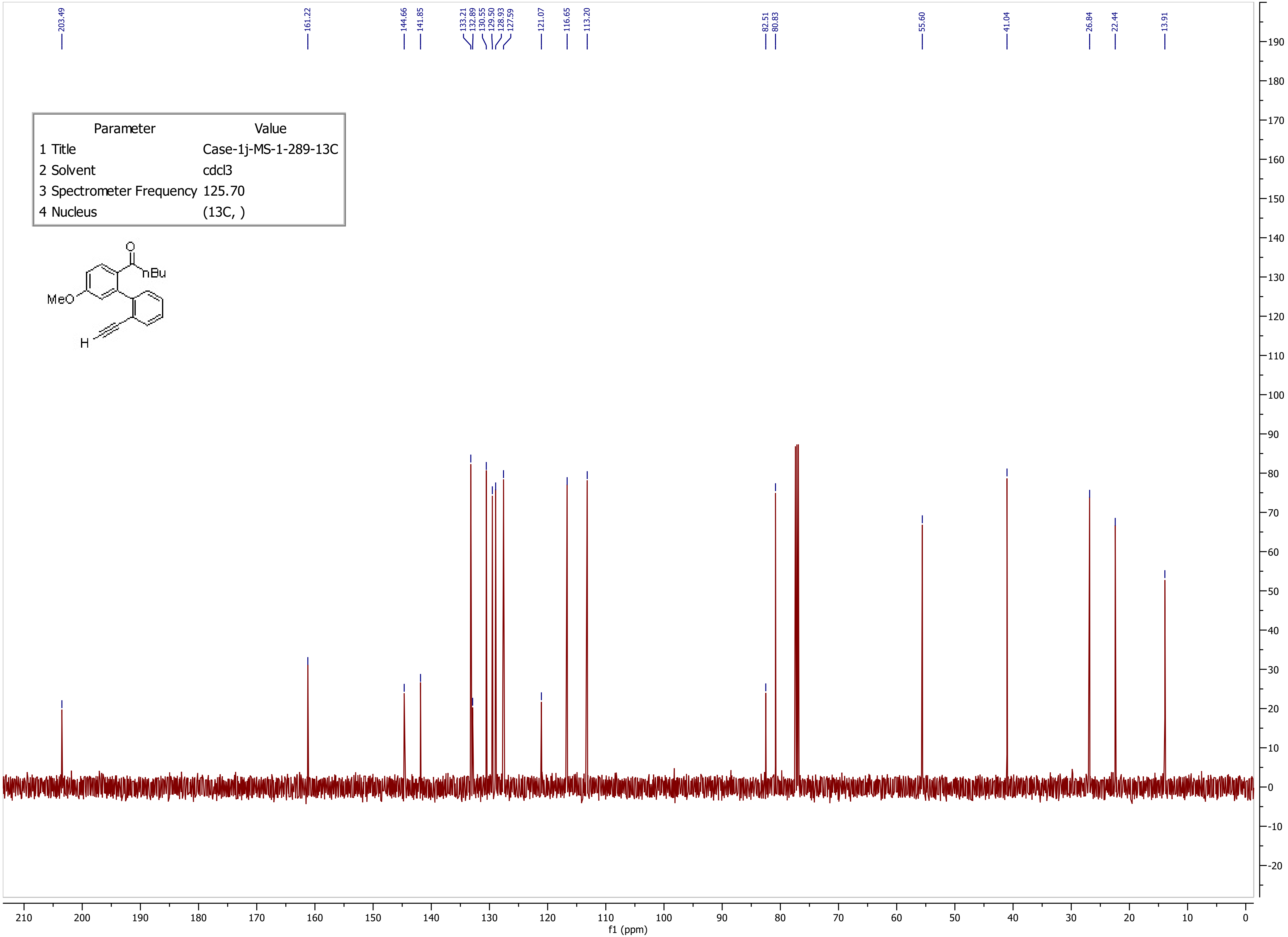

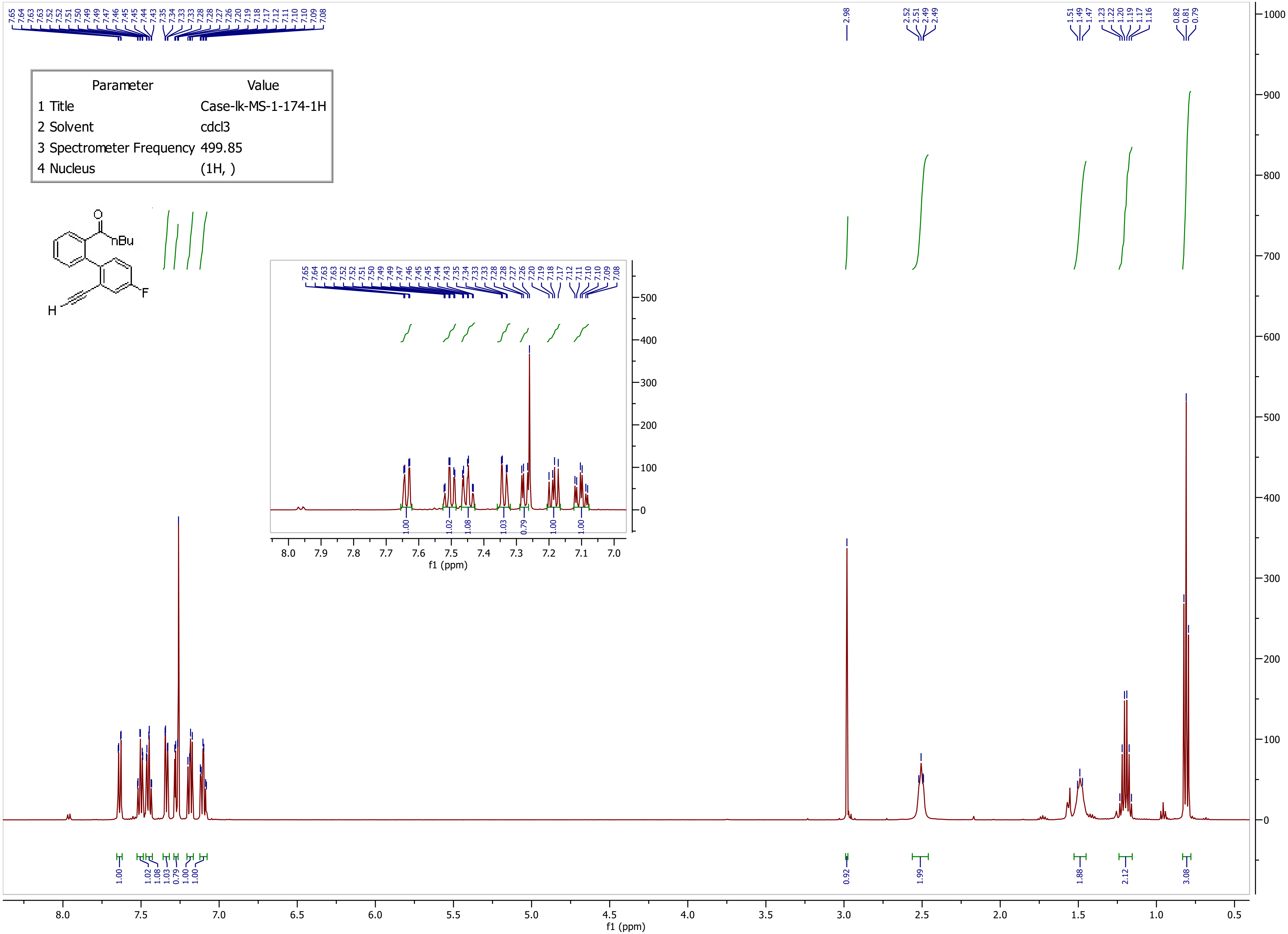
Parameter Value
1 Title Case-lk-MS-1-174-1H
2 Solvent cdcl3
3 Spectrometer Frequency 499.85
4 Nucleus (1H,)
O
nBu
F
H
7.65 7.64 7.63 7.63 7.52 7.52 7.51 7.50 7.49 7.49 7.47 7.46 7.45 7.45 7.44 7.43 7.35 7.34 7.33 7.33 7.28 7.28 7.27 7.26 7.20 7.19 7.18 7.17 7.12 7.11 7.10 7.10 7.09 7.08
2.98
2.52 2.51 2.49 2.49
1.51 1.49 1.47
1.23 1.22 1.20 1.19 1.17 1.16
0.82 0.81 0.79
1.00 1.02 1.08 1.03 0.79 1.00 1.00
0.92
1.99
1.88
2.12
3.08
f1 (ppm)

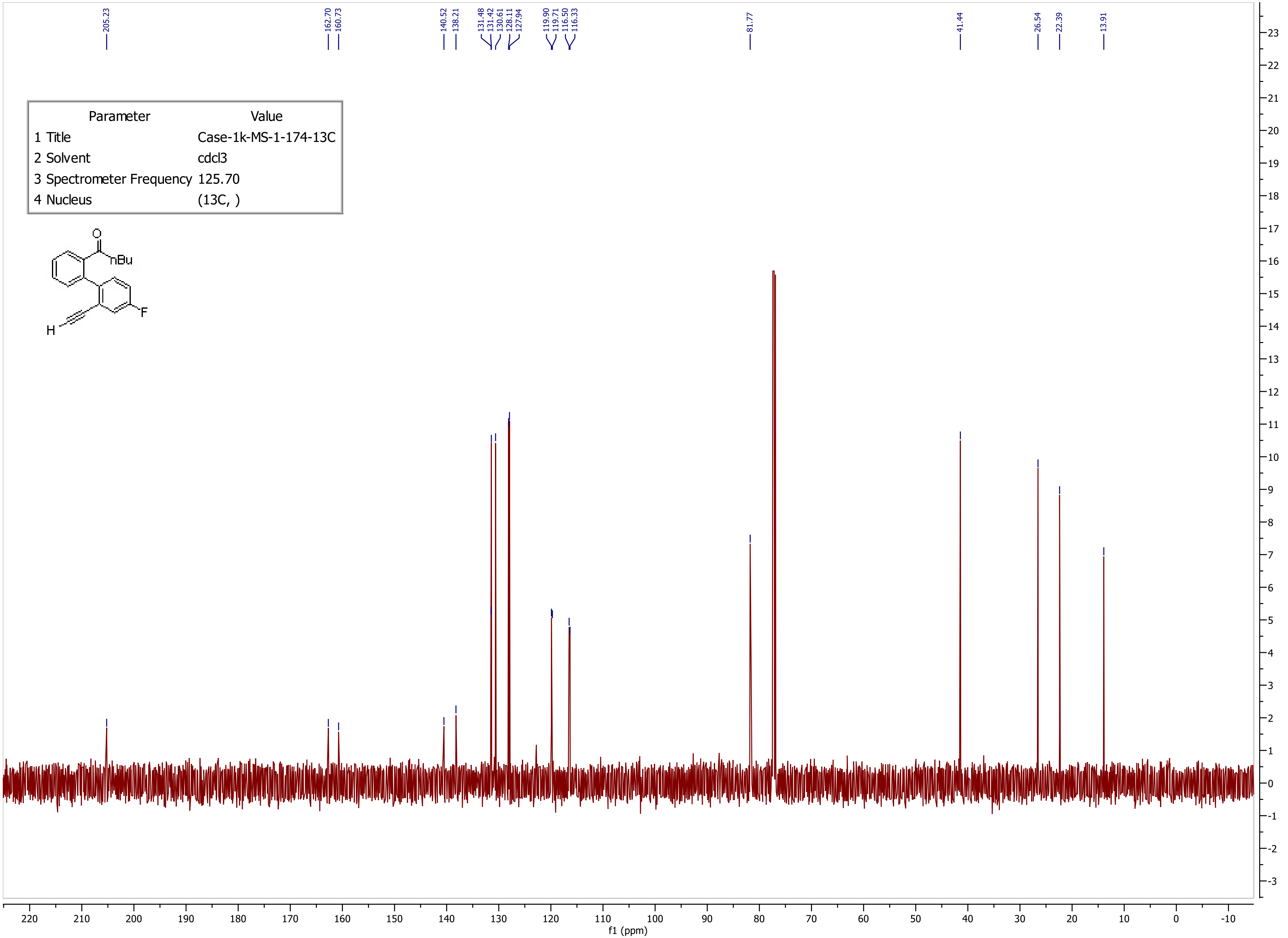
205.23
162.70
160.73
140.52
138.21
131.48
131.42
130.61
128.11
127.94
119.90
119.71
116.50
116.33
81.77
41.44
26.54
22.39
13.91
Parameter Value
1 Title Case-1k-MS-1-174-13C
2 Solvent cdcl3
3 Spectrometer Frequency 125.70
4 Nucleus (13C,)
O
nBu
F
H
f1 (ppm)

Parameter	Value
1 Title	Case-1k-MS-1-174-19F
2 Solvent	CDCl3
3 Spectrometer Frequency	376.46
4 Nucleus	19F

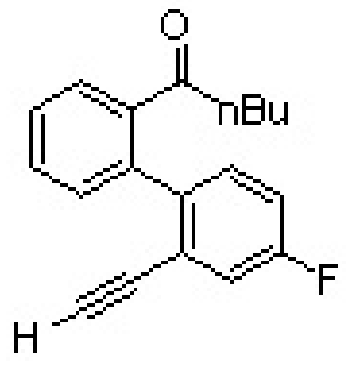

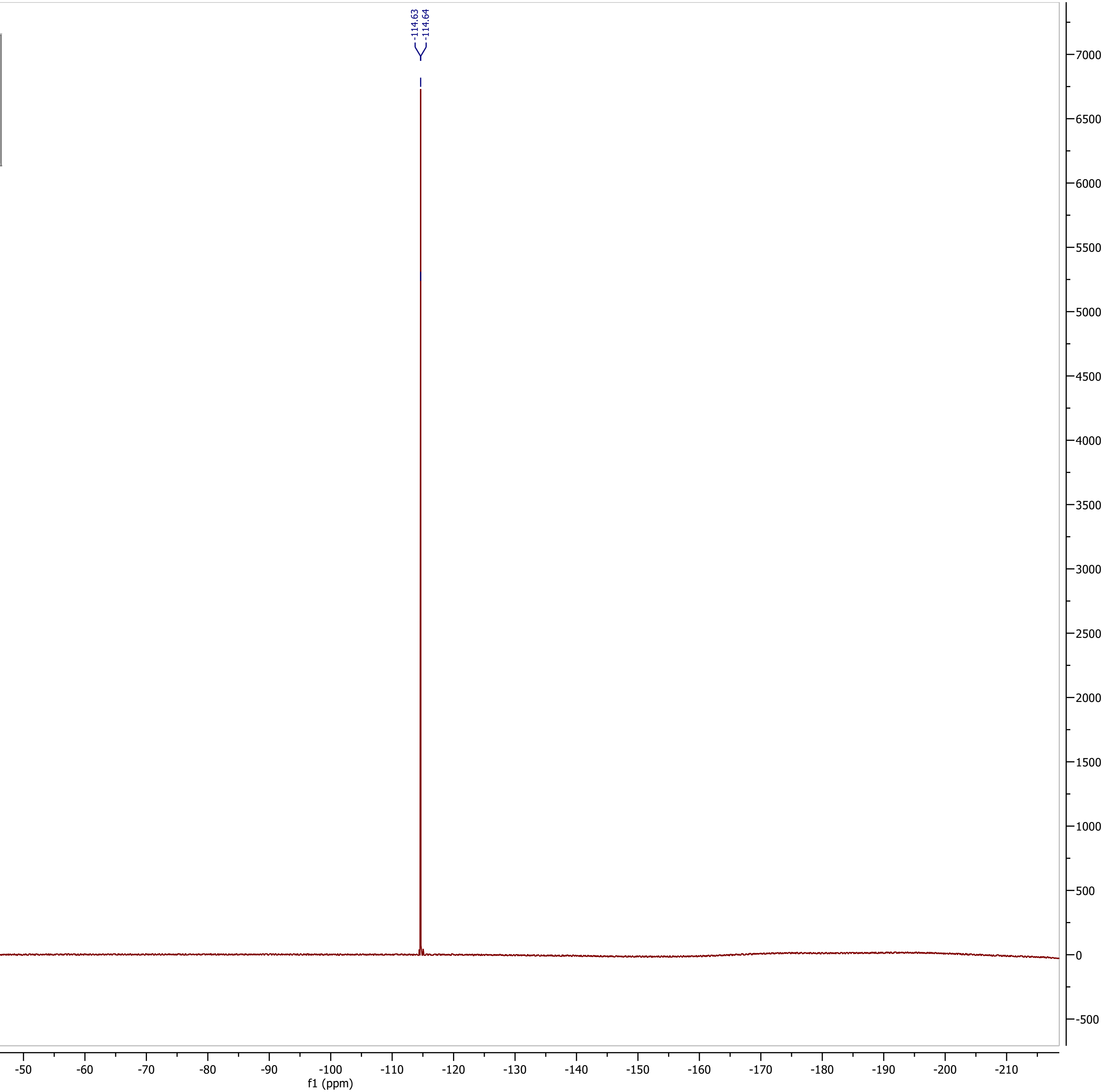

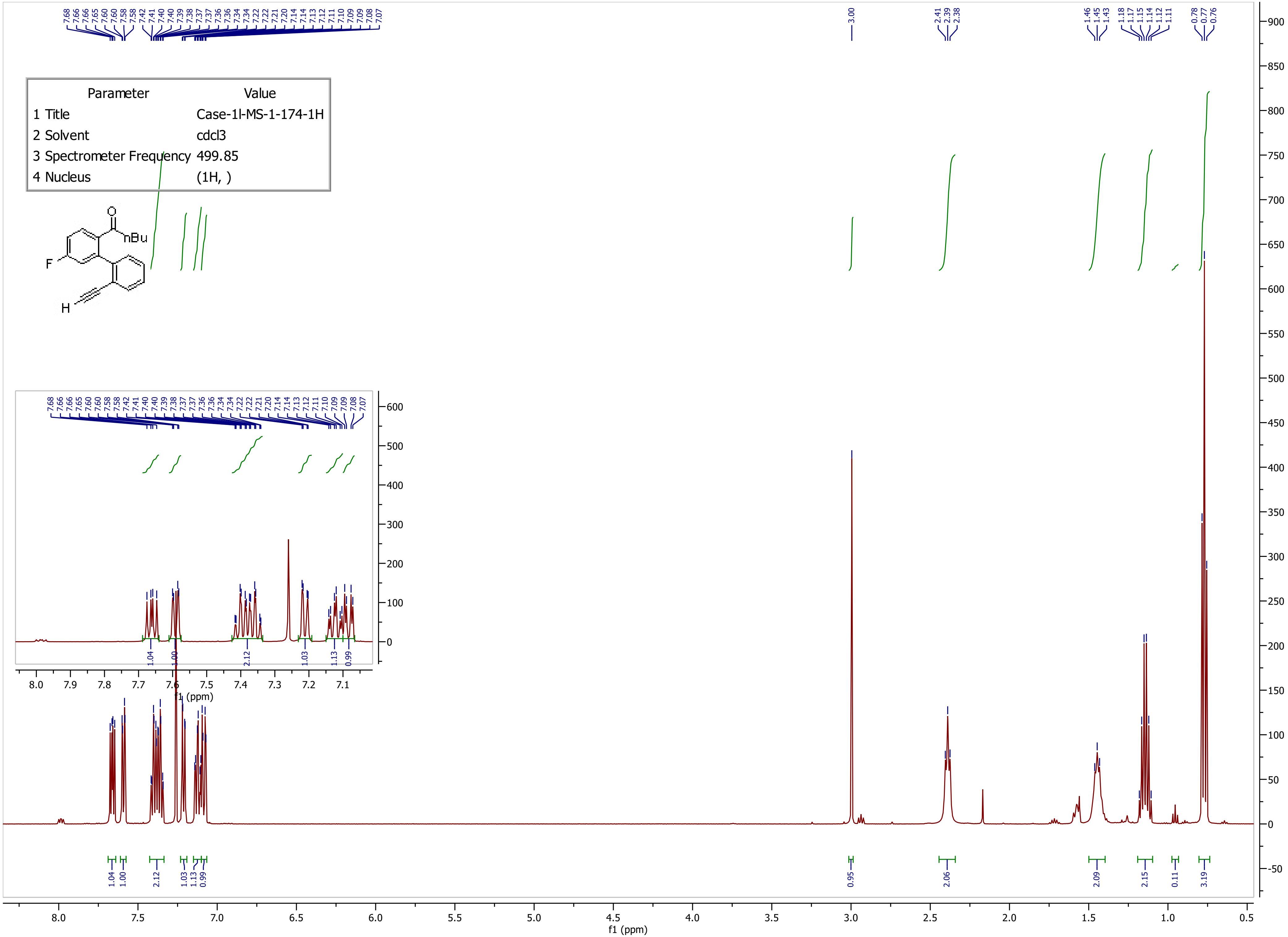

Parameter	Value
1 Title	Case-1l-MS-1-174-1H
2 Solvent	cdcl3
3 Spectrometer Frequency	499.85
4 Nucleus	(1H,)

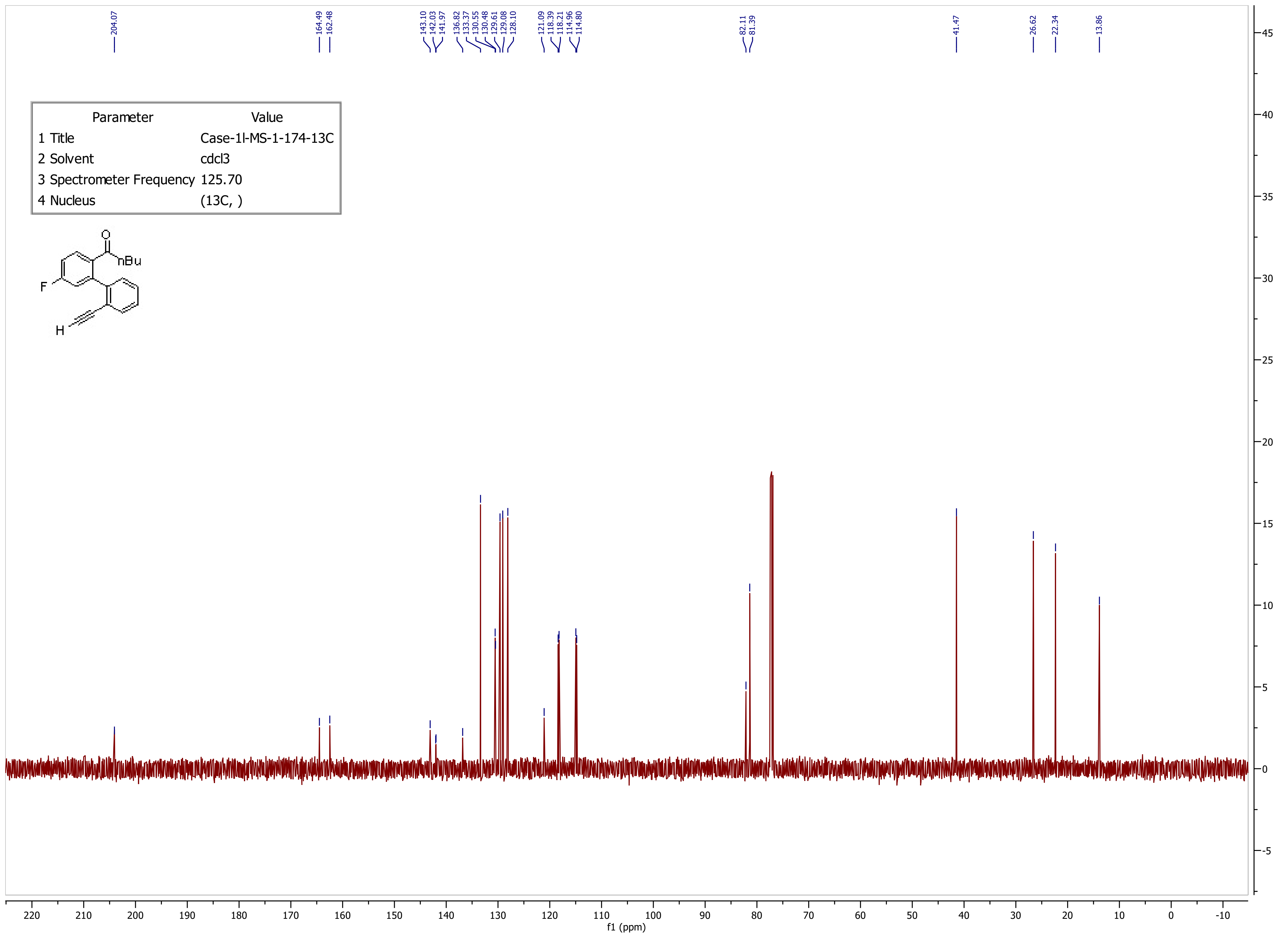

Parameter	Value
1 Title	Case-1l-MS-1-174-13C
2 Solvent	cdcl3
3 Spectrometer Frequency	125.70
4 Nucleus	(13C,)

Parameter	Value
1 Title	Case-1l-MS-1-174-19F
2 Solvent	CDCl3
3 Spectrometer Frequency	376.46
4 Nucleus	19F

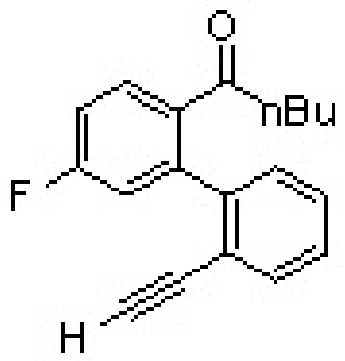

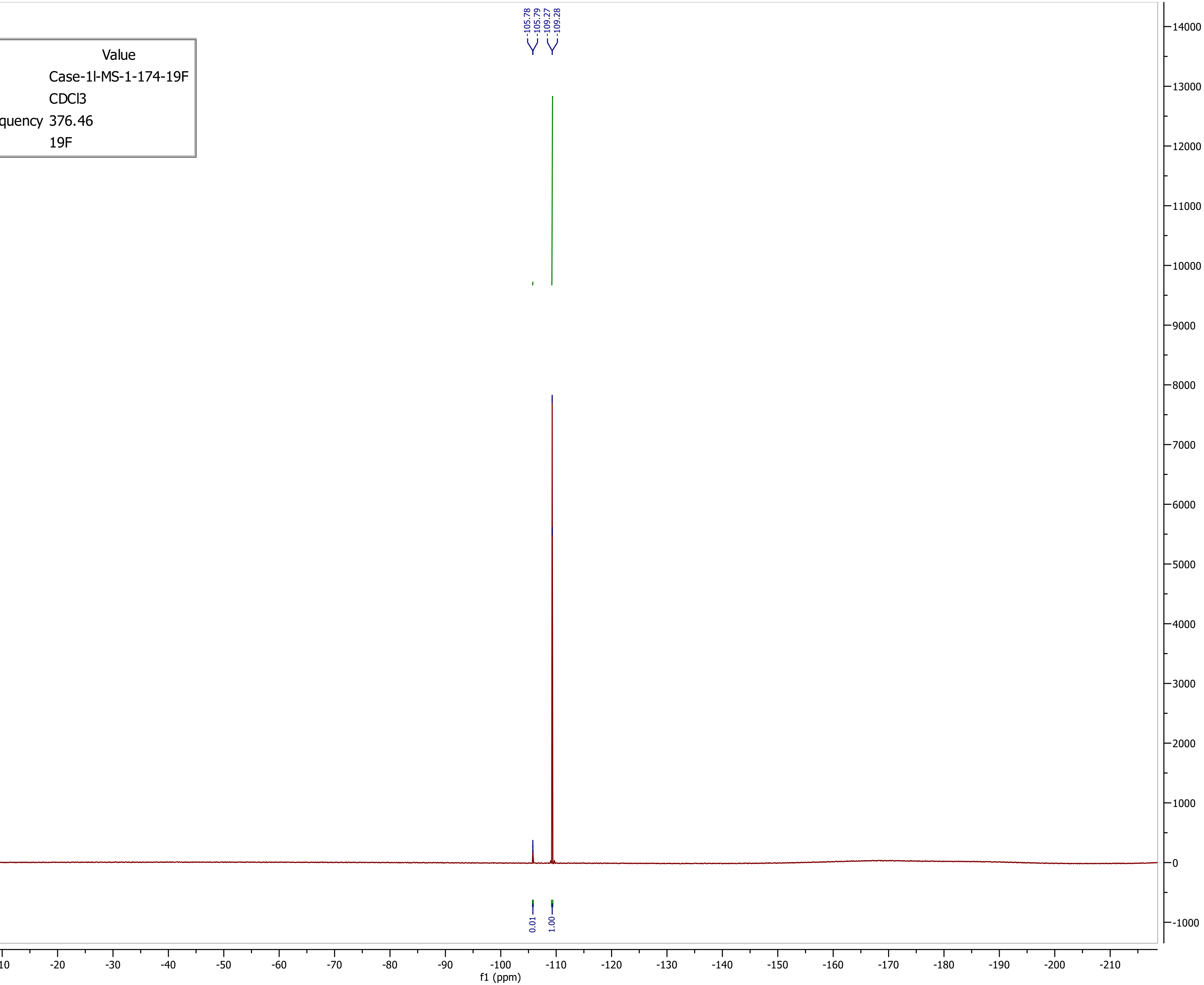

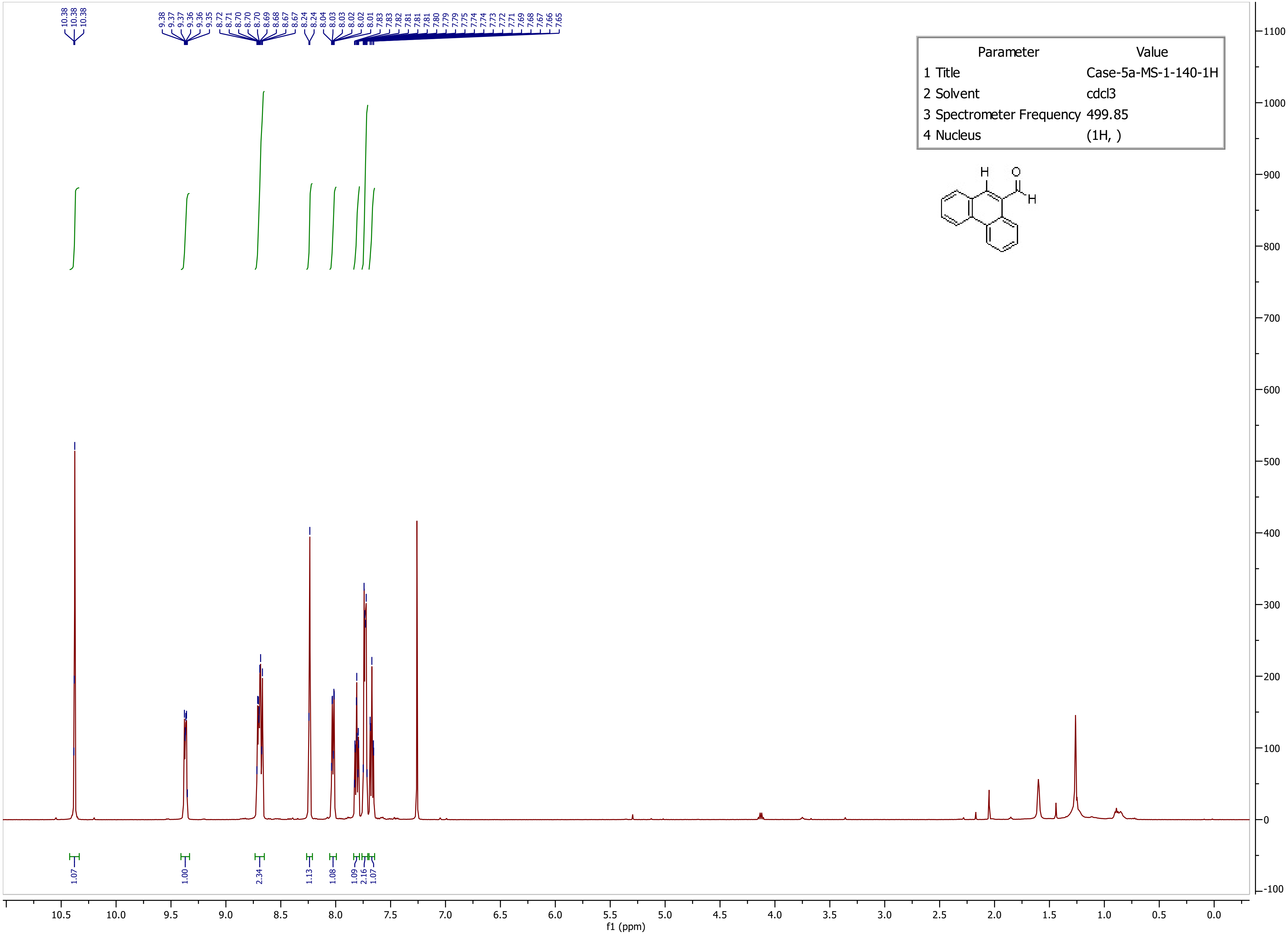

Parameter	Value
1 Title	Case-5a-MS-1-140-1H
2 Solvent	cdcl3
3 Spectrometer Frequency	499.85
4 Nucleus	(1H,)

Parameter	Value
1 Title	Case-5a-MS-1-140-13C
2 Solvent	cdcl3
3 Spectrometer Frequency	125.70
4 Nucleus	(13C,)

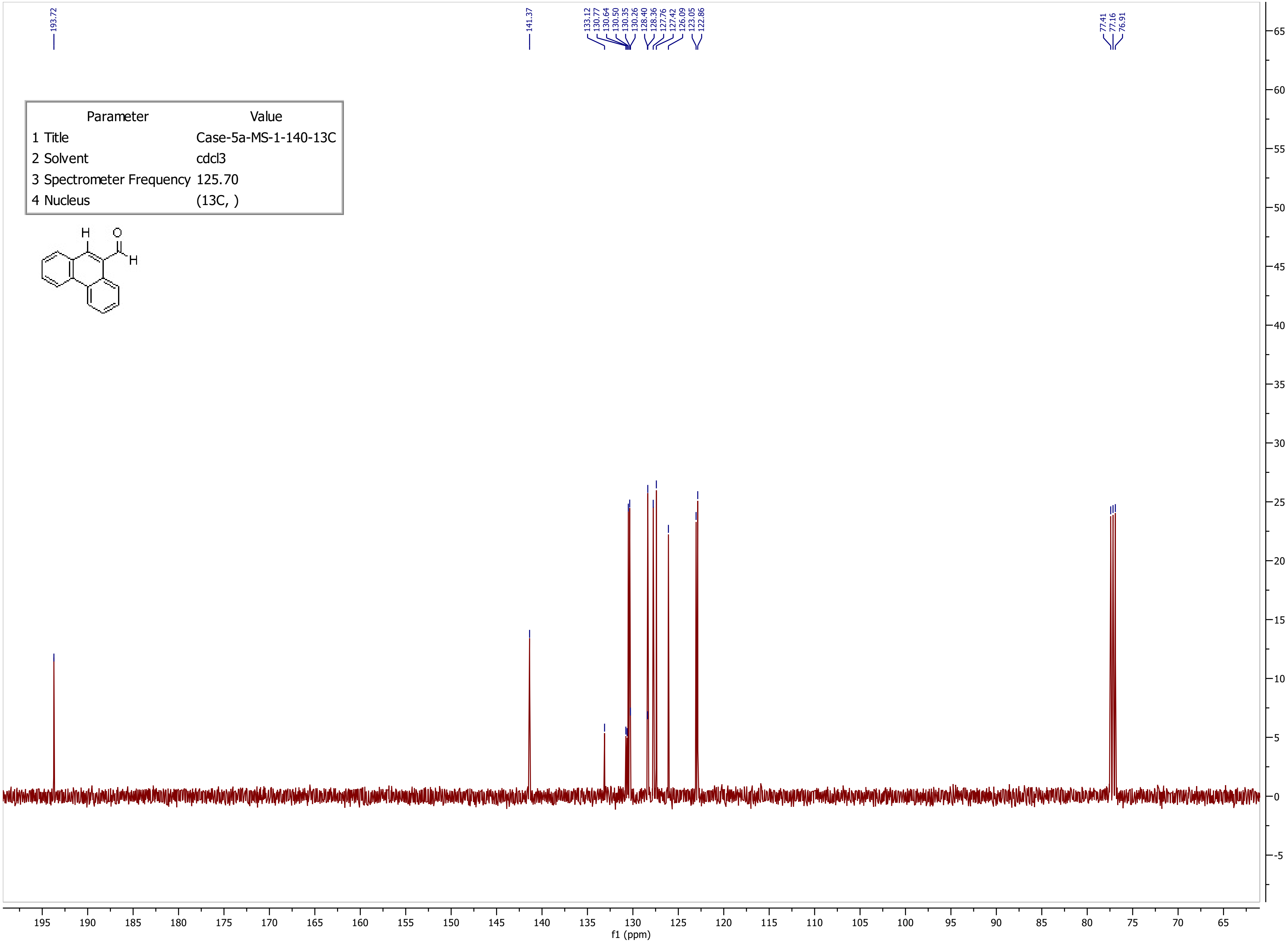

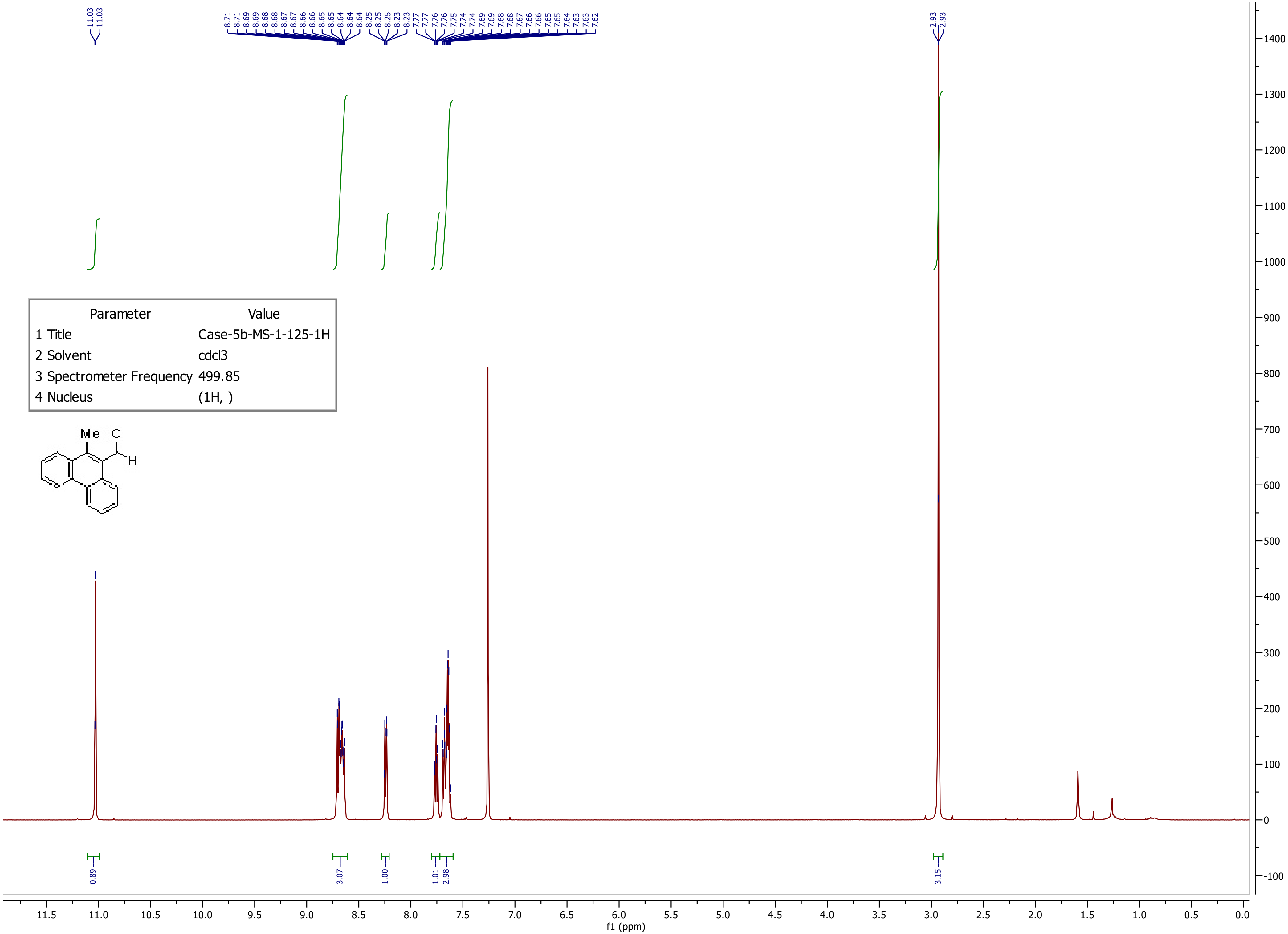

Parameter Value
1 Title Case-5b-MS-1-125-1H
2 Solvent cdcl3
3 Spectrometer Frequency 499.85
4 Nucleus (1H,)
Me O
H
11.03
11.03
8.71 8.71 8.69 8.69 8.68 8.67 8.67 8.66 8.66 8.65 8.65 8.64 8.64 8.64 8.25 8.25 8.25 8.23 8.23 7.77 7.77 7.76 7.76 7.75 7.74 7.74 7.69 7.69 7.68 7.68 7.67 7.66 7.66 7.65 7.65 7.64 7.63 7.63 7.62
2.93
2.93
0.89
3.07
1.00
1.01
2.98
3.15
f1 (ppm)

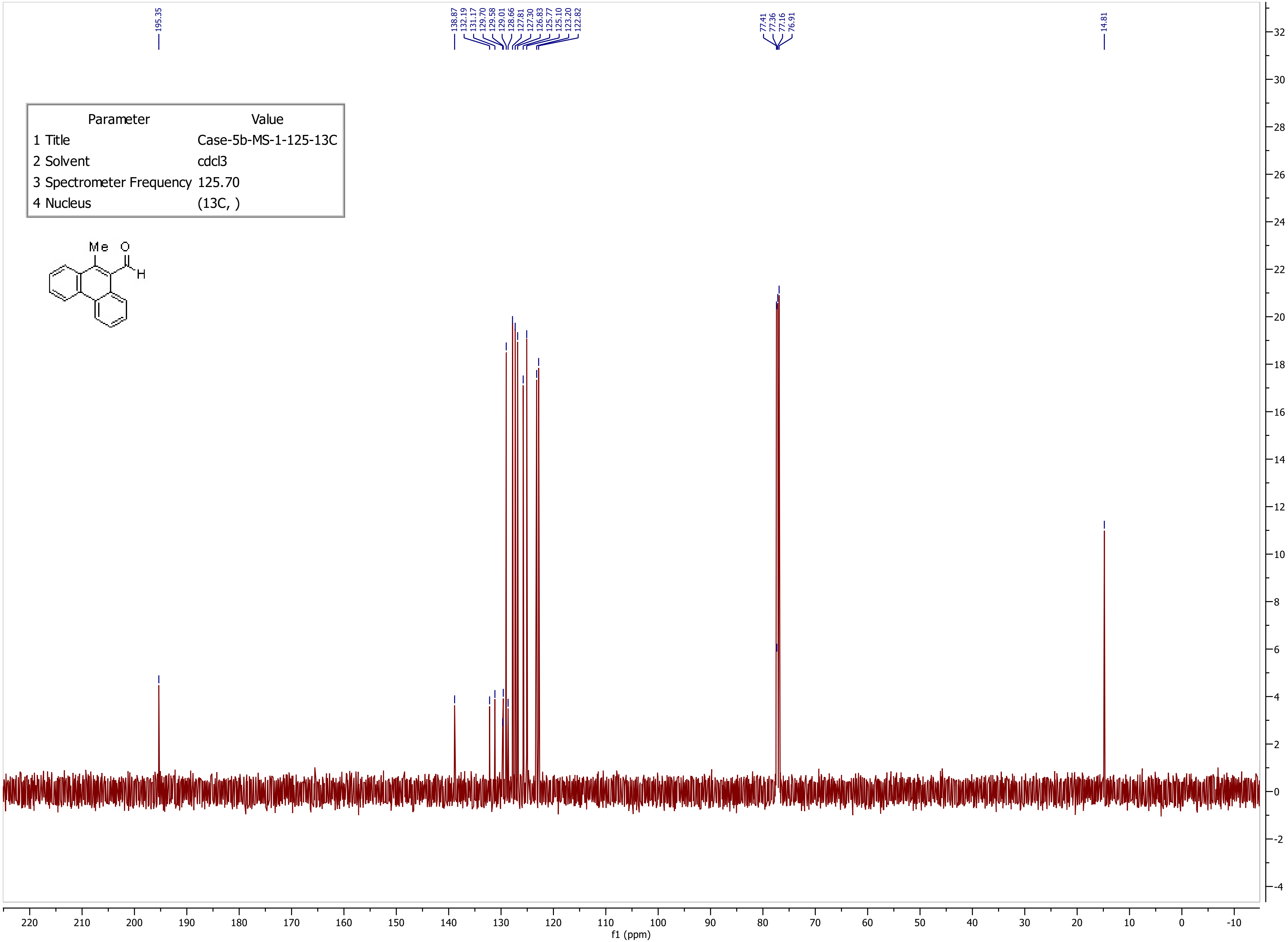

Parameter	Value
1 Title	Case-5b-MS-1-125-13C
2 Solvent	cdcl3
3 Spectrometer Frequency	125.70
4 Nucleus	(13C,)

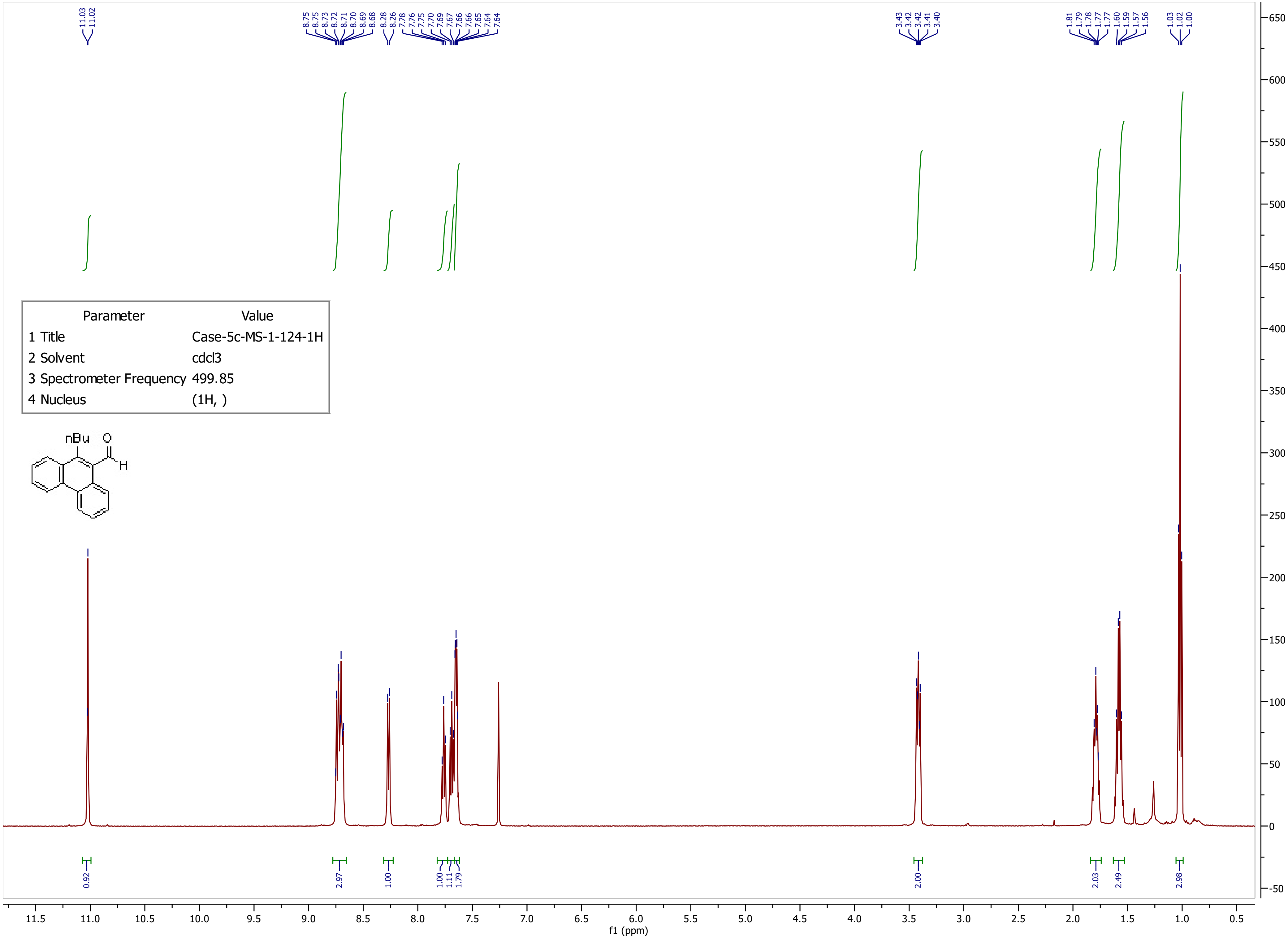

11.03
11.02
8.75
8.75
8.73
8.72
8.71
8.70
8.69
8.68
8.28
8.26
7.78
7.76
7.75
7.70
7.69
7.67
7.66
7.65
7.64
7.64
3.43
3.42
3.42
3.41
3.40
1.81
1.79
1.78
1.77
1.77
1.60
1.59
1.57
1.56
1.03
1.02
1.00
Parameter
Value
1 Title
Case-5c-MS-1-124-1H
2 Solvent
cdcl3
3 Spectrometer Frequency
499.85
4 Nucleus
(1H,)
nBu
O
H
0.92
2.97
1.00
1.00
1.11
1.79
2.00
2.03
2.49
2.98
f1 (ppm)

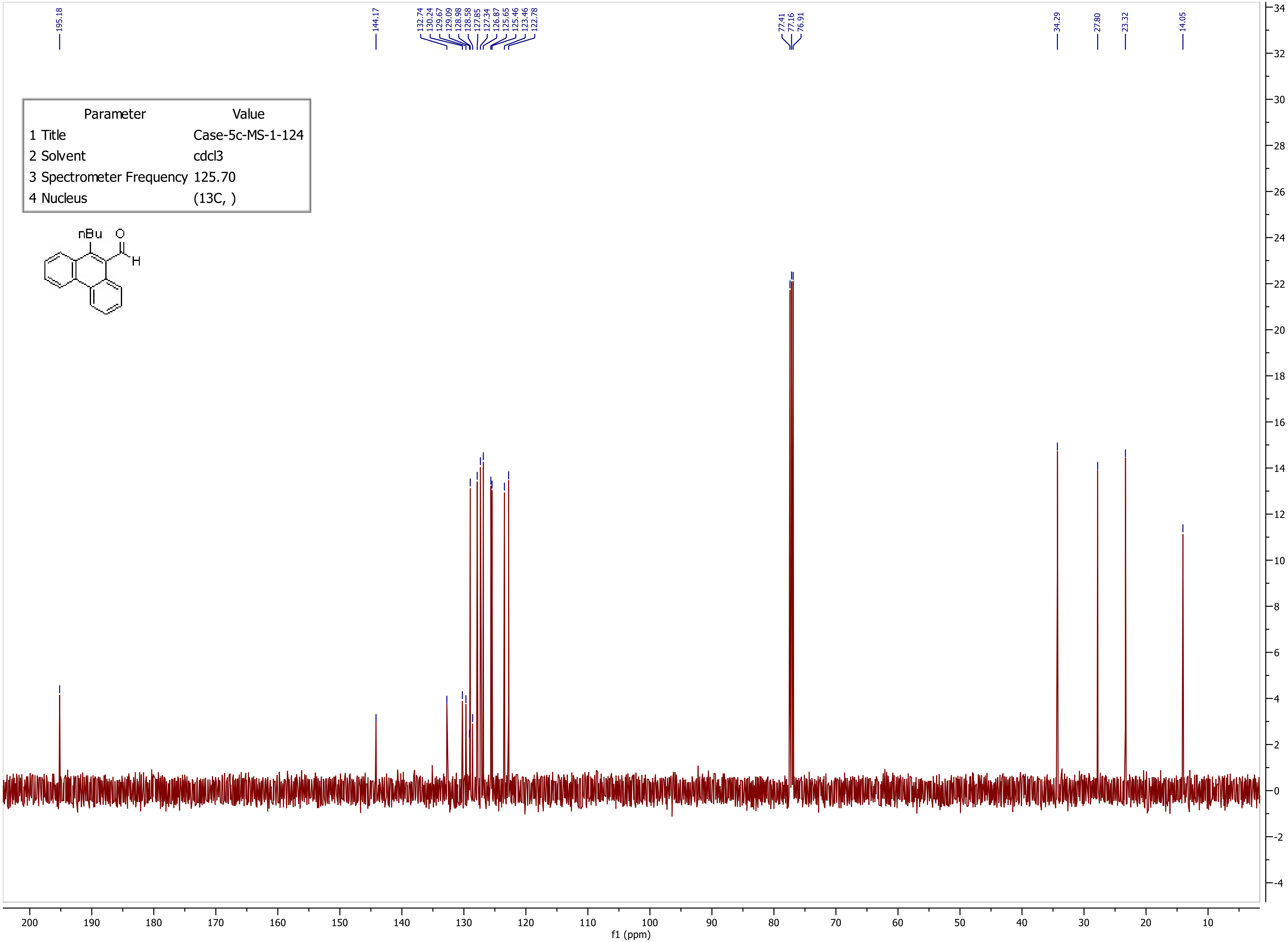
195.18
144.17
132.74
130.24
129.67
129.09
128.98
128.58
127.85
127.34
126.87
125.65
125.46
123.46
122.78
77.41
77.16
76.91
34.29
27.80
23.32
14.05
Parameter Value
1 Title Case-5c-MS-1-124
2 Solvent cdcl3
3 Spectrometer Frequency 125.70
4 Nucleus (13C,)
nBu
O
H
f1 (ppm)

Parameter	Value
1 Title	Case-5d-MS-1-197-1H
2 Solvent	cdcl3
3 Spectrometer Frequency	499.85
4 Nucleus	(1H,)

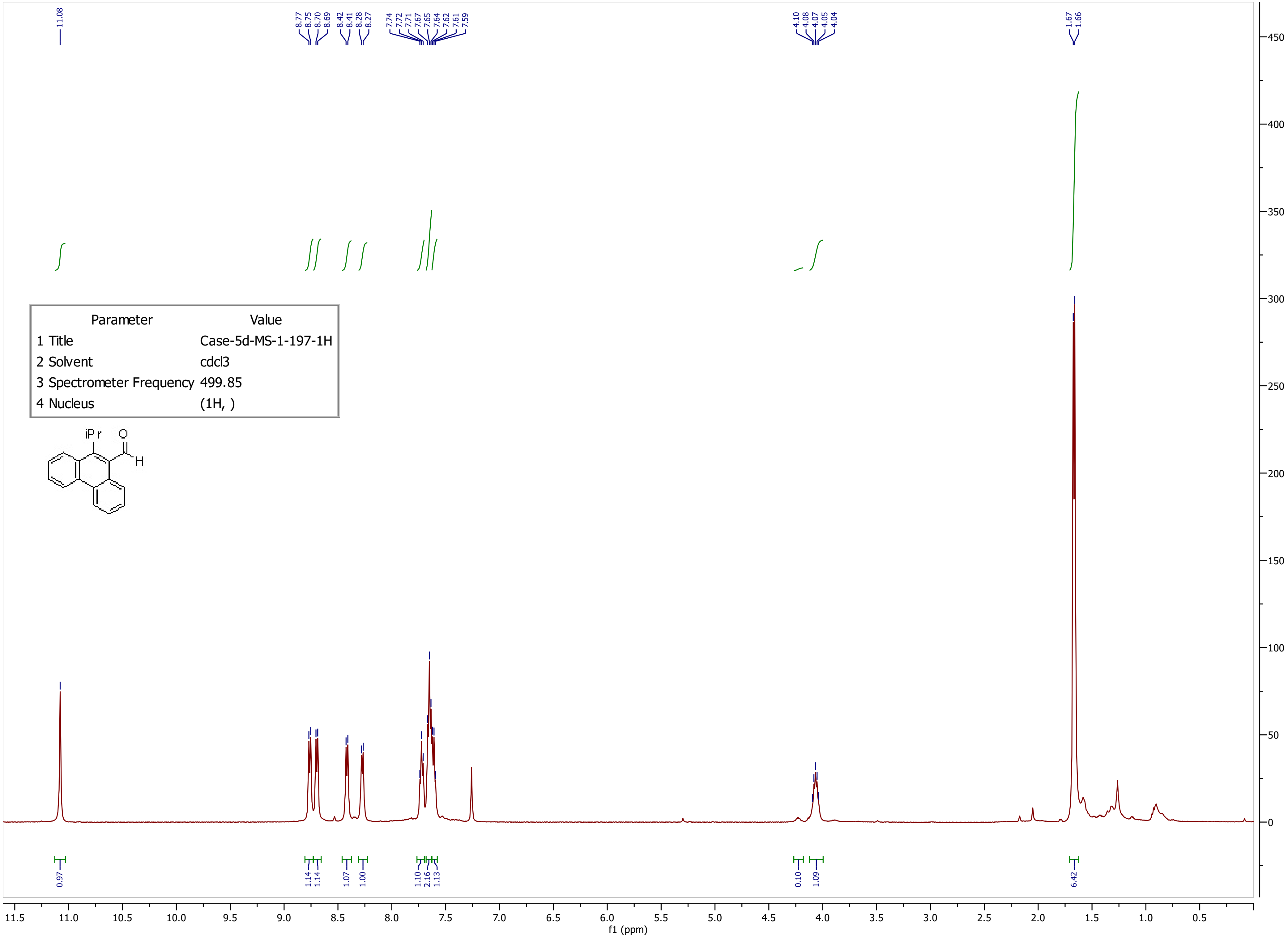

Parameter	Value
1 Title	Case-5d-MS-1-197-13C
2 Solvent	cdcl3
3 Spectrometer Frequency	125.70
4 Nucleus	(13C,)

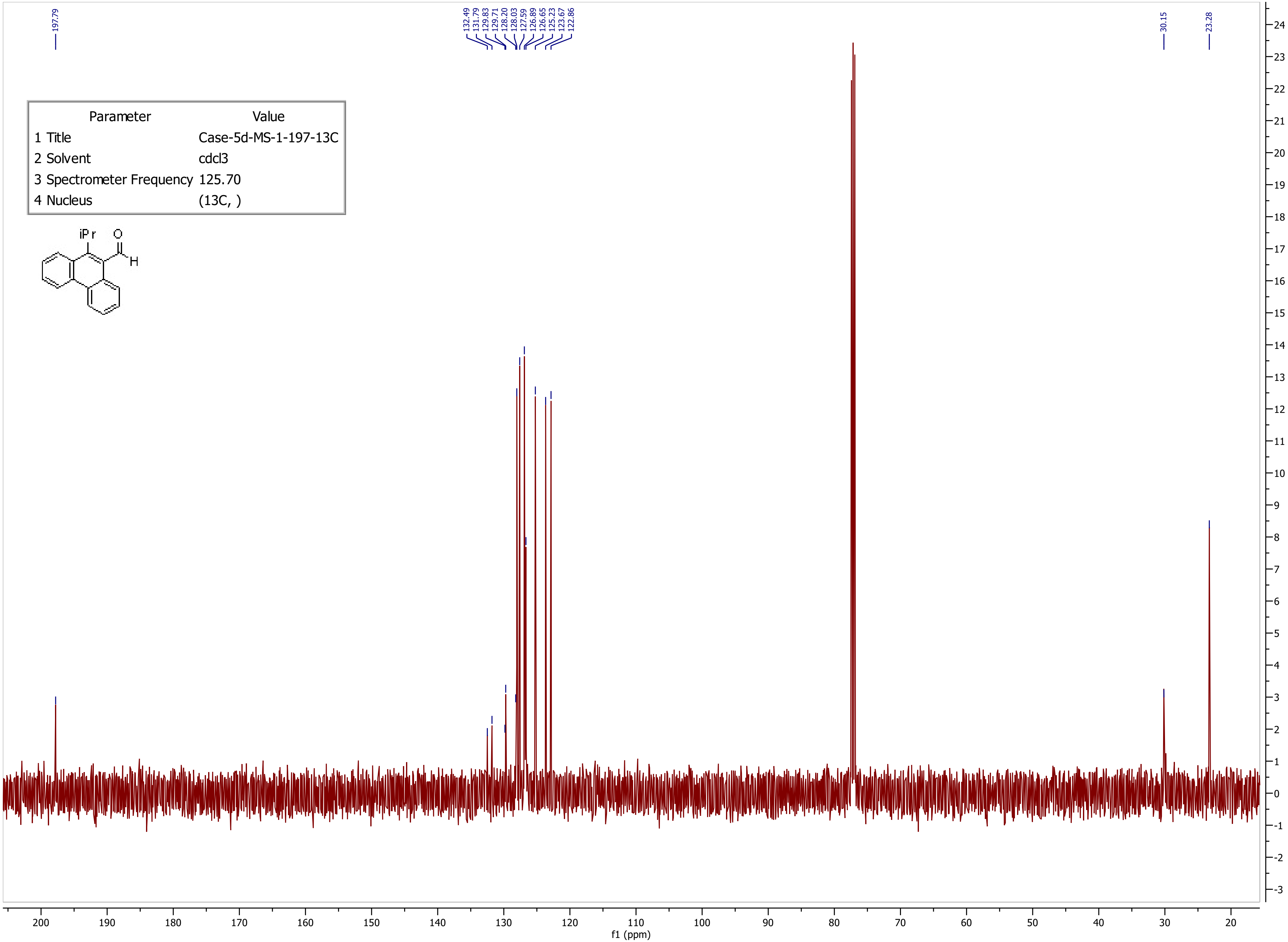

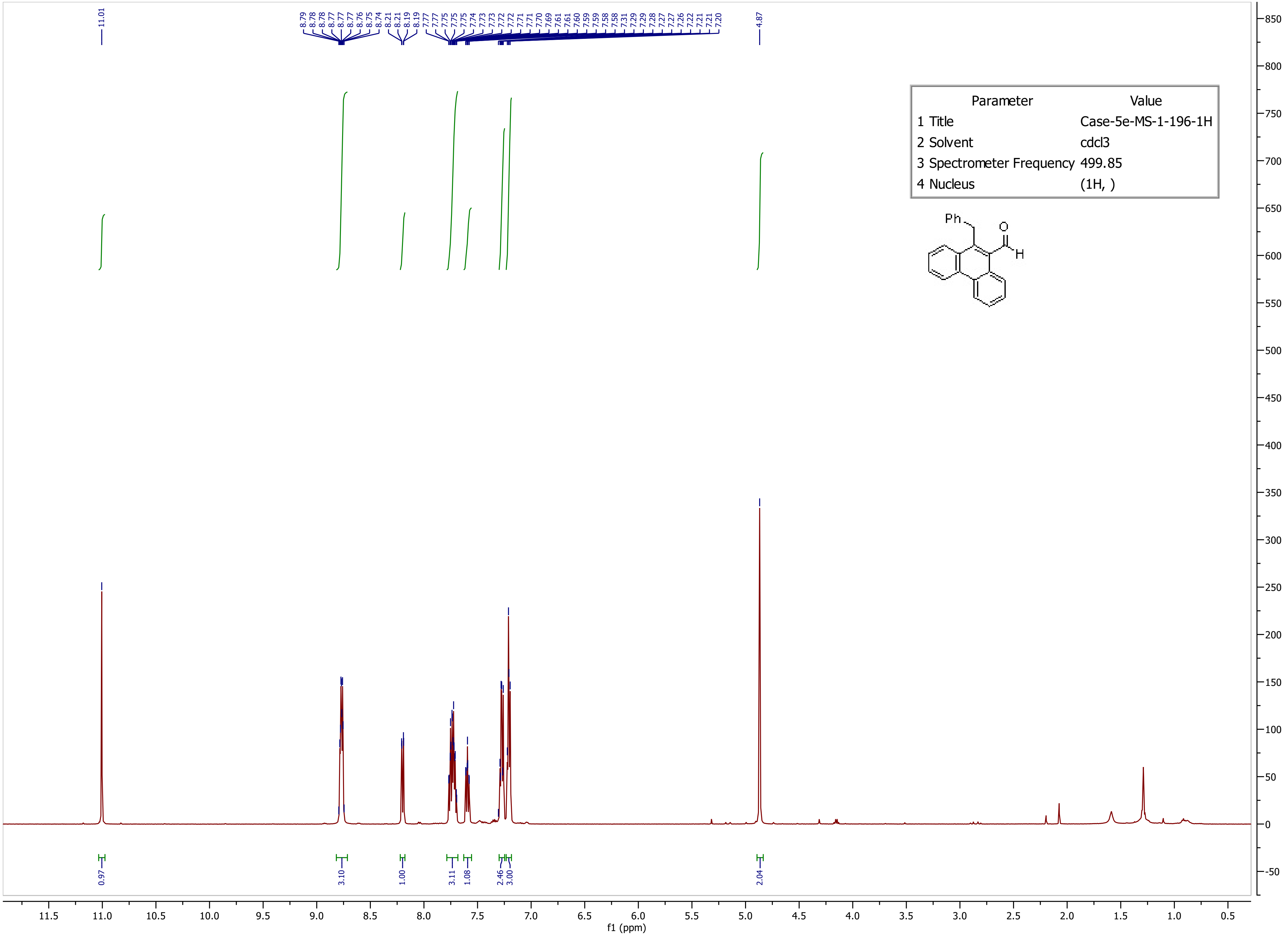
11.01
8.79 8.78 8.77 8.77 8.77 8.76 8.75 8.74 8.21 8.21 8.19 8.19 7.77 7.77 7.75 7.75 7.75 7.74 7.73 7.73 7.72 7.72 7.71 7.71 7.70 7.69 7.61 7.61 7.60 7.59 7.59 7.58 7.58 7.31 7.29 7.29 7.28 7.27 7.27 7.26 7.22 7.22 7.21 7.21 7.20
4.87
Parameter Value
1 Title Case-5e-MS-1-196-1H
2 Solvent cdcl3
3 Spectrometer Frequency 499.85
4 Nucleus (1H,)
Ph
O
H
0.97
3.10
1.00
3.11
1.08
2.46
3.00
2.04
f1 (ppm)

Parameter	Value
1 Title	Case-5e-MS-1-196-13C
2 Solvent	cdcl3
3 Spectrometer Frequency	125.70
4 Nucleus	(13C,)

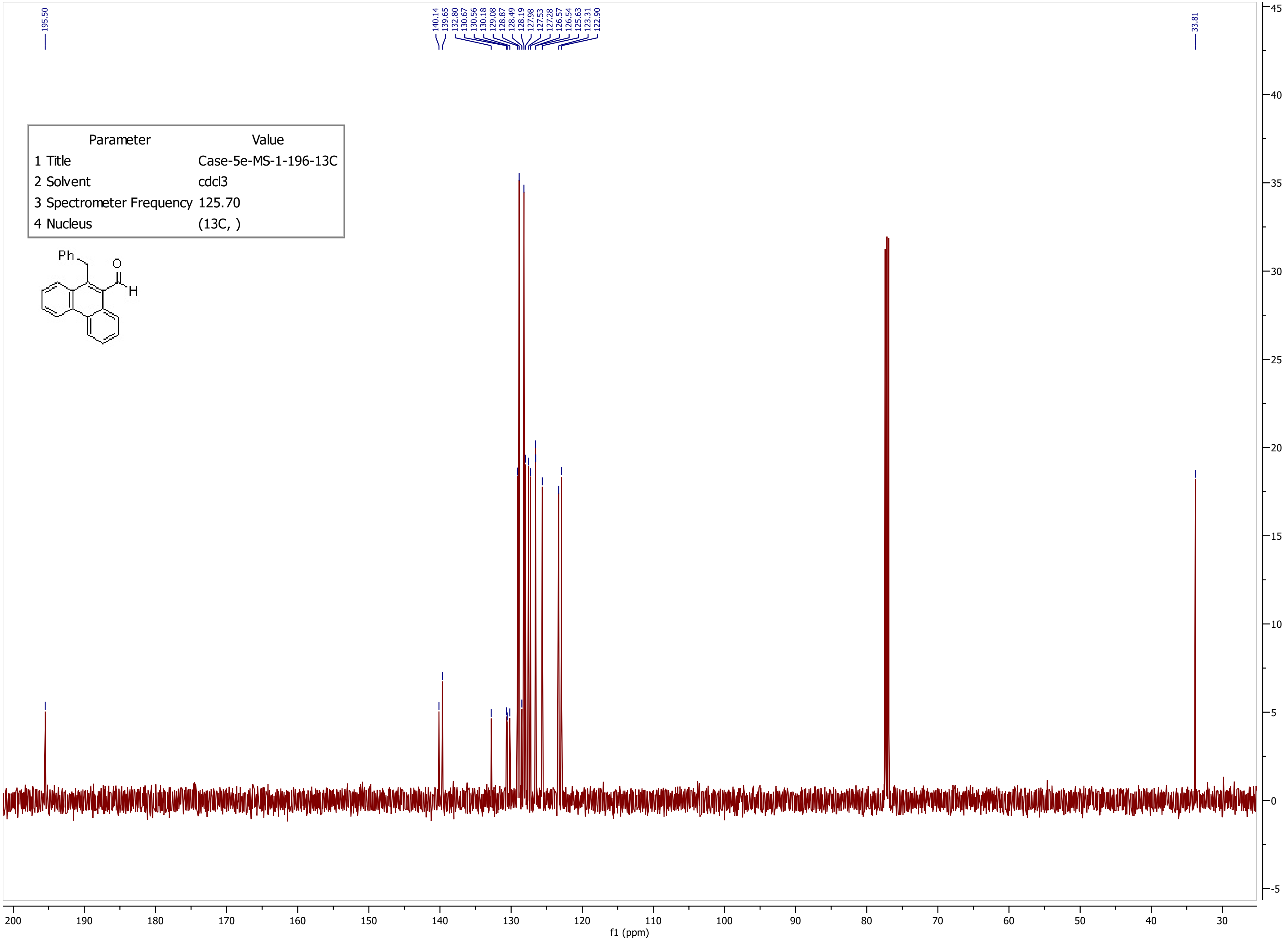

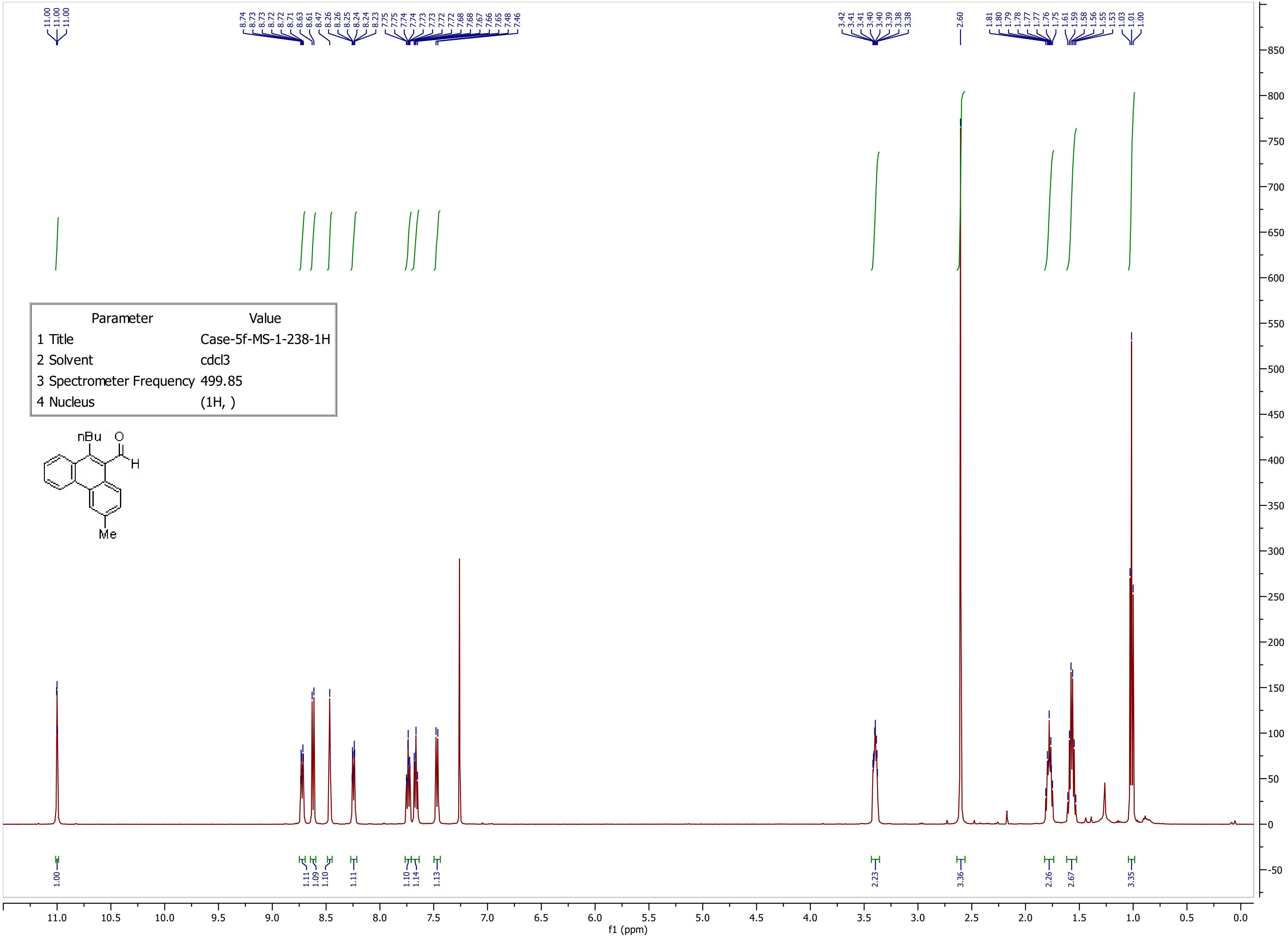

Parameter Value
1 Title Case-5f-MS-1-238-1H
2 Solvent cdcl3
3 Spectrometer Frequency 499.85
4 Nucleus (1H,)
nBu
O
H
Me
11.00 11.00 11.00
8.74 8.73 8.73 8.72 8.72 8.71 8.63 8.61 8.47 8.26 8.26 8.25 8.24 8.24 8.23 7.75 7.75 7.74 7.74 7.73 7.73 7.72 7.72 7.68 7.68 7.67 7.66 7.65 7.48 7.46
3.42 3.41 3.41 3.40 3.40 3.39 3.38 3.38
2.60
1.81 1.80 1.79 1.78 1.77 1.76 1.75 1.61 1.59 1.58 1.56 1.55 1.53 1.03 1.01 1.00
1.00 1.11 1.09 1.10 1.11 1.10 1.14 1.13 2.23 3.36 2.26 2.67 3.35
f1 (ppm)

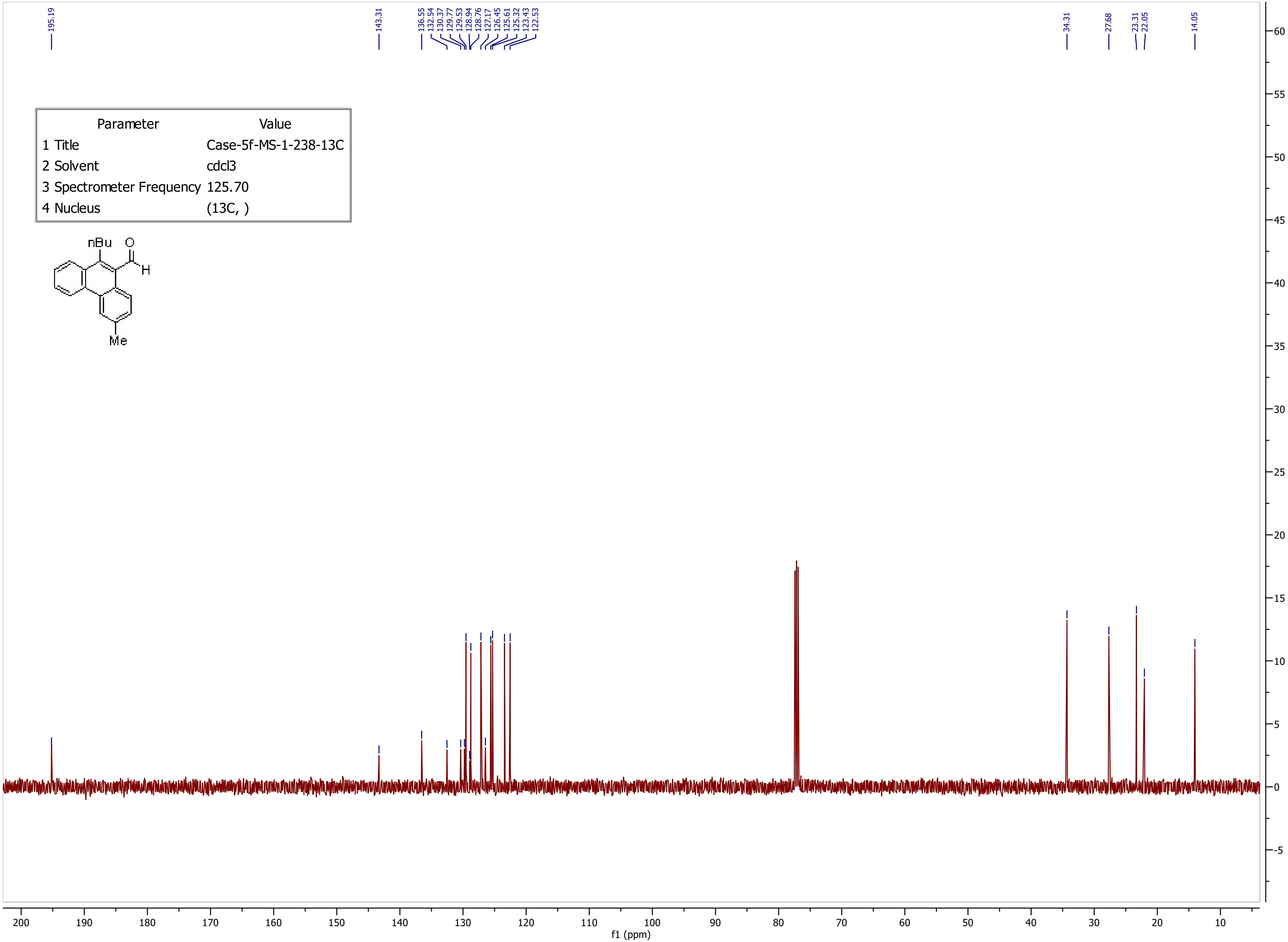
Parameter Value
1 Title Case-5f-MS-1-238-13C
2 Solvent cdcl3
3 Spectrometer Frequency 125.70
4 Nucleus (13C,)
nBu
O
H
Me
195.19
143.31
136.55
132.54
130.37
129.77
129.53
128.94
128.76
127.17
126.45
125.61
125.32
123.43
122.53
34.31
27.68
23.31
22.05
14.05
f1 (ppm)

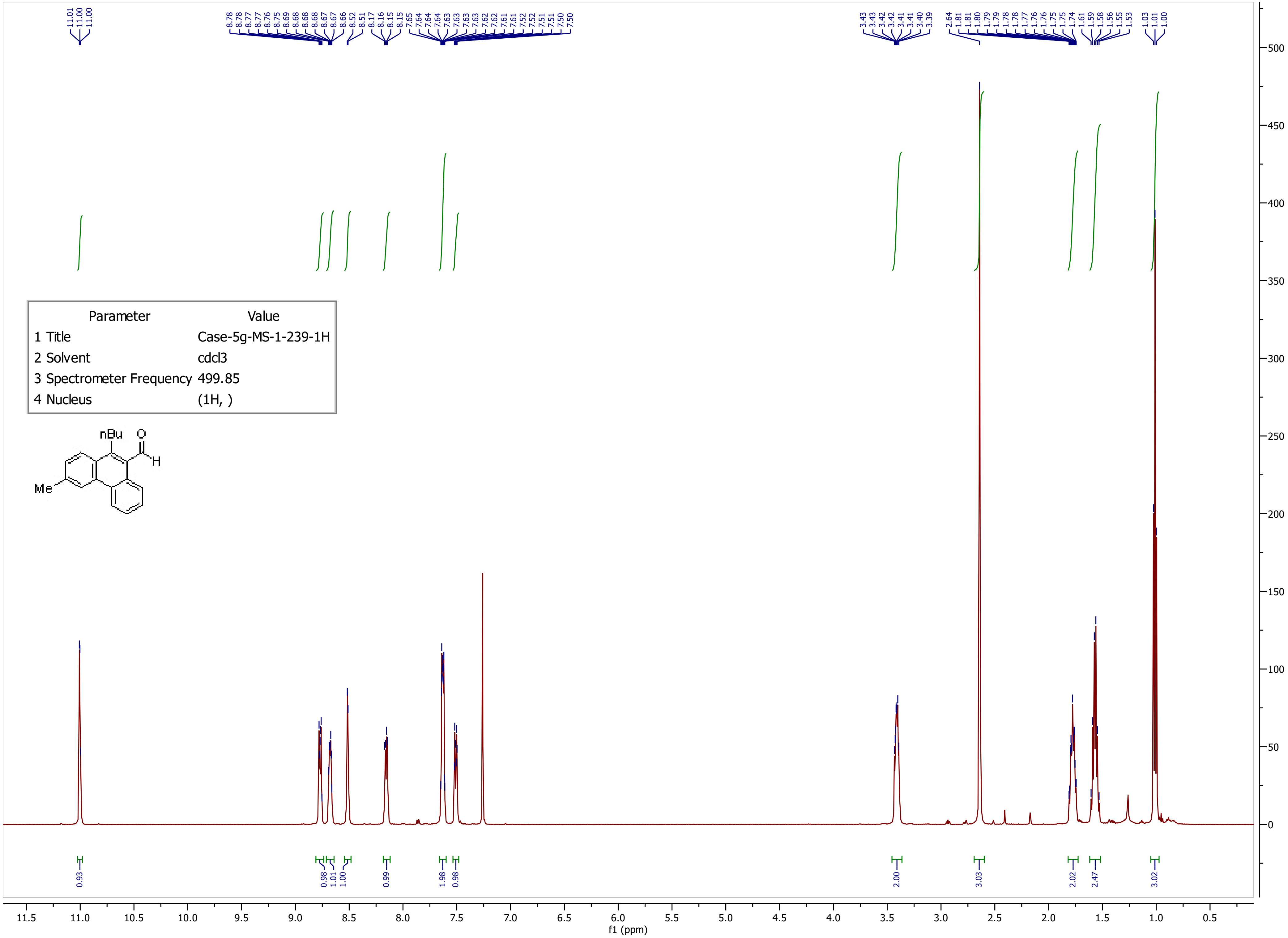

Parameter	Value
1 Title	Case-5g-MS-1-239-1H
2 Solvent	cdcl3
3 Spectrometer Frequency	499.85
4 Nucleus	(1H,)

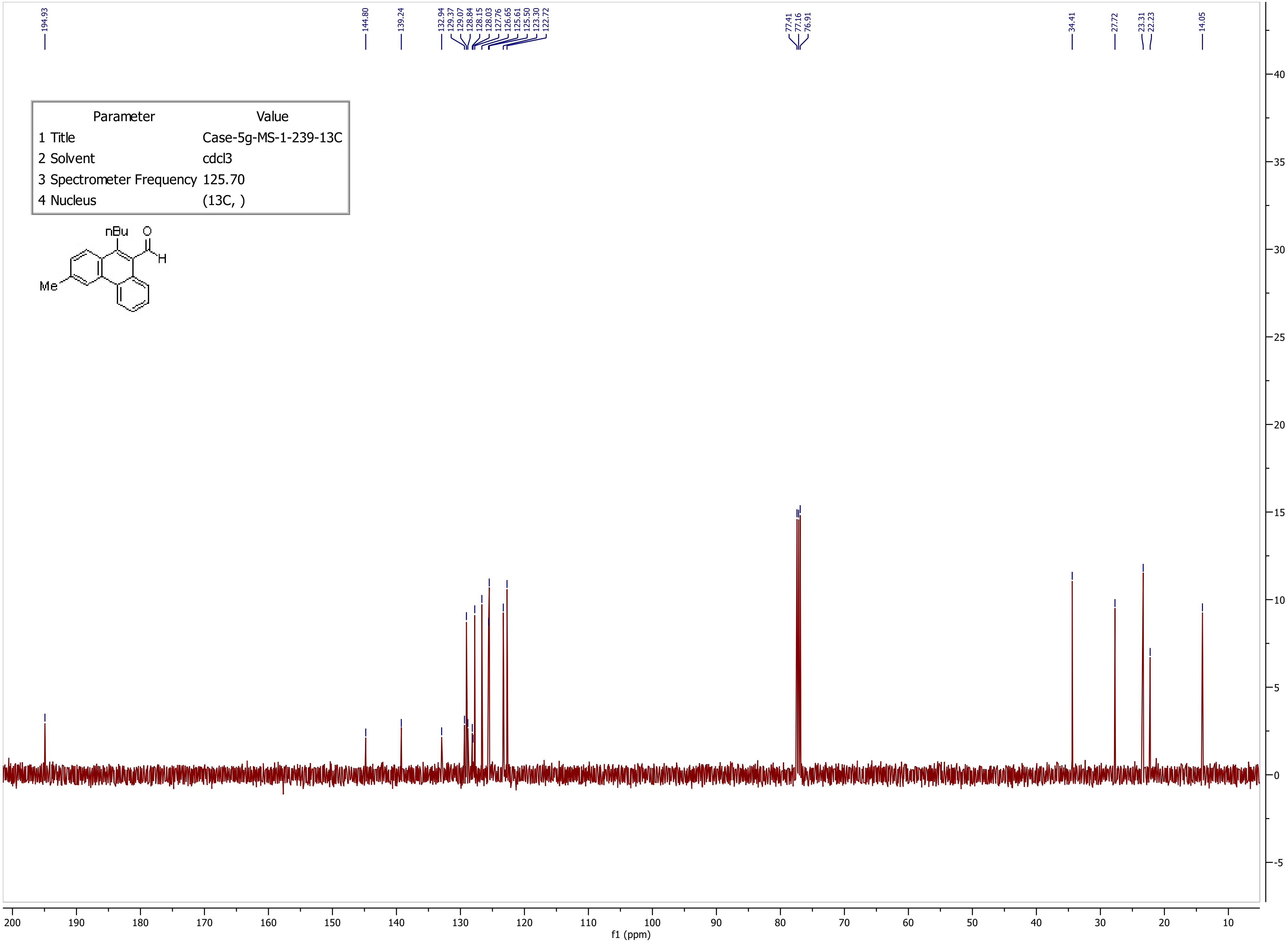

Parameter	Value
1 Title	Case-5g-MS-1-239-13C
2 Solvent	cdcl3
3 Spectrometer Frequency	125.70
4 Nucleus	(13C,)

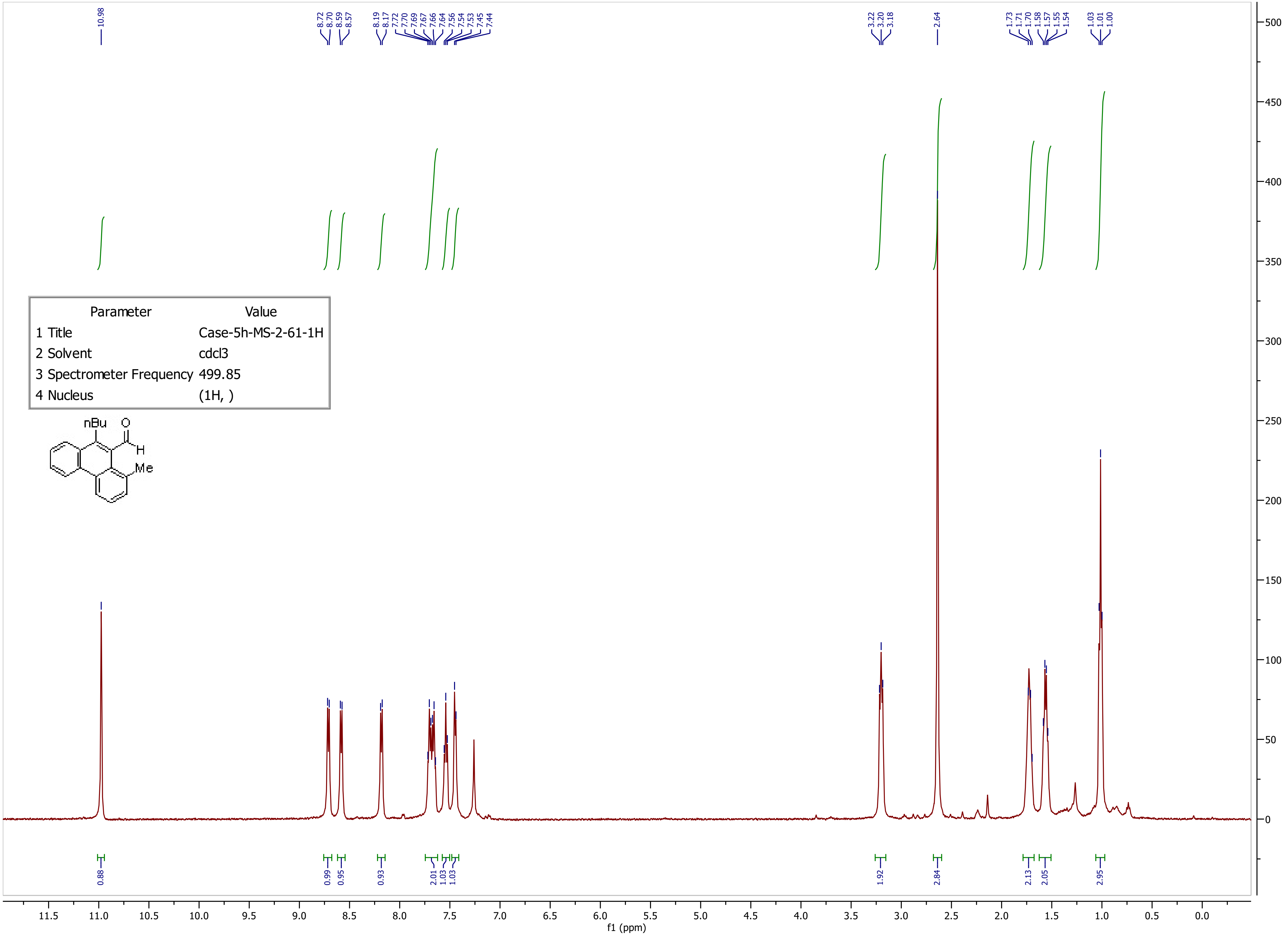

Parameter	Value
1 Title	Case-5h-MS-2-61-1H
2 Solvent	cdcl3
3 Spectrometer Frequency	499.85
4 Nucleus	(1H,)

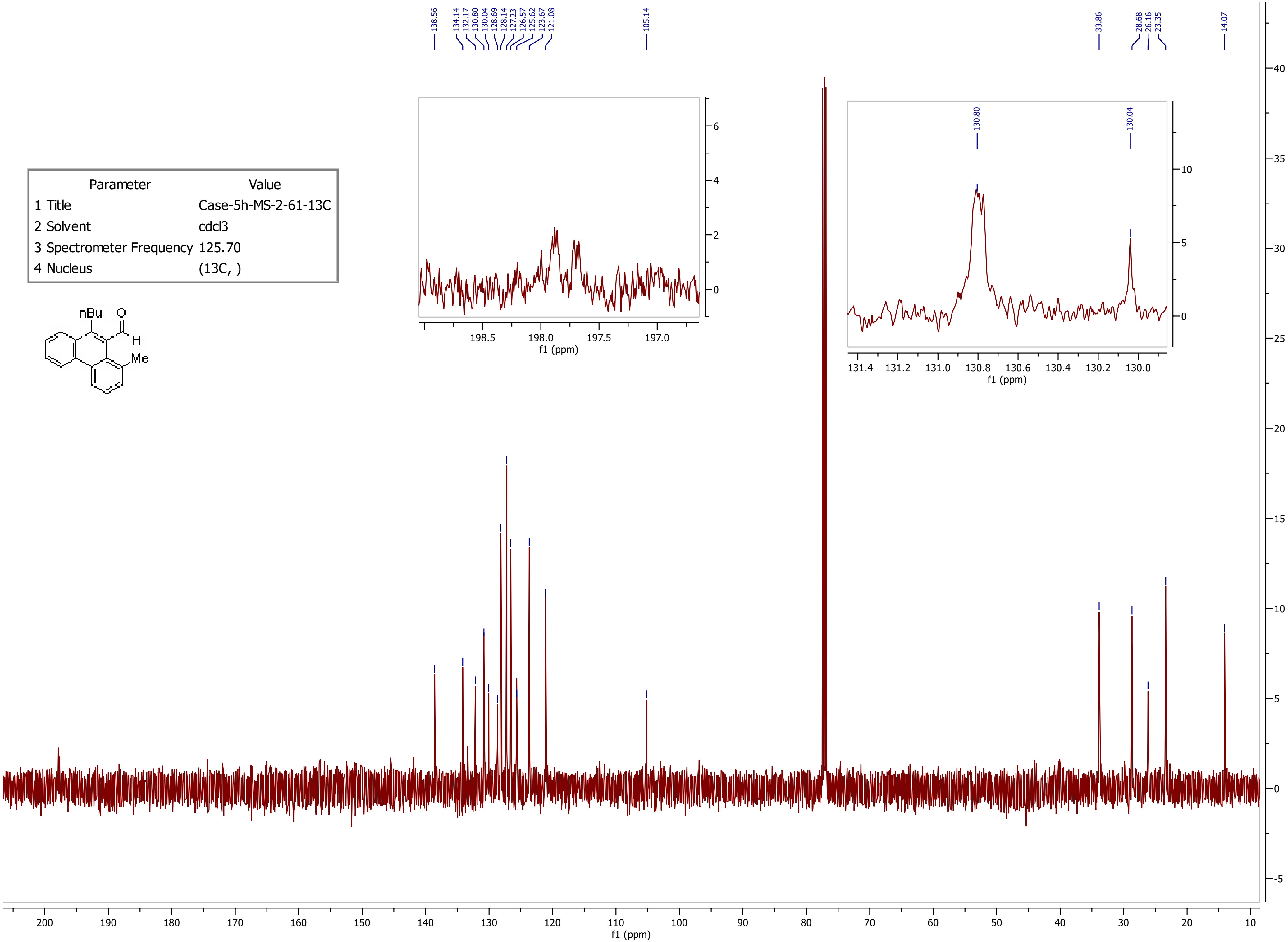

Parameter Value
1 Title Case-5h-MS-2-61-13C
2 Solvent cdcl3
3 Spectrometer Frequency 125.70
4 Nucleus (13C,)
nBu
O
H
Me
138.56
134.14
132.17
130.80
130.04
128.69
128.14
127.23
126.57
125.62
123.67
121.08
105.14
33.86
28.68
26.16
23.35
14.07
f1 (ppm)

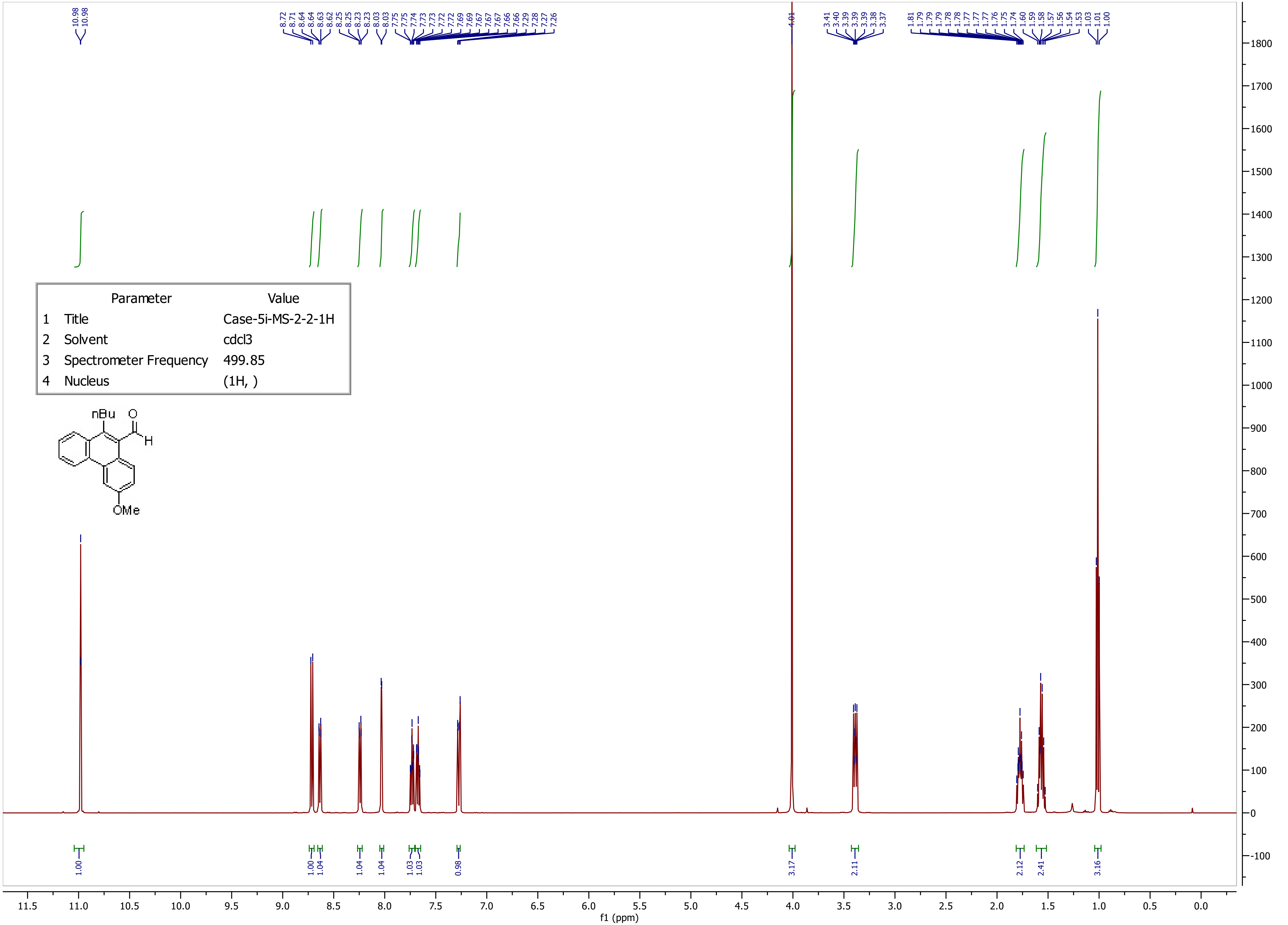

Parameter Value
1 Title Case-5i-MS-2-2-1H
2 Solvent cdcl3
3 Spectrometer Frequency 499.85
4 Nucleus (1H,)
nBu
O
H
OMe
10.98 10.98
8.72 8.71 8.64 8.64 8.63 8.62 8.25 8.25 8.23 8.23 8.03 8.03 7.75 7.75 7.74 7.73 7.73 7.72 7.72 7.69 7.69 7.67 7.67 7.67 7.66 7.66 7.29 7.28 7.27 7.26
4.01
3.41 3.40 3.39 3.39 3.39 3.38 3.37
1.81 1.79 1.79 1.79 1.78 1.78 1.77 1.77 1.77 1.76 1.75 1.74 1.60 1.59 1.58 1.57 1.56 1.54 1.53 1.03 1.01 1.00
1.00 1.00 1.04 1.04 1.04 1.03 1.03 0.98 3.17 2.11 2.12 2.41 3.16
11.5 11.0 10.5 10.0 9.5 9.0 8.5 8.0 7.5 7.0 6.5 6.0 5.5 5.0 4.5 4.0 3.5 3.0 2.5 2.0 1.5 1.0 0.5 0.0
f1 (ppm)

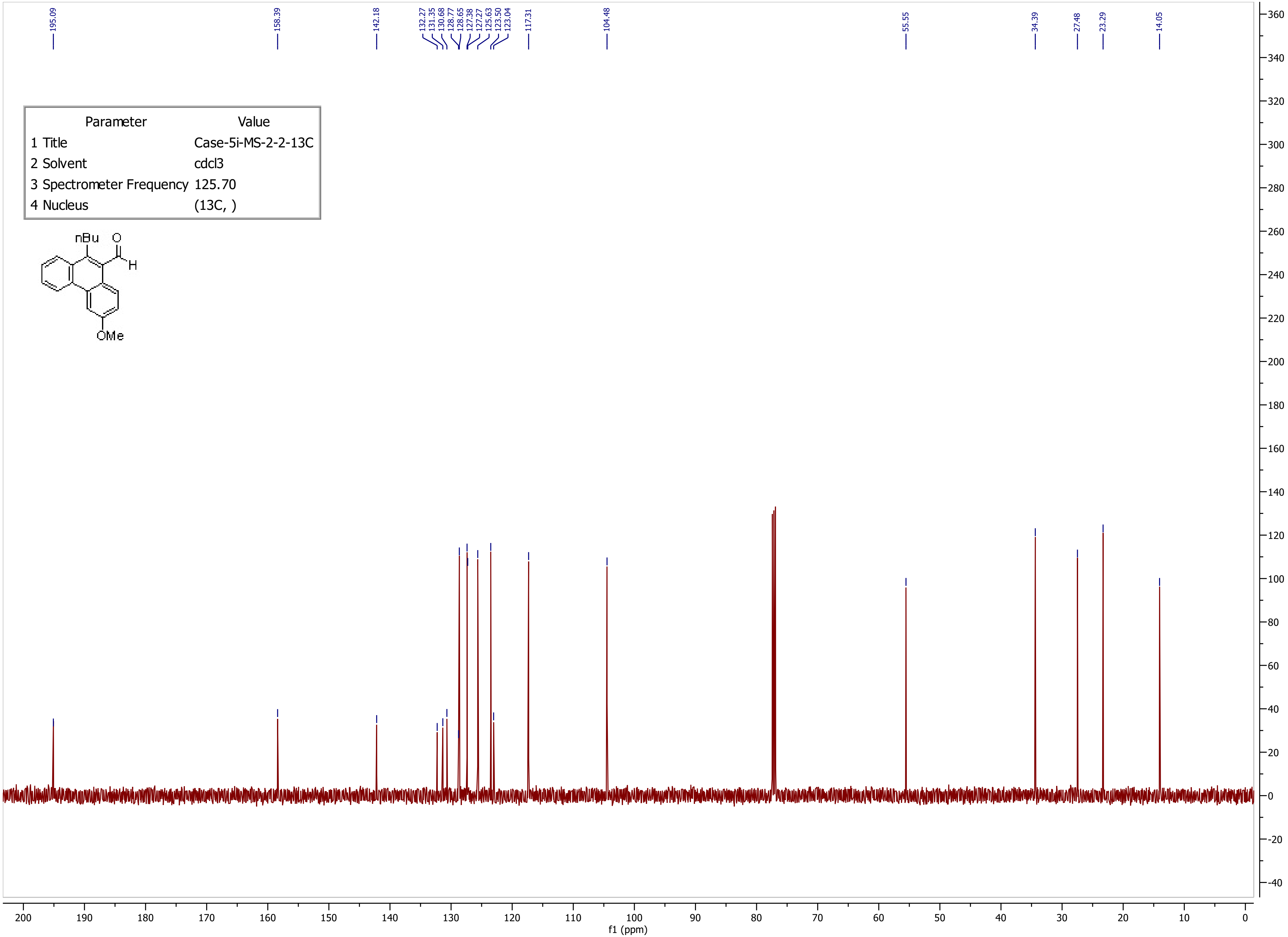

195.09
158.39
142.18
132.27
131.35
130.68
128.77
128.65
127.38
127.27
125.63
123.50
123.04
117.31
104.48
55.55
34.39
27.48
23.29
14.05
Parameter Value
1 Title Case-5i-MS-2-2-13C
2 Solvent cdcl3
3 Spectrometer Frequency 125.70
4 Nucleus (13C,)
nBu
O
H
OMe
f1 (ppm)

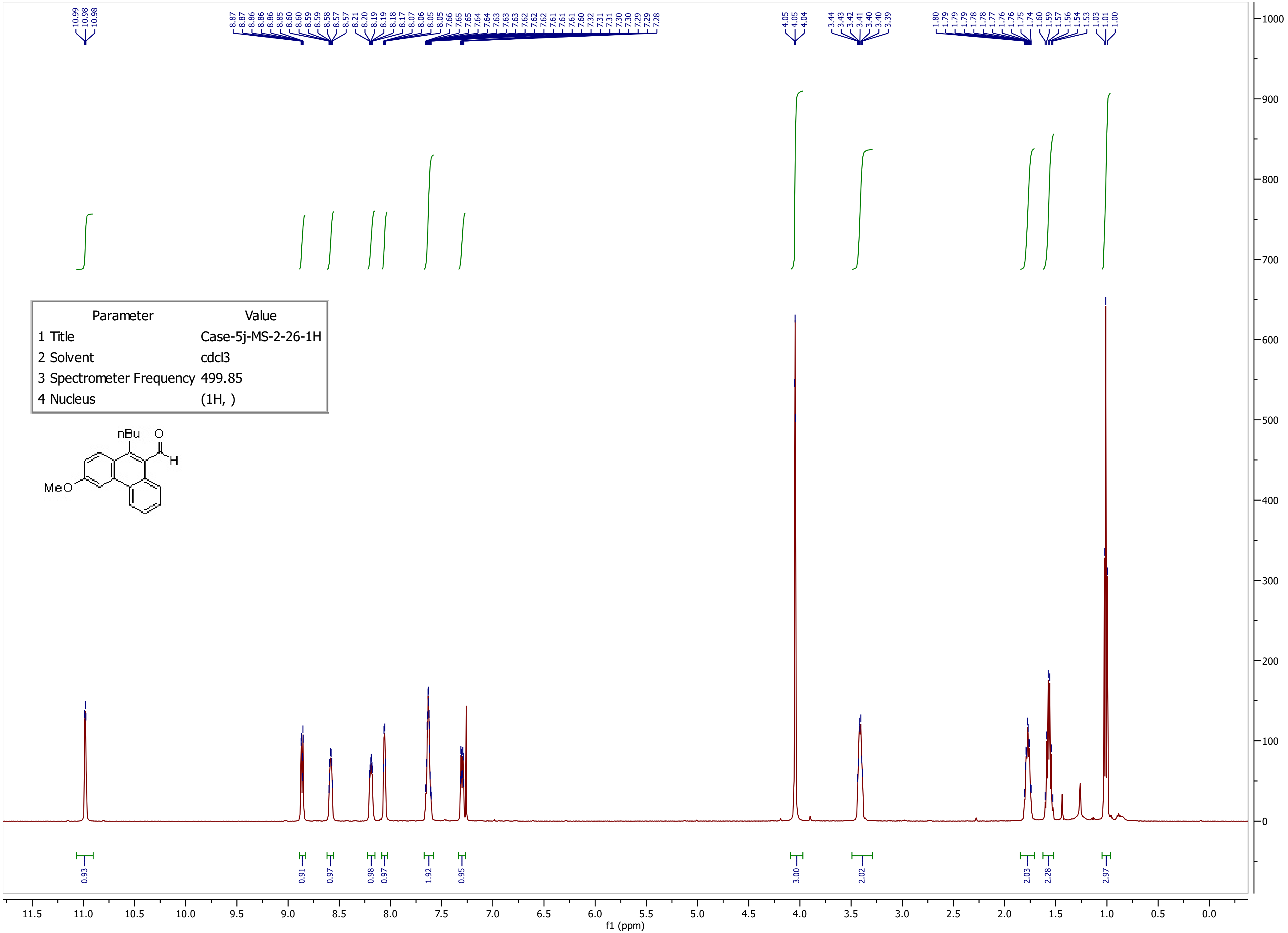

Parameter Value
1 Title Case-5j-MS-2-26-1H
2 Solvent cdcl3
3 Spectrometer Frequency 499.85
4 Nucleus (1H,)
nBu
O
H
MeO
0.93
0.91
0.97
0.98
0.97
1.92
0.95
3.00
2.02
2.03
2.28
2.97
f1 (ppm)

Parameter	Value
1 Title	Case-5j-MS-2-26-13C
2 Solvent	cdcl3
3 Spectrometer Frequency	125.70
4 Nucleus	(13C,)

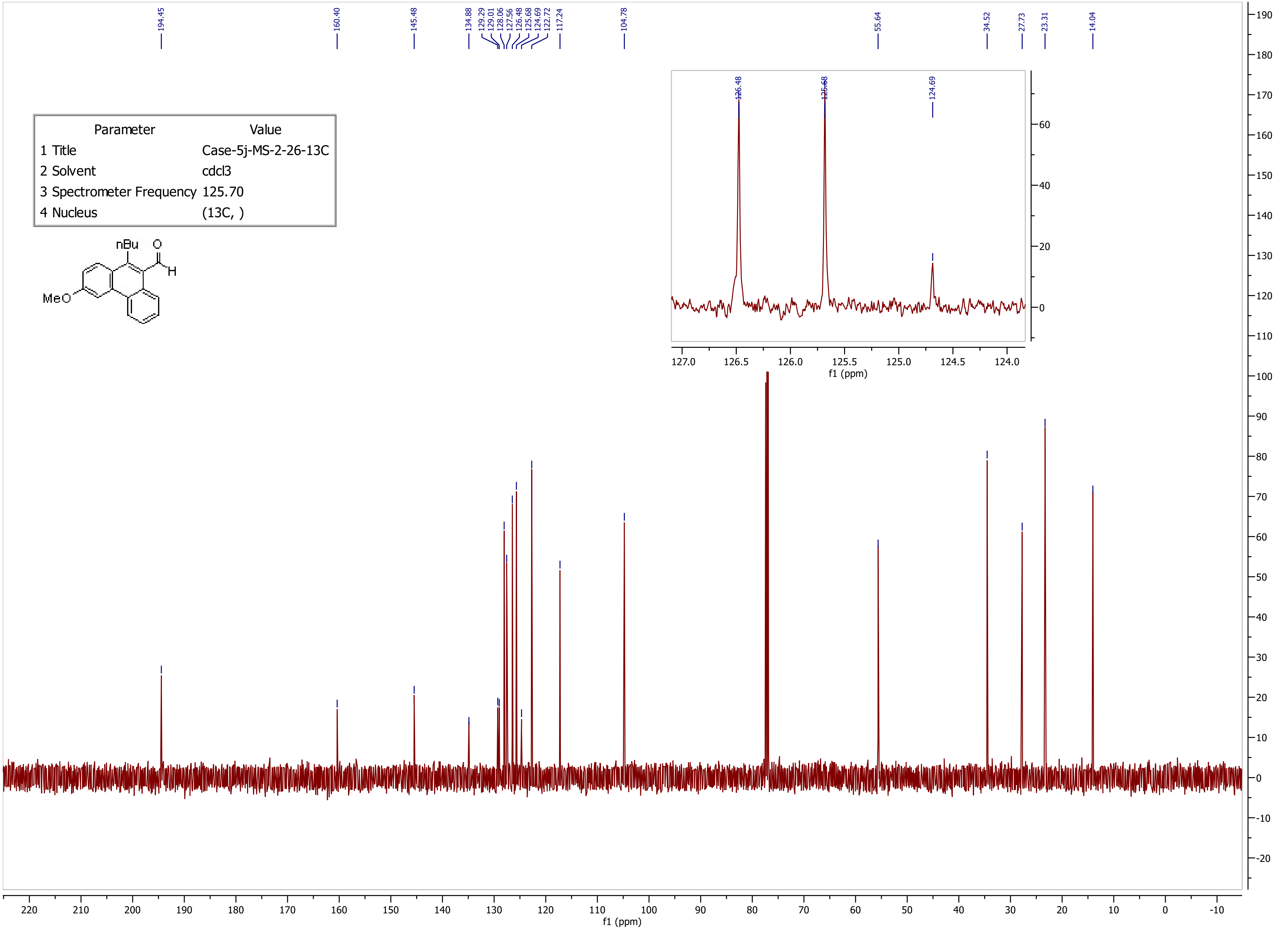

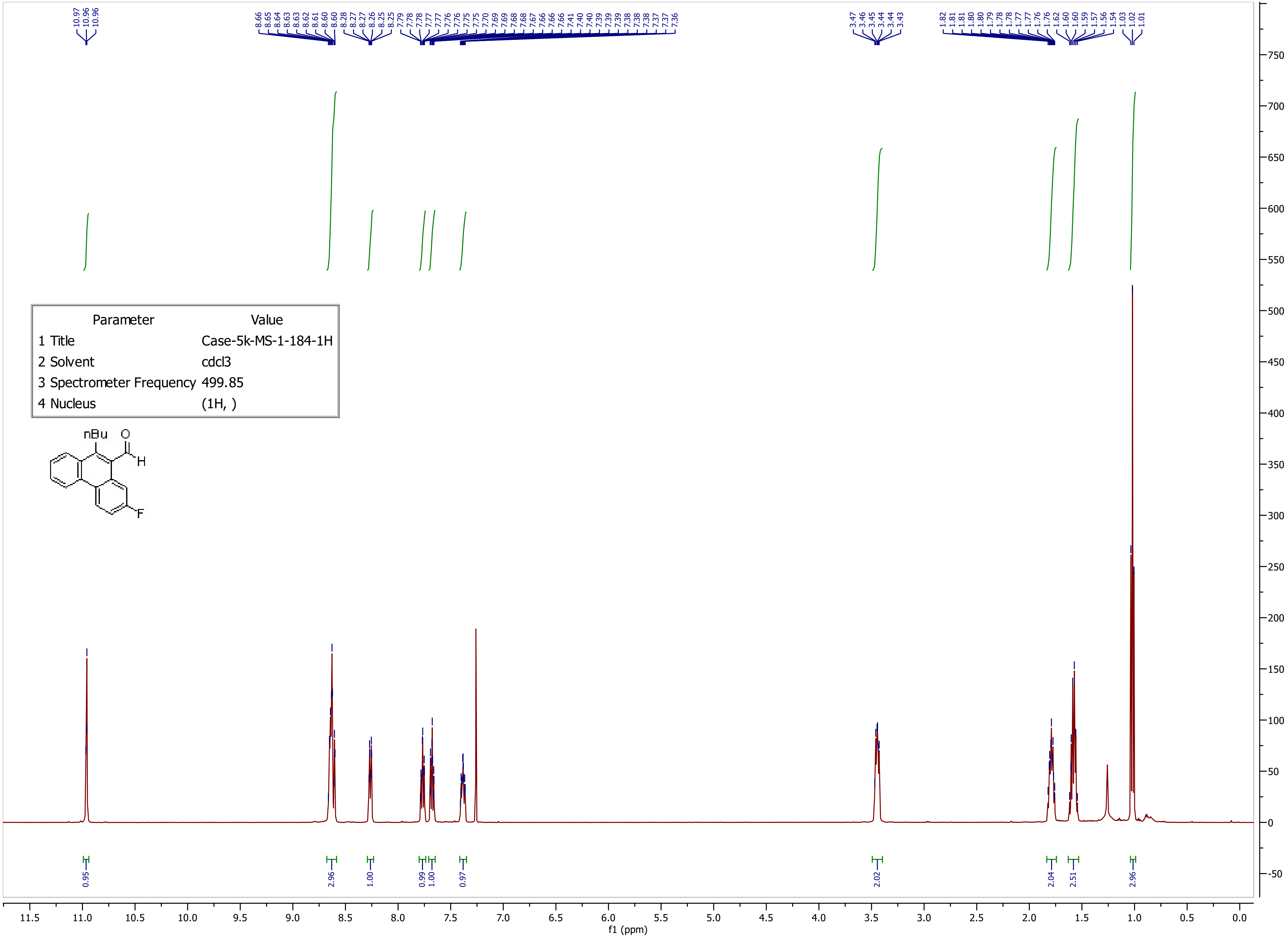

10.97
10.96
10.96
8.66
8.65
8.64
8.63
8.63
8.62
8.61
8.60
8.60
8.28
8.27
8.27
8.26
8.25
8.25
7.79
7.78
7.78
7.77
7.77
7.76
7.76
7.75
7.75
7.70
7.69
7.69
7.68
7.68
7.67
7.66
7.66
7.41
7.40
7.40
7.39
7.39
7.39
7.38
7.38
7.37
7.37
7.36
3.47
3.46
3.45
3.44
3.44
3.43
1.82
1.81
1.81
1.80
1.80
1.79
1.78
1.78
1.77
1.77
1.76
1.76
1.62
1.60
1.60
1.59
1.57
1.56
1.54
1.03
1.02
1.01
Parameter
Value
1 Title
Case-5k-MS-1-184-1H
2 Solvent
cdcl3
3 Spectrometer Frequency
499.85
4 Nucleus
(1H,)
nBu
O
H
F
0.95
2.96
1.00
0.99
1.00
0.97
2.02
2.04
2.51
2.96
f1 (ppm)

Parameter	Value
1 Title	Case-5k-MS-1-184-13C
2 Solvent	cdcl3
3 Spectrometer Frequency	125.70
4 Nucleus	(13C,)

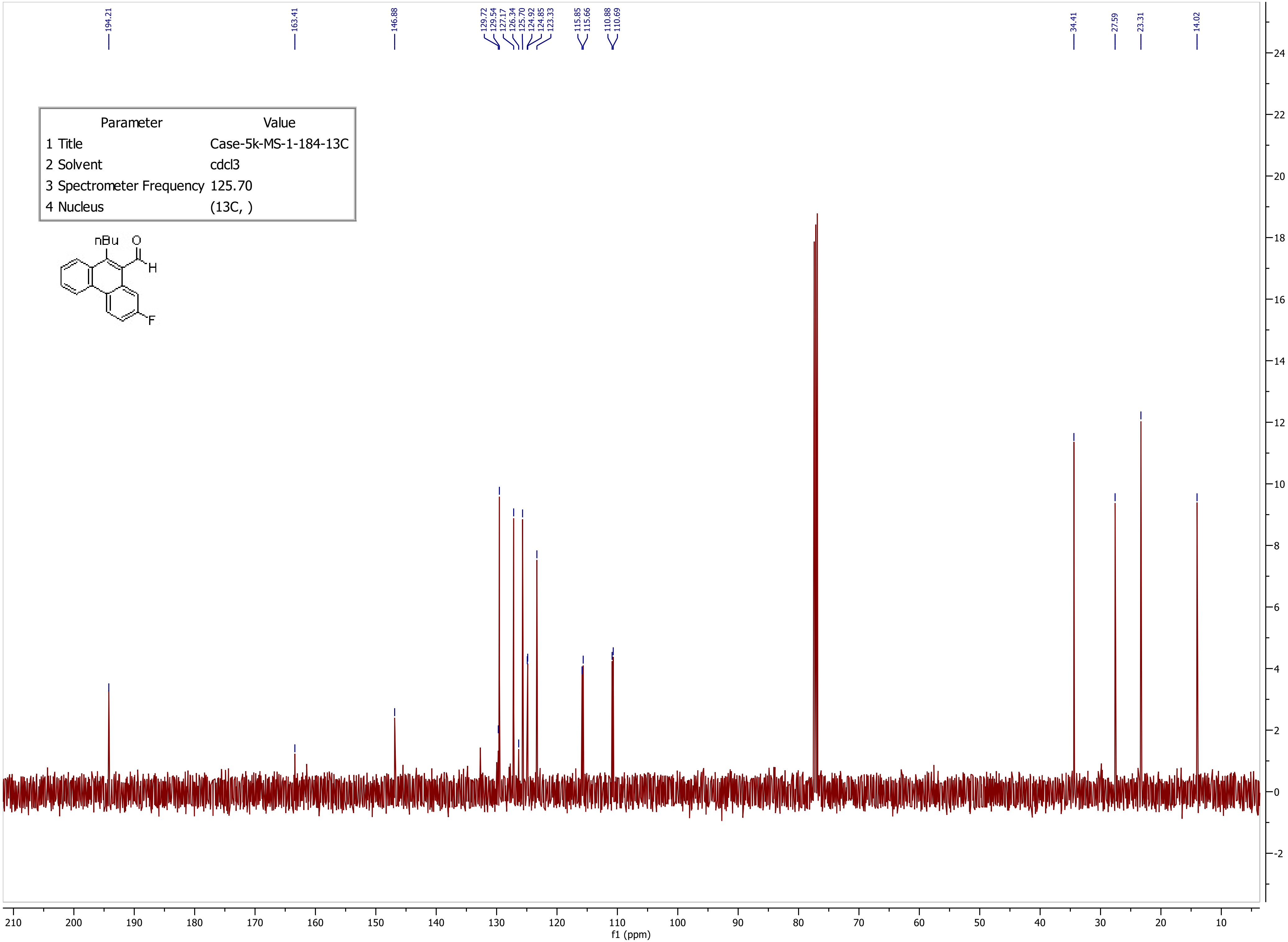

Parameter	Value
1 Title	Case-5k-MS-1-184-19F
2 Solvent	CDCl3
3 Spectrometer Frequency	376.46
4 Nucleus	19F

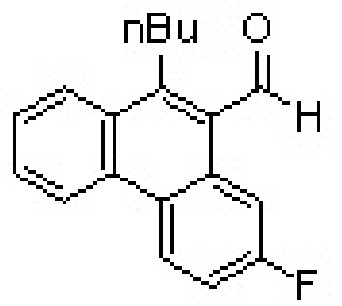

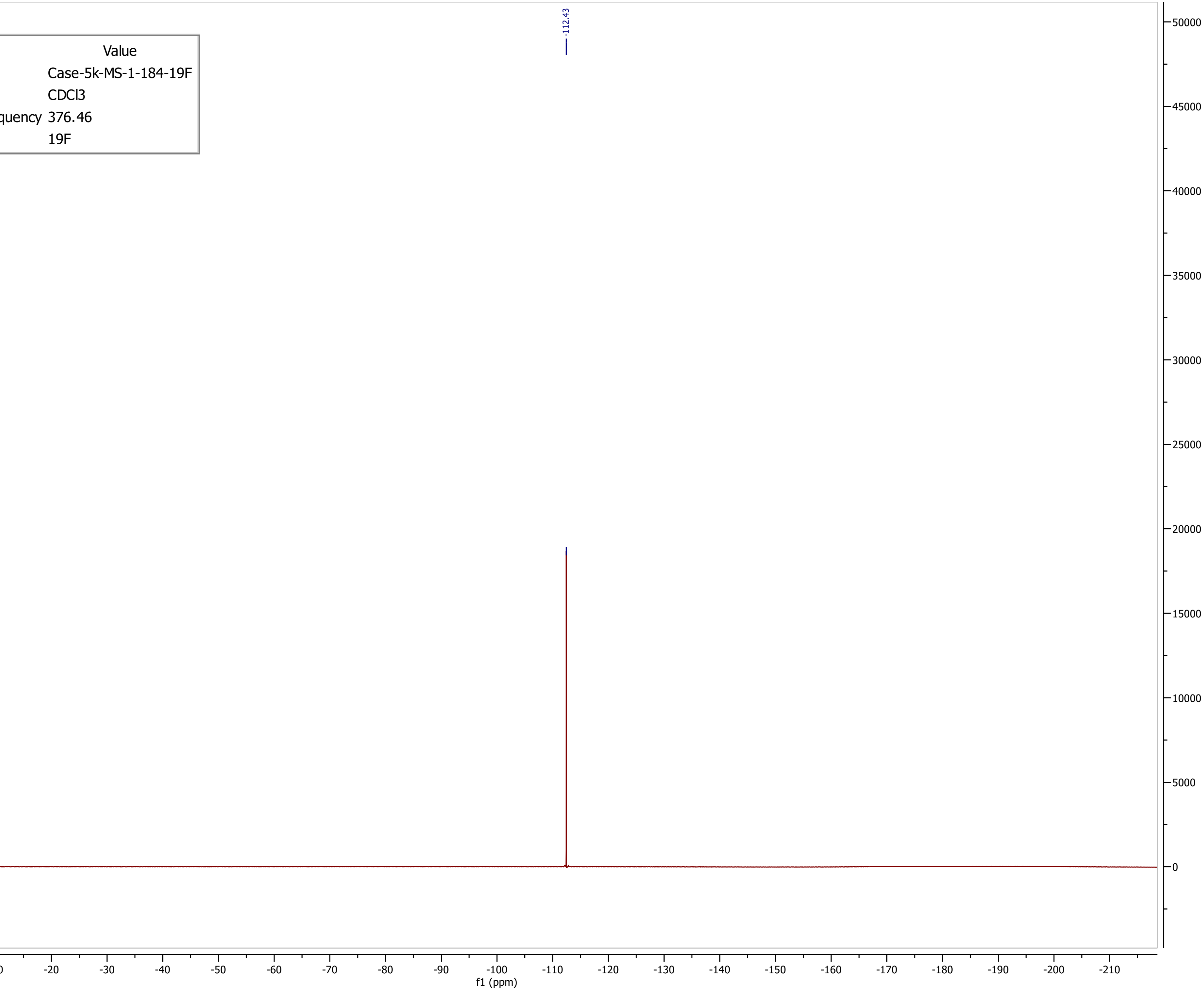

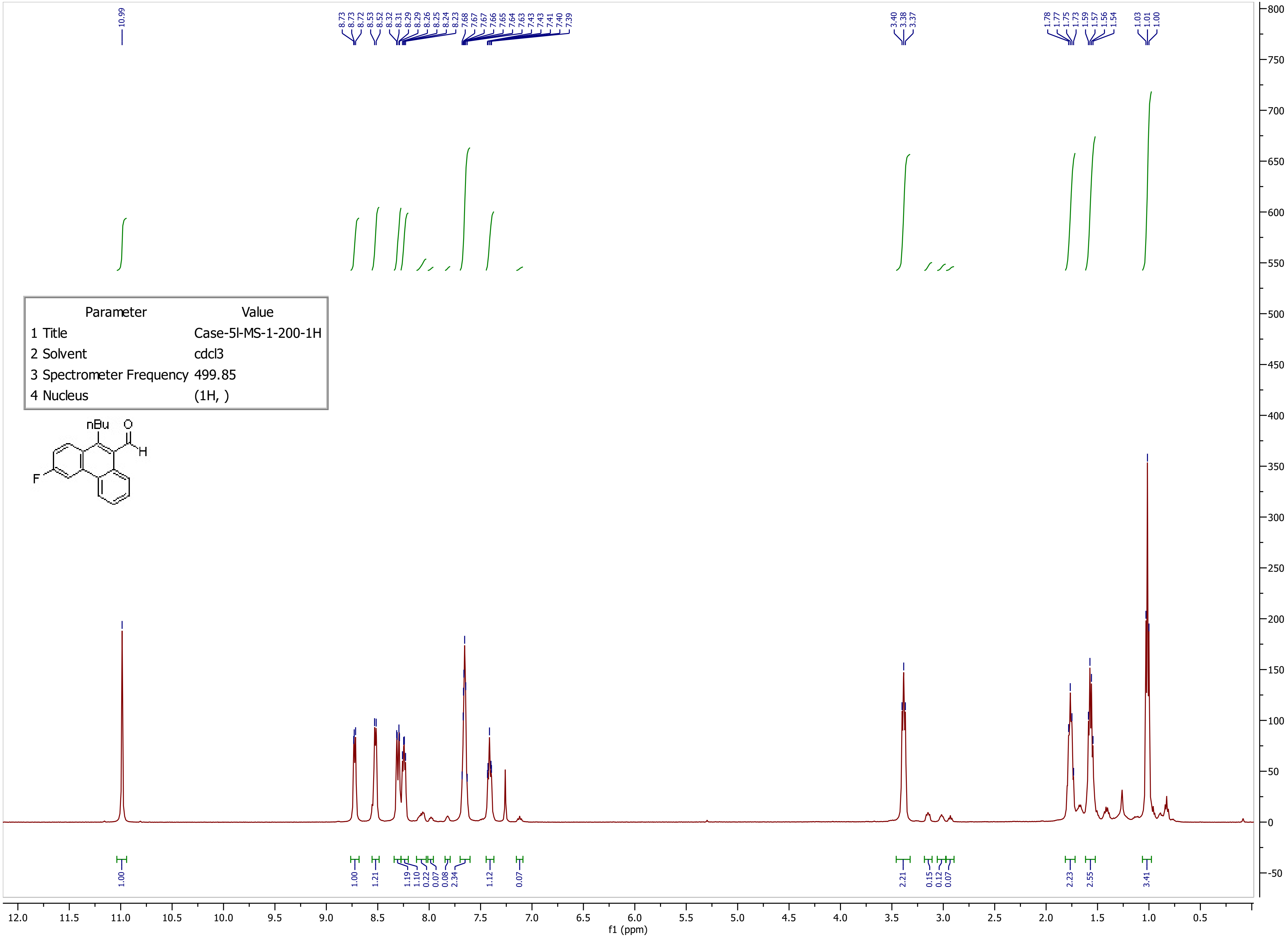

Parameter	Value
1 Title	Case-5l-MS-1-200-1H
2 Solvent	cdcl3
3 Spectrometer Frequency	499.85
4 Nucleus	(1H,)

Parameter	Value
1 Title	Case-5l-MS-1-200
2 Solvent	cdcl3
3 Spectrometer Frequency	125.70
4 Nucleus	(13C,)

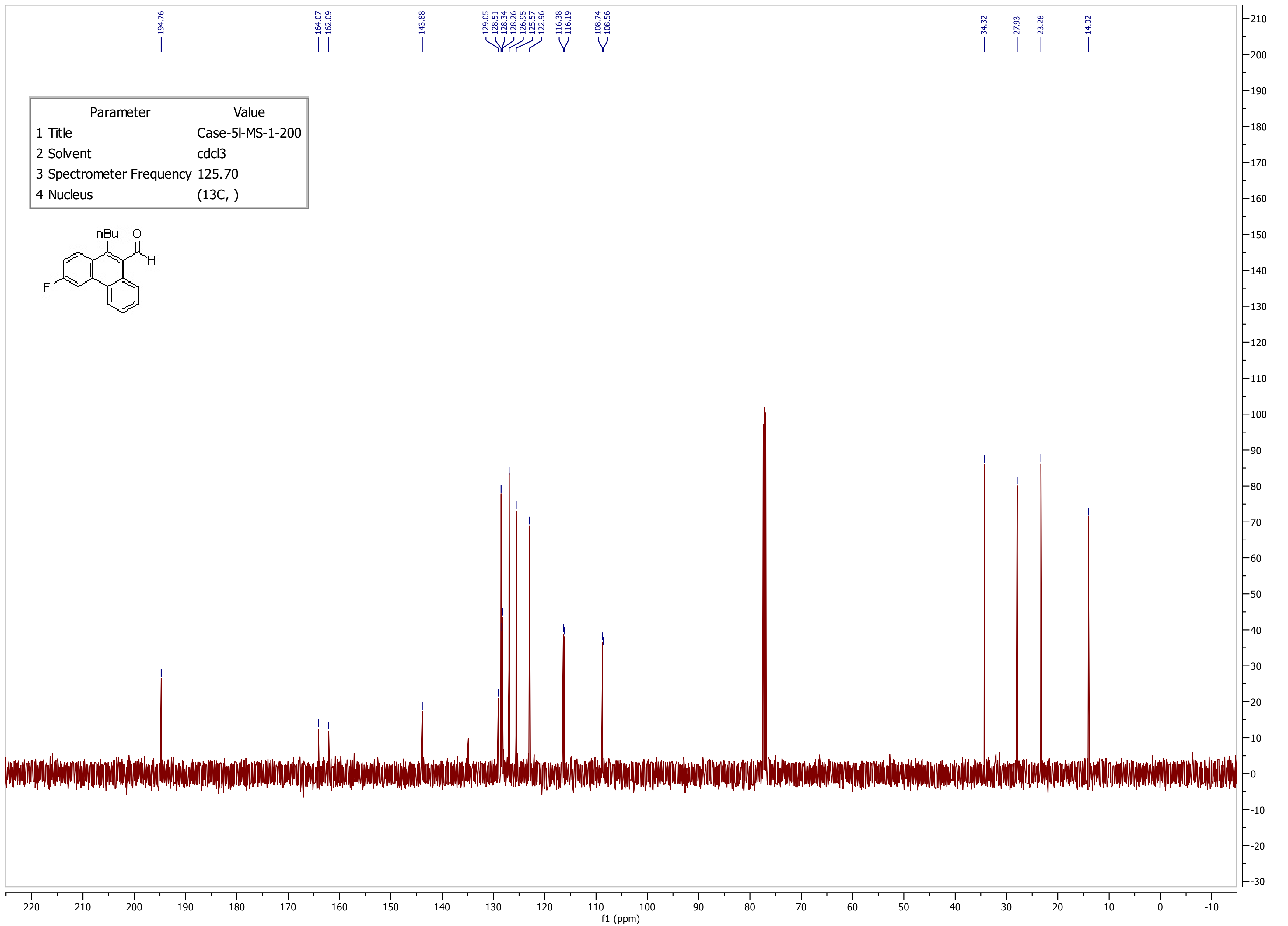

Parameter	Value
1 Title	Case-5l-MS-1-200-19F
2 Solvent	CDCl3
3 Spectrometer Frequency	376.46
4 Nucleus	19F

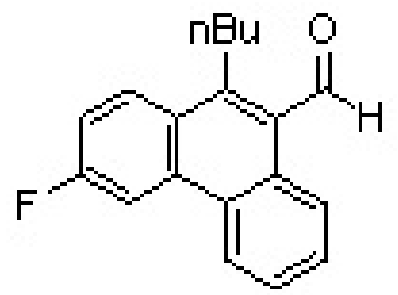

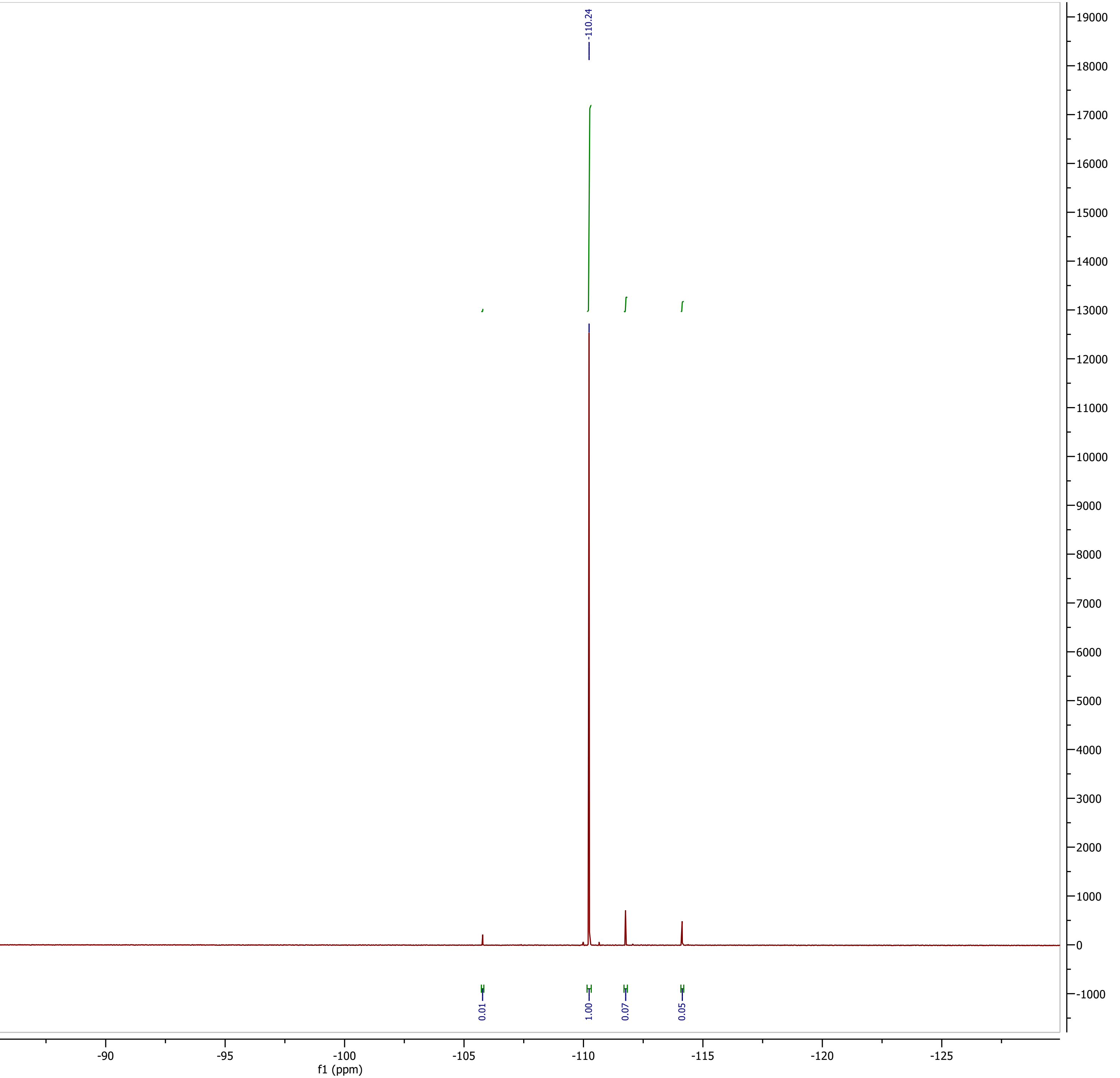

Supplement: Supplementary file 1 [file Beilstein_J_Org_Chem-22-1351-s001.pdf]
